# Stepwise Hydration Reveals Conformational Switching in Chiral Prolinol

**DOI:** 10.1021/jacs.5c13582

**Published:** 2025-12-11

**Authors:** Donatella Loru, Elena R. Alonso, Aran Insausti, Cristóbal Pérez, Luca Evangelisti, Juan L. Asensio, Francisco Corzana, Brooks H. Pate, Emilio J. Cocinero, M. Eugenia Sanz

**Affiliations:** † Department of Chemistry, King’s College London, London SE1 1DB, U.K.; ‡ Departamento de Química Física, Universidad del País Vasco (EHU), Instituto Biofisika (CSIC/EHU), Campus de Leioa, Ap. 644, 48080 Bilbao, Spain; § Department of Chemistry, University of Virginia, Charlottesville, Virginia 22904-4319, United States; ∥ Instituto de Química Orgánica General (IQOG-CSIC), Madrid 28006, Spain; ⊥ Departamento de Química and Instituto de Investigación en Química (IQUR), Universidad de La Rioja, Logroño 26006, Spain

## Abstract

Understanding the interactions of chiral molecules with water is crucial, given the central role that water plays in chemical and biological processes. We report the investigation of the amino alcohol prolinol, a widely used chiral catalyst and auxiliary in asymmetric synthesis, and its interactions with one to three water molecules by applying broadband rotational spectroscopy. Bare prolinol adopts two low-energy conformations stabilized by an intramolecular O–H···N hydrogen bond. Upon complexation with a single water molecule, four prolinol–H_2_O isomers are identified, showing addition and insertion structures, where the original prolinol conformations are conserved. Notably, complexation with two and three water molecules induces prolinol to adopt its highest energy conformations, which lie more than 9.5 kJ mol^–1^ above the global minimum and feature an intramolecular N–H···O hydrogen bond. In prolinol–(H_2_O)_2,3_, water acts as a conformational switch for prolinol, binding to both the amino and hydroxyl groups. Combined NMR studies and molecular dynamics simulations reveal that, in bulk water, prolinol exists as a highly flexible conformational ensemble, with no evidence of a stable intramolecular hydrogen bond, and mainly samples the same conformational space as that displayed in prolinol–(H_2_O)_2,3_. Our results illustrate how stepwise hydration proceeds and reveal the profound changes that water can induce in flexible chiral molecules. These findings provide a solid foundation for future experiments and modeling of solvation-induced processes.

## Graphical abstract

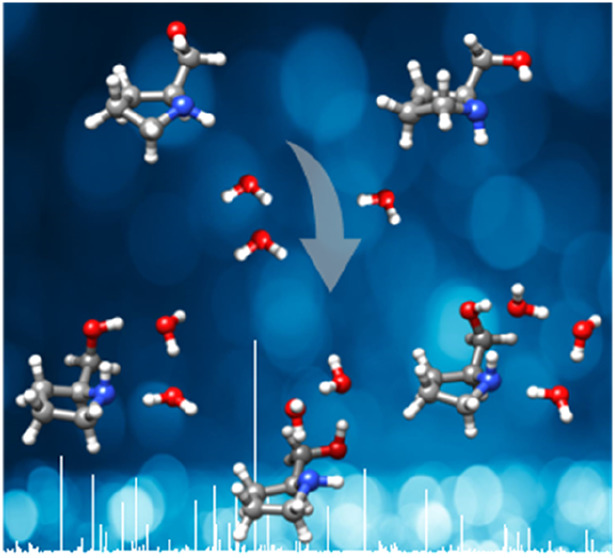

## Introduction

Nowadays, water is recognized to play an active role in a wide range of biological and chemical processes. Water has been shown to participate in catalytic asymmetric reactions,^1^ influence ligand binding,^2^ facilitate proton transport through hydrogen-bonded chains,^3^ and induce self-assembly and protein folding.^4,5^ Many of these activities involve interactions with chiral molecules, where water can modulate or even determine chiral behavior. For instance, water has been shown to produce chirality amplification by boosting enantioselectivity in asymmetric reactions^6^ and to induce chirality inversion.^7^ Conversely, chiral solutes can transfer their chirality to the surrounding water molecules. Using chiral sum frequency generation (SFG) spectroscopy, macromolecules were recently found to impose a chiral topology on their first solvation shell that mirrors their own.^8–11^ Chirality transfer from small chiral molecules to water was also demonstrated by vibrational circular dichroism (VCD) and Raman optical activity (ROA) experiments.^12,13^ These experimental observations cannot be reproduced by using implicit water models and require explicit consideration of individual water molecules. In fact, specific solute–water clusters in the bulk, which surprisingly only contain a few water molecules, have been shown to account for the observed VCD and ROA spectral features.^13^ Investigating clusters between chiral solutes and water is thus essential to unravel how water modulates chirality and understand its specific role in solute–solvent interactions.

An important class of chiral molecules is that of vicinal or β-amino alcohols, which feature hydroxyl and amino groups in adjacent carbon atoms. These compounds are pivotal building blocks in many biologically active molecules, including natural products and synthetic drugs,^14^ and are widely used as catalysts and chiral auxiliaries in asymmetric synthesis.^15^ Both −OH and −NH functional groups can act as hydrogen-bond donors and acceptors, and thus, amino alcohols can form O–H···N or N–H···O intramolecular hydrogen bonds. Such versatility gives rise to the existence of multiple conformers and leads to competition between intra- and intermolecular hydrogen bonding when interacting with solvents. As a result, two distinct types of configurations can appear for amino alcohol-solvent clusters: addition structures, where the solvent adds to the intramolecular hydrogen bond without interfering with it, and insertion structures, where the solvent disrupts the intramolecular hydrogen bond. Insertion structures can also induce conformational changes in the amino alcohol, potentially altering the activity of the larger molecule in which the amino alcohol is embedded. Understanding the conformational preferences of amino alcohols and how they are modulated by solvent interactions is therefore essential for accurately describing and controlling the molecular recognition and self-assembly processes in which they participate.

Among vicinal amino alcohols, prolinol (C_5_H_11_NO) stands out due to its cyclic structure, featuring a secondary amino group incorporated within a five-membered ring. (*S*)-prolinol finds extensive application as an intermediate in the synthesis of enantiomerically pure compounds such as pyrrolobenzodiazepines, a class of DNA minor groove binders,^16^ and 3-hydroxypiperidines, important components of many biologically active compounds.^17^ Prolinol is also a highly enantioselective catalyst.^18^ A key feature of prolinol in drug discovery is its flexible five-membered pyrrolidine ring, which, through ring puckering, allows access to a large three-dimensional space.^19^ The conformational landscape of prolinol was investigated in the gas phase by Fourier transform infrared (FTIR) spectroscopy,^20^ where two −OH stretching bands were tentatively assigned to the two lowest-energy conformers. While FTIR is sensitive to local vibrational modes and provides qualitative information on functional groups and chemical bonding, it does not yield precise structural details. High-resolution spectroscopy is therefore essential to accurately determine conformational geometries.

Microwave spectroscopy, with its exceptional resolution and high sensitivity to subtle structural changes, is an ideal technique for conformational investigations. Different conformers and isomers present distinct rotational spectra, typically consisting of tens to hundreds of transitions with various patterns that reflect differences in molecular mass distribution. Their analysis leads to the determination of the experimental rotational constants, *A*, *B*, and *C*, which are inversely proportional to the moments of inertia. Rotational transition intensities are dictated by the values of molecular electric dipole moment components along the principal inertial axes, μ_*a*_, μ_*b*_, and μ_*c*_. Sizable values of μ_*a*_, μ_*b*_, and μ_*c*_ give rise to *a*-, *b*-, and *c*-type rotational spectra, respectively. Additionally, if the molecular species contains a nucleus with a quadrupole moment, each rotational transition will show a hyperfine structure arising from the interaction of the nuclear electric quadrupole moment with the molecular electric field gradient. This is the case of the ^14^N (nuclear spin *I* = 1) in prolinol. The experimental parameters describing this interaction are the nuclear quadrupole coupling constants χ_gg_ (g = *a*,*b*,*c*), which inform on the electronic environment around the quadrupolar nucleus, thus providing another set of data, independent from the rotational constants, that allows conformational identification. Isotopologues in natural abundance can also be detected, provided that line intensities are sufficient, showing the same spectral patterns as the parent species but shifted to lower frequencies.

Consideration of all of the above microwave experimental data and its comparison with theoretically predicted parameters allows unambiguous conformer identification and, when isotopologues are observed, experimental determination of molecular structures. Rotational spectroscopy has been successfully applied to the investigation of chiral molecules and their complexes, including clusters with a high number of water molecules.^21–26^

In this work, we present the study of the conformational landscape of the neutral amino alcohol prolinol and its stepwise hydration using broadband Fourier transform microwave spectroscopy^27^ supported by quantum chemical calculations. This technique captures broad spectral regions, typically several GHz, facilitating the assignment of spectral patterns associated with the various species present. Moreover, it allows the accumulation of millions of spectra, significantly enhancing sensitivity and enabling the detection of low-abundance species, such as clusters with several water molecules. Experiments are conducted in a supersonic jet, which simplifies the spectrum while increasing line intensity as all species are cooled to their ground vibrational states, and enables cluster formation from collisions at the initial stages of the expansion. Clusters with different numbers of water molecules are produced. No mass selection is used; signals from all possible species contribute to the rotational spectrum with virtually no overlap due to its exceptional high resolution and can be decoded according to their rotational and quadrupole coupling constants and their dipole moment components.

We conclusively identified two conformers of bare prolinol, corresponding to the lowest-energy forms, and determined their structures through the analysis of the rotational spectra of their parent species and ^13^C, ^15^N, and ^18^O isotopologues. Complexation of prolinol with up to three water molecules reveals a complicated interplay of interactions, resulting in huge changes in prolinol’s conformational preferences. While the monohydrates retain the low-energy conformers of bare prolinol, the di- and trihydrates feature the higher-energy prolinol conformers. Overall configuration preferences of the complexes also change. Insertion structures are preferred in the monohydrates, whereas addition structures are favored in the di- and trihydrated complexes.

## Results and Discussion

### Prolinol

The rotational spectrum of (*S*)-prolinol was recorded in the 2–18 GHz frequency range using broadband microwave spectrometers at the University of Virginia^27,28^ and the University of the Basque Country.^29,30^ Prolinol is a five-membered ring, and as such, it has two out-of-plane ring motions, which give rise to envelope (E) or twisted (T) configurations. Seven conformers, including E and T types with different atoms above or below the plane of the ring, varying ∠OCCN dihedral angles and intramolecular hydrogen bonds, were predicted within 12 kJ mol^–1^ by MP2 and B3LYP-D3BJ calculations (see Table S1). The conformers, labeled **I–VII** according to their relative energies at the B3LYP-D3BJ/6–311++G­(d,p) level, can be grouped into two families based on the orientation of the hydroxymethyl group. Conformers **I**, **III**, and **VII** feature ∠OCCN dihedral angles of approximately −60°, while conformers **II**, **IV**, **V**, and **VI** have ∠OCCN angles close to +60°. The most stable conformers, **I–V**, are stabilized by O–H···N hydrogen bonds, whereas the higher-energy conformers **VI** and **VII** exhibit N–H···O interactions. All conformers are asymmetric tops near the prolate limit, with large dipole moment components along the *a* principal inertial axis. Therefore, they are expected to show characteristic patterns of *a*-type *J*+1 ← *J* transitions, separated by approximately the sum of rotational constants *B* + *C*.

Two distinct sets of transitions, showing suitable patterns and corresponding to two different conformers, were readily identified in the rotational spectrum. All transitions exhibited hyperfine splittings, indicative of the presence of a single ^14^N nucleus in each species (see Figure 1). The measured transitions were fit^31^ to the A-reduced Watson Hamiltonian^32^ in the I^*r*^ representation, supplemented by an additional term, H_Q_,^33^ accounting for the nuclear quadrupole coupling interaction. The determined values of the experimental rotational and nuclear quadrupole coupling constants (NQCCs) clearly match those predicted by theory for conformers **I** and **II** (see Table 1). The average differences between experimental and theoretical rotational constants are 0.3% (1.3%) and 0.5% (0.5%) for conformers **I** and **II**, respectively, using B3LYP-D3BJ (MP2).

**Figure 1:**
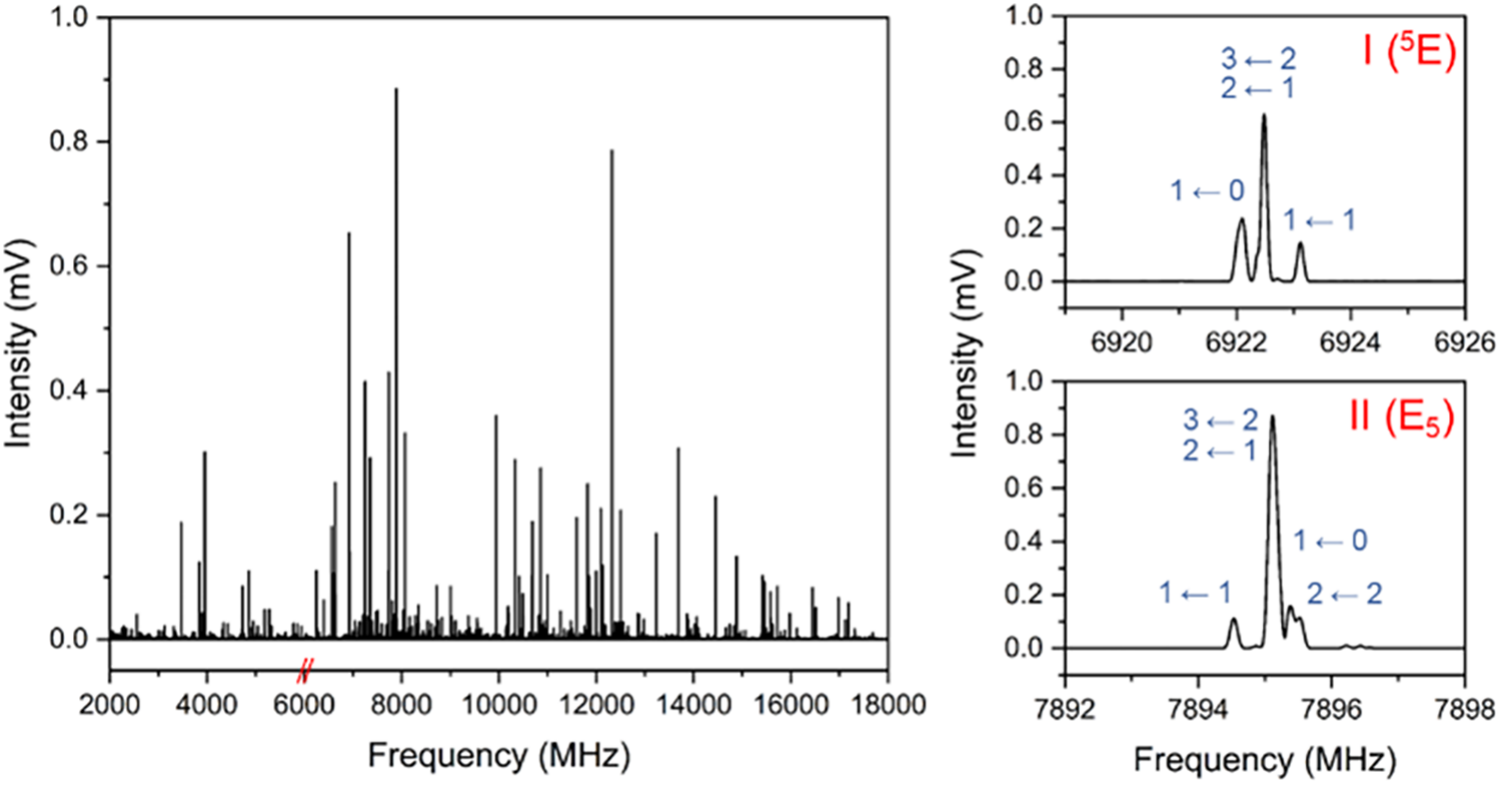
(left) Broadband rotational spectrum of prolinol in the 2–6 and 6–18 GHz frequency range. (right) Observed rotational transition 2_0,2_ ← 1_0,1_ for conformers I (E_5_) and II (^5^E) of prolinol, showing their different nuclear quadrupole coupling hyperfine structure. Hyperfine components are labeled with their quantum numbers *F*′ ← *F*″.

**Table 1:** Experimental and Theoretical Spectroscopic Parameters of the Observed Monomers of Prolinol

	I			II		
parameter	experimental	B3LYP	MP2	experimental	B3LYP	MP2
*A* ^a^ (MHz)	4507.02605(41)^d^	4489.2	4475.6	5241.6960(14)	5198.4	5271.5
*B* (MHz)	2059.27298(20)	2054.5	2089.3	1888.2816(12)	1897.0	1898.1
*C* (MHz)	1892.44146(20)	1888.1	1926.3	1582.9077(10)	1585.4	1590.7
Δ_*J*_ (kHz)	1.4677(46)	1.17	1.11	0.624(16)	0.62	0.48
Δ_*JK*_ (kHz)	–7.022(13)	–5.39	–4.81	–2.451(89)	–2.89	–1.87
Δ_*K*_ (kHz)	13.345(41)	10.40	8.88	7.75(30)	9.16	6.41
δ_*J*_ (kHz)	–0.1627(15)	–0.13	–0.12	0.0442(61)	0.04	0.04
δ_*K*_ (kHz)		–0.67	–0.62	1.11(49)	0.65	0.56
χ_*aa*_ (MHz)	1.1275(21)	1.13	1.07	–1.3383(31)	–1.61	–1.44
χ_*bb*_ (MHz)	2.1608(29)	2.47	2.24	0.1920(48)	0.25	0.20
χ_*cc*_ (MHz)	–3.2883(29)	–3.60	–3.31	1.1464(48)	1.36	1.25
|μ_*a*_|/|μ_*b*_|/|μ_*c*_|	y/y/y	2.1/0.8/1.9	2.1/0.9/1.9	y/y/y	3.3/0.3/0.5	3.3/0.3/0.5
σ^b^ (kHz)	6.9			8.3		
*N* ^c^	346			203		

a *A*, *B*, and *C* are the rotational constants; Δ_*J*_, Δ_*JK*_, Δ_K_, δ_*J*_, and δ_*K*_ are the quartic centrifugal distortion constants; χ_*aa*_, χ_*bb*_, and χ_*cc*_ are the nuclear quadrupole coupling constants*; |μ*_*a*_ *|*, *|μ*_*b*_ *|, and |μ*_*c*_ *|* are the absolute values of the electric dipole moment components in Debye; y and n, yes and no, indicate whether *a*-, *b*-, and *c*-type transitions are observed or not.

b σ is the rms deviation of the fit.

c *N* is the number of fitted hyperfine components.

d Standard error in parentheses in units of the last digit.

Additional confirmation of the assignment of conformers **I** and **II** was obtained through the observation of the rotational transitions of all their ^13^C, ^15^N, and ^18^O isotopologues in natural abundance (1.1, 0.4, and 0.2%, respectively), at their predicted frequency shifts (see Figure S1). The spectroscopic parameters of these isotopologues for both conformers are reported in Tables S2–S3 of the Supporting Information. These results allow the unambiguous identification of the observed prolinol conformers and support the initial assignment from FTIR data.^20^

No transitions attributable to other predicted conformers of prolinol were detected. This is likely due to conformational relaxation caused by collisions with the carrier gas at the onset of the supersonic expansion, a process that typically occurs when interconversion barriers are below ∼4.8 kJ mol^–1^.^34^ In our case, the absence of conformers **III** and **IV** is attributed to their relaxation to conformers **I** and **II**, respectively. These relaxations only involve changes in ring puckering and are theoretically predicted to have barriers of approximately 2.3 and 1.2 kJ mol^–1^ (see Figure S6). The remaining conformers lie more than 9 kJ mol^–1^ above the global minimum and thus are expected to be negligibly populated.

The assignment of the ^13^C, ^15^N, and ^18^O isotopologues enabled the determination of the experimental substitution (*r*_s_) and effective (*r*_0_) structures for both conformers. The *r*_s_ structure is obtained by considering the difference between the moments of inertia of the parent molecule and each isotopologue^35^ to determine the atomic coordinates. Alternatively, a least-squares fit of the moments of inertia of all observed species yields the *r*_0_ structure, which best reproduces the experimental moments of inertia. Since not all structural parameters could be determined from the fits, some of them were fixed to those predicted by B3LYP-D3BJ calculations, which showed better agreement with experiment. A comparison of the *r*_s_ and *r*_0_ structures, along with the B3LYP-D3BJ and MP2 equilibrium structures, is provided in Tables S4–S5 and Figure 2. The structural parameters are in good agreement across the different methods and are also very similar between the two conformers. Both conformers adopt an envelope configuration of the ring, where C5 is displaced out of the plane defined by the dihedral angle ∠C4C3C2N1.

**Figure 2:**
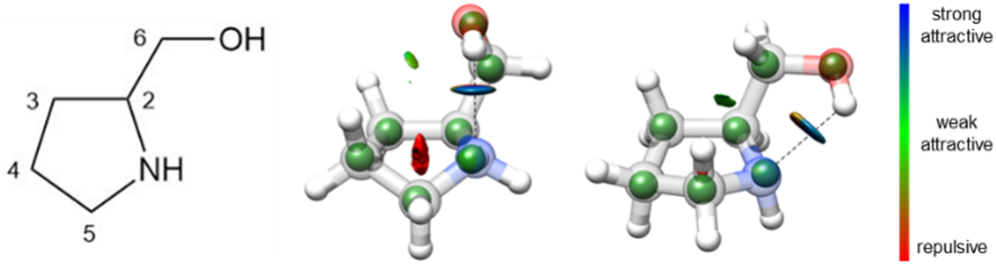
Observed I (E_5_) (left) and II (^5^E) (right) conformers of prolinol, showing the theoretical structures (gray) with the experimental *r*_s_ atom coordinates indicated as green spheres. The NCI isosurfaces (*s* = 0.5) for values of sign­(λ_2_)*ρ* from −0.025 to +0.025 au are also shown. The colors indicate interaction strength and type: blue for strong attractive interactions, green for weak attractive interactions, and red for repulsive interactions.

The main difference lies in the puckering angle of the ring, close to 26° in both cases but with opposite signs, indicating an exo arrangement of C5 with respect to the −CH_2_OH chain in conformer I and an endo arrangement in conformer II (see Figure 2). The possible ring configurations are more precisely described using Cremer–Pople polar coordinates,^36^ namely, the puckering of the ring, given by the angle Φ, and the puckering amplitude *q*, which indicates the perpendicular displacement of the atoms from a planar ring. These coordinates are represented in a circular diagram where the center of the circle corresponds to a planar ring (*q* = 0) and the values of Φ specify envelope (E) and twisted (T) configurations corresponding to different atoms being above or below the planar ring, indicated with a superscript or a subscript, respectively. Using this nomenclature, the observed conformers of prolinol are labeled as E_5_ (I) and ^5^E (II) and represented in the Cremer–Pople diagram as red circles (see Figure 3). They are on opposite sides of the diagram because both conformers are envelope forms with the same atom (C5) above or below the ring.

**Figure 3:**
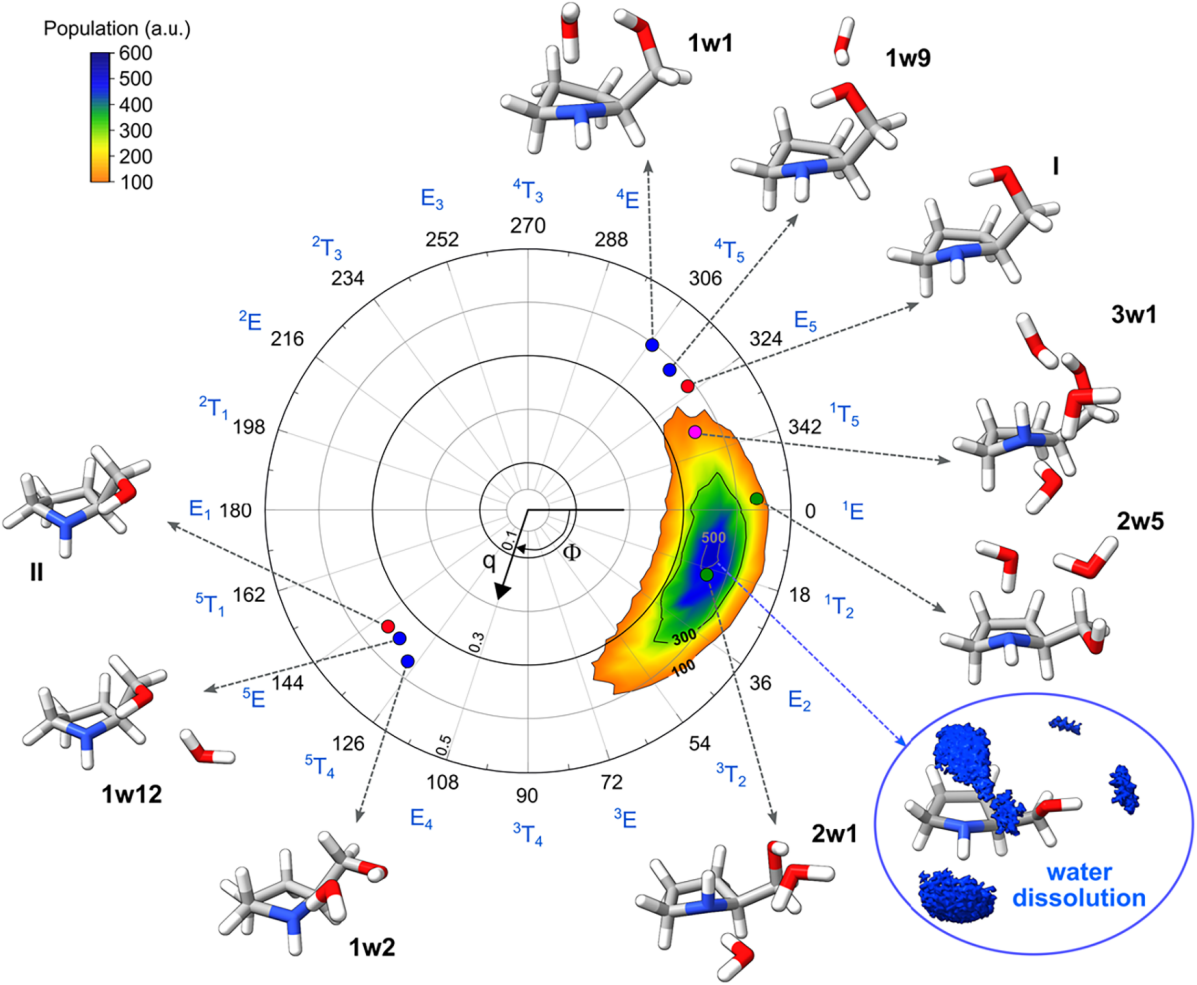
Cremer–Pople diagram illustrating the ring-puckering parameters of the observed conformers of prolinol and its hydrated complexes. The radial coordinate (q) represents the puckering amplitude in Å, and the angular coordinate (Φ) indicates the phase of the ring puckering in degrees. Red circles correspond to the monomer, blue to prolinol–H_2_O complexes, green to prolinol–(H_2_O)_2_, and pink to prolinol–(H_2_O)_3_. The colored surface represents the conformational population in aqueous solution obtained from 1 μs molecular dynamics (MD) simulations. The experimentally observed structures are shown around the diagram, while the bottom right panel displays the time-averaged solution structure obtained from MD simulations, together with the corresponding oxygen density map of the first solvation shell around prolinol.

Both conformers are stabilized by an intramolecular O–H···N hydrogen bond where the amino group acts as a hydrogen acceptor and the hydroxyl oxygen acts as a hydrogen donor. These interactions are visualized as blue regions in the noncovalent interaction (NCI) plots shown in Figure 2, which are obtained from analyzing electron density and reduced density gradients.^37,38^ Conformer **I** is further stabilized by a secondary C–H···O hydrogen bond between a lone pair on the oxygen atom and a –CH group within the ring. In contrast, conformer **II** displays only a weak dispersion-type H···H contact in addition to the primary O–H···N hydrogen bond. These weaker attractive interactions appear as green isosurfaces in Figure 2. Conformers **I** and **II** are very close in energy, and their relative abundances reflect this, with conformer **I** accounting for 59(7)% and conformer **II** accounting for 41(7)% of the population. These values were estimated from common *a*-type transitions, assuming that conformer number density *N*_*i*_ is proportional to line intensity *I*_*i*_ and inversely proportional to the square of the corresponding dipole moment component, μ_*ia*_^2^.

#### Prolinol–(H_2_O)_1–3_

After two conformers of prolinol were identified, the rotational spectrum still exhibited numerous additional lines featuring the nuclear quadrupole hyperfine structure characteristic of species containing a single ^14^N nucleus (Figure 4). We initially assigned two new species (see Table 2, columns 1 and 2), with rotational constants smaller than those of the observed prolinol conformers. Suspecting that these lines could arise from hydrated complexes of prolinol, we recorded the spectrum again after introducing water into the injection line. This confirmed our initial hypothesis, as we observed a marked increase in the spectral intensity of the newly assigned species. Further spectral searches led to the identification of two additional prolinol–H_2_O complexes, shown in columns 3 and 4 of Table 2. Comparison of the experimental rotational constants and NQCCs with theoretical predictions allows us to assign the observed species to complexes **1w1**, **1w2**, **1w9**, and **1w12**. Complexes are labeled as XwY, where X indicates the number of water molecules in the complex and Y is an index according to the relative energy ordering (including zero-point corrections) obtained from B3LYP-D3BJ predictions. The best agreement was found with the theoretical structures at the B3LYP-D3BJ/6–311++G­(d,p) level of theory (see Tables 2 and S8). Considering common transitions, the estimated experimental abundances follow the trend **1w1 ≈ 1w2 > 1w9 ≈ 1w12**.

**Figure 4:**
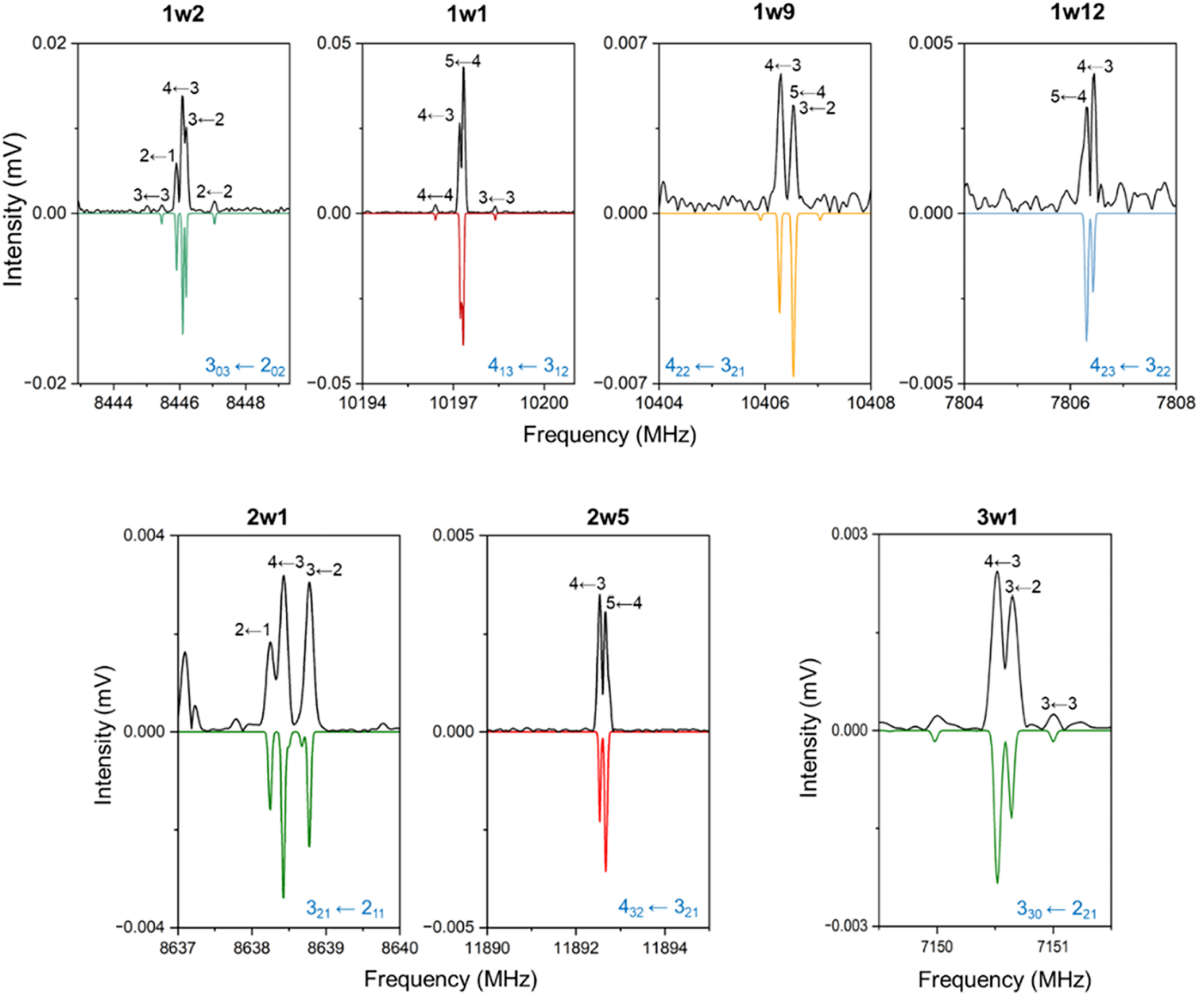
Sections of the broadband rotational spectrum of prolinol–water showing selected rotational transitions for the observed hydrated complexes. The experimental spectra are shown in black, and the corresponding fitted spectra are shown inverted in color, with each color representing a different isomer. The rotational transitions are labeled in blue as *J*′_*K′‑1, K′+1*_ ← *J*″_*K″‑1, K″+1*_, and their nuclear quadrupole coupling hyperfine components (labeled in black) are indicated with their quantum numbers *F*′ ← *F*″.

**Table 2:** Experimental and Theoretical Spectroscopic Constants for the Four Observed Isomers of the Prolinol–H_2_O Complex

	1w1			1w2		
parameter	experimental	B3LYP	MP2	experimental	B3LYP	MP2
*A* ^a^ (MHz)	2180.92974(70)^d^	2207.3	2199.6	2699.50099(40)	2740.9	2753.8
*B* (MHz)	1738.24469(71)	1736.1	1766.5	1391.44859(31)	1392.0	1399.5
*C* (MHz)	1291.50399(42)	1300.3	1319.8	1010.33763(20)	1011.8	1020.9
Δ_*J*_ (kHz)	0.661(20)	0.63	0.59	0.2855(60)	0.21	0.22
Δ_*JK*_ (kHz)	1.330(48)	3.98	1.23	0.737(26)	0.94	0.70
Δ_*K*_ (kHz)		–3.67	–0.74	0.795(44)	0.22	0.56
δ_*J*_ (kHz)	0.106(12)	0.13	0.13	0.0623(29)	0.04	0.04
δ_*K*_ (kHz)		2.05	0.85	0.813(55)	0.74	0.62
χ_*aa*_ (MHz)	–2.9665(37)	–2.86	–2.86	–3.1185(21)	–3.29	–3.13
χ_*bb*_ (MHz)	1.9435(61)	1.43	1.67	1.7233(33)	1.75	1.62
χ_*cc*_ (MHz)	1.0231(61)	1.18	1.19	1.3953(33)	1.54	1.51
|μ_*a*_|/|μ_*b*_|/|μ_*c*_|	y/y/y	2.3/0.8/3.4	2.2/1.0/3.4	y/y/y	3.8/1.8/1.3	3.8/1.9/1.1
σ^b^ (kHz)	7.3			5.7		
*N* ^c^	127			263		

	**1w9**			**1w12**		
parameter	experimental	B3LYP	MP2	experimental	B3LYP	MP2
*A* ^a^ (MHz)	2455.5219(24)^d^	2375.4	2475.2	3355.83(28)	3297.9	3382.8
*B* (MHz)	1395.79218(80)	1479.5	1427.8	1003.17663(45)	1016.0	1013.9
*C* (MHz)	1105.91727(52)	1154.5	1130.1	948.61336(46)	984.1	958.1
Δ_*J*_ (kHz)	2.900(11)	1.43	2.14	0.7692(40)	0.82	0.86
Δ_*JK*_ (kHz)	–13.092(64)	–4.76	–8.85	–3.602(97)	–4.17	–4.28
Δ_*K*_ (kHz)		7.33	14.12		17.29	20.50
δ_*J*_ (kHz)	0.9076(82)	0.42	0.64		–0.10	–0.08
δ_*K*_ (kHz)		0.01	–0.05		12.37	8.77
χ_*aa*_ (MHz)	–0.5848(55)	–0.99	–0.51	0.8725(51)	0.72	0.79
χ_*bb*_ (MHz)	2.2088(76)	2.52	2.29	–2.7839(76)	–2.99	–2.76
χ_*cc*_ (MHz)	–1.6240(76)	–1.52	–1.76	1.9113(76)	2.27	1.96
|μ_*a*_|/|μ_*b*_|/|μ_*c*_|	y/y/n	2.4/1.5/0.4	2.5/1.6/0.5	y/n/n	2.3/0.0/0.3	2.4/0.1/0.2
σ^b^ (kHz)	6.2			6.4		
*N* ^c^	89			79		

a *A*, *B*, and *C* are the rotational constants; Δ_*J*_, Δ_*JK*_, Δ_K_, δ_*J*_, and δ_*K*_ are the quartic centrifugal distortion constants; χ_*aa*_, χ_*bb*_, and χ_*cc*_ are the nuclear quadrupole coupling constants; |μ_*a*_|, |μ_*b*_|, *and* |μ_*c*_| are the absolute values of the electric dipole moment components in Debye; y and n, yes and no, indicate whether *a*-, *b*-, and *c*-type transitions are observed or not.

b σ is the rms deviation of the fit.

c *N* is the number of fitted hyperfine components.

d Standard error in parentheses in units of the last digit.

Several prolinol–H_2_O complexes are predicted to lie at energies lower than those of **1w12** (Table S8), the highest-energy isomer observed experimentally, but no signals attributable to these species were detected. Most of them present conformational relaxation barriers to **1w1** or **1w2** below 4.8 kJ mol^–1^ (Figures S7–S8), thus rationalizing their absence from the rotational spectrum. We detect the lowest-energy isomers **1w1** and **1w2**, which are thermodynamically favored, as well as the higher-energy complexes, **1w9** and **1w12**, where water adds to the experimentally observed conformers of prolinol and which are likely kinetically trapped in the supersonic expansion.

Upon removing the rotational transitions arising from prolinol and its monohydrated complexes, a substantial number of lines remained unassigned. Careful analysis of the spectrum led to the identification of two complexes of prolinol with two water molecules, **2w1** and **2w5**, and one complex with three water molecules, **3w1** (Table 3). They were identified based on the agreement between theoretical and experimental rotational constants and NQCCs. In the case of **2w1**, two other prolinol–(H_2_O)_2_ isomers, **2w3** and **2w6**, show similar predicted rotational and quadrupole parameters. Both are predicted to lie at slightly higher energies by the B3LYP-D3BJ and MP2 methods (see Table S9), and differ from **2w1** only in the orientation of the dangling water hydrogens. They present low barriers for relaxation by collisions in the supersonic expansion (almost inexistent in the case of **2w6**, see Figure S9), which explains their nonobservation and supports our assignment of the observed species to **2w1**. As previously observed for the isomers of the monohydrate, B3LYP-D3BJ predicts rotational constants for prolinol–(H_2_O)_2,3_ with better overall agreement with the experimental ones than MP2.

**Table 3:** Experimental and Theoretical Spectroscopic Constants for the Observed Isomers of the Prolinol–(H_2_O)_2,3_ Complexes

	2w1			2w5			3w1		
par.	exp.	B3LYP	MP2	exp.	B3LYP	MP2	exp.	B3LYP	MP2
*A* ^a^	1904.38772(84)^d^	1938.8	1950.9	1889.52731(37)	1914.9	1950.5	1299.84843(46)	1302.5	1317.0
*B*	926.91092(39)	932.3	929.8	928.82164(25)	937.7	923.5	700.50066(29)	710.3	708.7
*C*	719.28840(34)	723.4	723.4	742.61818(25)	750.4	750.4	580.21468(36)	596.0	596.9
Δ_*J*_	0.3152(47)	0.22	0.30	0.4272(27)	0.26	0.29	0.1812(43)	0.10	0.13
Δ_*JK*_	–1.377(31)	–0.90	–1.34	–1.640(14)	–0.86	–1.07	0.123(19)	0.91	0.81
Δ_*K*_	4.718(30)	3.18	4.35	4.331(23)	2.64	0.32	0.465(22)	–0.33	0.22
δ_*J*_	–0.0475(25)	0.04	0.06	–0.1174(14)	0.07	0.08		0.03	0.04
δ_*K*_		0.34	0.39	–0.242(45)	0.23	0.25		0.14	0.38
χ_*aa*_	–0.6419(65)	–0.69	–0.74	0.8285(31)	0.88	0.84	–1.1241(54)	–1.12	–1.13
χ_*bb*_	–0.2173(95)	–0.23	–0.25	–0.6661(56)	–0.66	–0.89	–0.4464(81)	–0.01	–0.41
χ_*cc*_	0.8592(95)	0.92	0.99	–0.1625(56)	–0.22	0.06	1.5704(81)	1.13	1.55
|μ_*a*_|	y	1.0	0.9	y	2.7	2.5	n	0.1	0.3
|μ_*b*_|	y	2.2	2.2	y	2.1	2.1	y	2.1	1.9
|μ_*c*_|	y	0.8	0.9	y	0.6	0.7	y	0.6	0.5
σ^b^	8.4			6.3			8.6		
*N* ^c^	130			211			124		

a *A*, *B*, and *C* are the rotational constants; Δ_*J*_, Δ_*JK*_, Δ_K_, δ_*J*_, and δ_*K*_ are the quartic centrifugal distortion constants; χ_*aa*_, χ_*bb*_, and χ_*cc*_ are the nuclear quadrupole coupling constants; |μ_*a*_|, |μ_*b*_|, *and* |μ_*c*_| are the absolute values of the electric dipole moment components in Debye; y and n, yes and no, indicate whether *a*-, *b*-, and *c*-type transitions are observed or not.

b σ is the rms deviation of the fit.

c *N* is the number of fitted hyperfine components.

d Standard error in parentheses in units of the last digit.

### Stepwise Hydration and Conformational Preferences

The isomers observed for prolinol–H_2_O correspond to water binding to conformers **I** and **II** of bare prolinol, forming insertion or addition structures depending on whether the water molecule disrupts the intramolecular hydrogen bond or simply adds to it. In insertion structures, the water molecule interacts with both amino and hydroxyl groups via O_w_–H_w_···N and O–H···O_w_ hydrogen bonds, simultaneously acting as a hydrogen donor and acceptor (see Figure 5). In contrast, in addition structures, the water molecule only binds to the hydroxyl group through an O_w_–H_w_···O hydrogen bond, acting solely as a donor. The addition structures also show C–H···O_w_ hydrogen bonds. A cooperative chain of hydrogen bonds, O–H···O–H···N, is established in both types of complexes.

**Figure 5:**
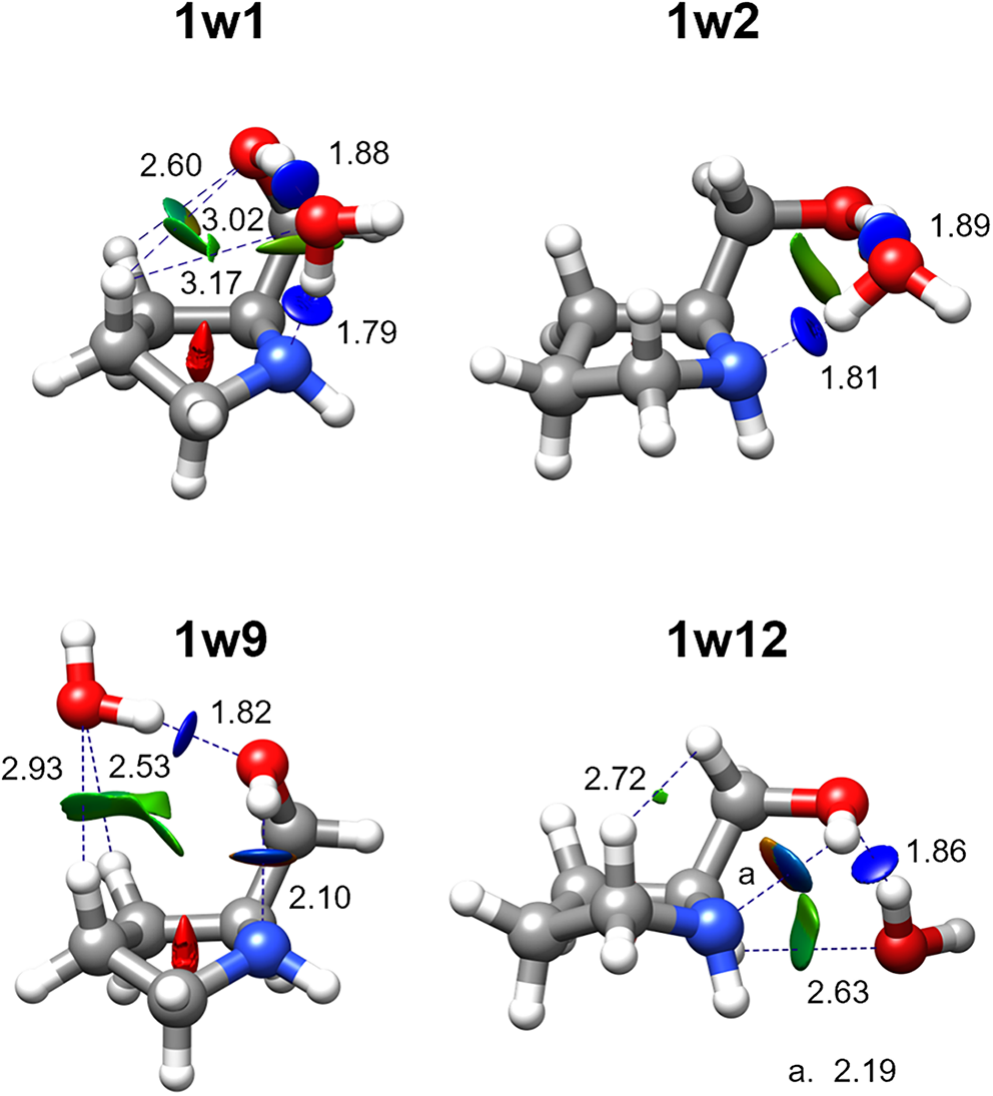
Theoretical structures of the four observed isomers of prolinol–H_2_O (**1w1**, **1w2**, **1w9**, **1w12**), optimized at the B3LYP-D3BJ/6–311++G­(d,p) level of theory. Intermolecular interactions are indicated with blue dotted lines along with associated theoretical distances (in Å). NCI isosurfaces (*s* = 0.5) are shown for values of sign­(*λ*_2_)*ρ* ranging from −0.025 to +0.025 au. The colors indicate interaction strength and type: blue for strong attractive interactions, green for weak attractive interactions, and red for repulsive interactions.

However, the O–H···N bonds are much longer (by 0.3–0.4 Å) in the addition structures due to their intramolecular nature and the constraints imposed by prolinol geometry. This explains why insertion structures are more abundant and predicted to be energetically favored (Table S8). NBO analysis^39^ (Tables S11–S14) confirms that intermolecular hydrogen-bonding interactions in insertion structures are substantially more stabilizing than those in addition structures, consistent with experimental observations.

In prolinol–(H_2_O)_2,3_, the water molecules form a chain of hydrogen bonds bridging the hydroxyl and amino groups, acting as hydrogen acceptors to the hydroxyl group and donors to the amino group (see Figure 6). These interactions do not disrupt the intramolecular hydrogen bond, and thus, prolinol–(H_2_O)_2,3_ solely exhibit addition structures, in contrast to the monohydrated species. The most radical change, however, concerns the conformation of prolinol, which no longer corresponds to the low-energy conformers **I** and **II**, observed in the monomer and monohydrates. In the di- and trihydrated complexes, prolinol adopts higher-energy conformers **VI** and **VII**, which lie *ca*. 9.6–10.8 kJ mol^–1^ above the global minimum (Table S1). This switch in conformational preferences also involves a modification of the intramolecular hydrogen bond, which shifts from O–H···N to N–H···O. The intramolecular N–H···O bond participates in the hydrogen-bonding network of the complexes, but it is weaker than the intermolecular hydrogen bonds established with the water molecules (see NCI plots in Figure 6).

**Figure 6:**
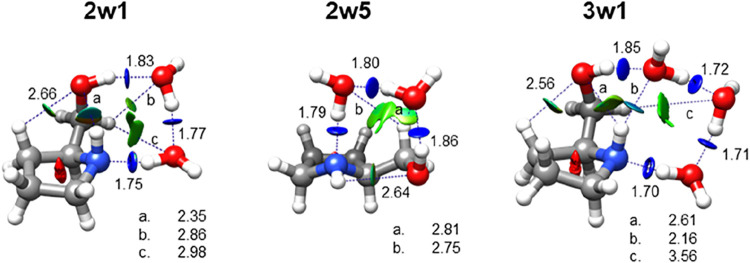
Theoretical structures of the observed isomers of prolinol–(H_2_O)_2,3_, optimized at the B3LYP-D3BJ/6–311++G­(d,p) level of theory. Intermolecular hydrogen bonds are shown as blue dotted lines along with associated theoretical distances (in Å). NCI isosurfaces (*s* = 0.5) are displayed for values of sign­(*λ*_2_)*ρ* ranging from −0.025 to 0.025 au. The colors indicate interaction strength and type: blue for strong attractive interactions, green for weak attractive interactions, and red for repulsive interactions.

In the dihydrates, the cyclic hydrogen-bonding network resembles the structure of the lowest-energy isomer of the water tetramer *S*_4_,^40^ with the −OH and −NH groups replacing one water molecule each. Similarly to *S*_4_, the oxygen and nitrogen atoms lie mostly on the same plane, and the hydrogen atoms not involved in hydrogen bonds adopt an alternating up–down configuration. In trihydrate **3w1**, a configuration akin to the water tetramer *S*_4_ is evident, involving the three water molecules and the amino group. The −OH group of prolinol participates in the hydrogen-bond network as a hydrogen donor to one water molecule and as a hydrogen acceptor via the intramolecular N–H··O bond. Both the −OH and −NH groups act as hydrogen donors to the same water molecule, with the N–H··O_w_ bond being longer and weaker than the O–H··O_w_ bond. According to B3LYP-D3BJ calculations, the increased flexibility provided by the additional water molecule in **3w1** results in an almost planar arrangement, with a dihedral angle of ∠NO_w_O_w_O_w_ = −3.2°, in contrast to ∠NOO_w_O_w_ = −16.4° in **2w5** and ∠NOO_w_O_w_ = 22.5° in **2w1**. Besides, the intramolecular N–H··O bond elongates from 2.32 Å in prolinol **VII** to 2.61 Å in the trihydrate.

In all observed hydrates, the hydrogen-bond network displays a homodromic configuration with alternating hydrogen donors and acceptors, forming a closed chain in the dihydrates and trihydrates. This arrangement boosts polarization and results in cooperativity.^41^ Upon stepwise hydration, the hydrogen bonds become shorter (see Figure 6) and their interaction energies increase, as shown by NBO calculations^39^ (Tables S11–S14). Notably, the strength of the hydrogen-bond network in prolinol–(H_2_O)_2,3_ compensates for the energetic cost of adopting higher-energy prolinol conformers, which lie approximately 10 kJ mol^–1^ above the global minimum.

The stepwise addition of water molecules to prolinol not only drastically alters its conformational preferences but also induces subtler structural changes involving ring puckering. Adding one water molecule does not modify the favored prolinol conformations, but changes the puckering of **I** and **II**, from E_5_ to ^4^T_5_ and from ^5^E to ^5^T_4_, respectively (see Figure 3). This puckering shift occurs for both insertion and addition structures of prolinol–H_2_O. Adjustments in ring puckering are also observed for conformation **VII**, which evolves from ^1^T_5_ in the monomer to ^1^T_2_ in **2w1** and E_5_ in **3w1**. In contrast, in dihydrate **2w5**, where prolinol adopts conformation **VI**, ring-puckering (^1^E) remains unchanged with respect to the bare molecule. Additional structural modifications involve the ∠OCCN dihedral angle. In the monohydrate, ∠OCCN shifts from −53.5° in conformer **I** to −72.3° in **1w1** and −47.4° in **1w9**, and from 55.1° in conformer **II** to 52.4° in **1w12** and 69.3° in **1w2**. As expected, larger changes occur in the insertion structures due to the need to accommodate the water molecule between the hydroxyl and amino groups. In dihydrate **2w5**, the ∠OCCN angle changes slightly from 66.2° in **VI** to 60.2°, whereas in di- and trihydrates involving prolinol **VII**, it shifts from −62.0° in **VII** to −52.6° in **2w1** and −68.1° in **3w1.**

Depending on the degree of hydration of prolinol, insertion or addition structures are preferred in the complexes. In the monohydrates, insertion structures are favored over addition ones, while for the dihydrates and trihydrates addition structures are energetically preferred. In agreement with experimental observations, addition structures are predicted to lie more than 5 kJ mol^–1^ higher in energy for monohydrated prolinol, and to have significantly lower binding energies according to SAPT calculations^42,43^ (see Table S15). A similar preference for insertion over addition structures is also observed in the complexes of 2-aminoethanol with a single water molecule.^44,45^ For 3-aminopropanol–H_2_O, an insertion structure is predicted to be the global minimum, but the observed complex shows an addition configuration.^46^ In the case of 4-aminobutanol-H_2_O, the experimentally observed isomer and all predicted species within 9.6 kJ mol^–1^ display addition structures.^47^

The change from insertion to addition structures with stepwise solvation suggests that water self-aggregation is the likely energetic driving force and it is reinforced by interactions with the prolinol amino and hydroxyl groups, which enhance cooperativity. The arrangement of the water molecules, resulting in a hydrogen-bond network resembling the lowest-energy isomer of the pure water tetramer, supports this interpretation. Optimizing all interactions in the hydrogen-bond network, which becomes increasingly energetic with microsolvation (see NBO predicted energies, Tables S11–S14), is also likely to be the prime agent controlling prolinol conformation. The ability of prolinol and its derivatives to adapt their ring configuration can be a determining factor in molecular recognition. For example, activation of the Put3p protein requires an unmodified pyrrolidine ring; it can be achieved with proline and prolinol but not with derivatives containing double bonds in their pyrrolidine rings.^48^

The characterization of prolinol–(H_2_O)_1–3_ reveals how stepwise hydration reshapes both the structure of the chiral solute prolinol and the configuration of the water network. The functional groups of prolinol guide the arrangement of water molecules, which collectively attempt to mimic the planar ring structure of the lowest-energy water tetramer^40^ in the di- and trihydrated complexes. Prolinol distorts the pure tetramer configuration to some extent, but it is the water network that ultimately drives prolinol to adopt higher-energy conformers. Remarkably, this transition involves a complete reorganization of the pyrrolidine ring. While structural rearrangements upon hydration had been previously observed in flexible noncyclic molecules,^45,49^ this is the first experimental report of such a dramatic conformational shift involving a pyrrolidine ring within a chiral molecule.

Next, we examined the conformational preferences of prolinol in water and methanol, with the latter chosen as a representative polar organic solvent. In water, experimental conditions were set to pH 10 to favor the neutral species. The 2D NOESY spectra recorded at 25 °C in both solvents were highly similar, showing the key cross-peaks depicted in Figures S10 and S11. Analysis of the ^3^J homonuclear coupling constants, particularly those involving the CH_2_–OH moiety, reveals that the hydroxymethyl group exhibits a high degree of conformational flexibility in solution.^50^ This finding alone strongly challenges the existence of long-lived intramolecular polar contacts involving the nitrogen atom. A similar conclusion applies to the pyrrolidine ring, whose behavior appears largely independent of the solvent conditions. In line with these experimental observations, the dominant conformational ensembles derived from 1 μs molecular dynamics (MD) simulations in explicit water and methanol are basically identical (see Figures 3, S12, and S13). Interproton distances derived from these trajectories mostly agree with the experimental NMR-based upper bounds (see Tables S39 and S40). Intriguingly, deviations were observed for a couple of these bounds, likely representing minor populations around the ^5^E region of the pseudorotational path not adequately accounted for by the trajectories. Apart from these minor contributors, the most relevant conformers correspond to ^1^T_2_ forms spanning the E_2_–^1^E region (see Figure 3). Furthermore, our analysis of the first hydration shell reveals a shared water density between the hydroxyl and amino groups, which resembles the discrete water molecules found to be complexed between these two moieties in the gas phase. Indeed, related ring-puckering patterns are also observed in the gas phase when the molecule is complexed with two or three water molecules. Crucially, no intramolecular hydrogen bonding was detected between the hydroxyl and amino groups in either aqueous or methanolic solution. This observation provides a rationale for the distinct conformational behavior seen in condensed phases compared to the gas phase. As a result, in the solvents examined here (water and methanol), the effective chiral environment of prolinol remains dynamic, owing to the flexibility of the hydroxymethyl group. This is consistent with the common use of additional substituents in prolinol-based organocatalysts to enforce conformational organization when high asymmetric induction is desired.^51,52^

Conformational changes induced by interactions with water may be relevant for other molecular systems containing flexible rings. A biologically significant example is the amino acid proline, which is well-known for favoring β-turns due to the constraints imposed by its pyrrolidine ring.^53^ Four conformers of proline have been experimentally observed, featuring E_5_ and ^5^E puckering configurations as well as O–H···N and N–H···O intramolecular hydrogen bonds.^54,55^ Computational studies predict that interactions with just one water molecule change proline’s conformational preferences from the global minimum with an O–H···N intramolecular bond to a higher-energy conformer (lying ∼ 9 kJ mol^–1^ above) featuring an N–H···O bond.^56^ This shift closely parallels the behavior observed in prolinol. While proline-water complexes have not been detected experimentally yet, theoretical predictions suggest that higher-energy proline conformers must be considered to describe its microsolvation, highlighting the strong structural impact of even minimal hydration.

## Conclusions

The conformational landscape of prolinol and its stepwise hydration were elucidated by a combination of rotational spectroscopy and quantum-chemistry calculations. The high resolution of rotational spectroscopy has been instrumental in unambiguously identifying the observed species through their rotational and nuclear quadrupole coupling constants. The microsolvation of prolinol reveals the profound changes that water exerts on the solute structure. Whereas upon binding a single water molecule, prolinol retains its native conformation, further hydration drives a complete rearrangement, altering both the puckering of the ring and the intramolecular hydrogen bond to optimize interactions with the solvent. Water thus acts as a conformational switch for prolinol, overriding its inherent conformational preferences.

Our results unveil the specific orientations adopted by water molecules in their interactions with prolinol and underscore the relevance of even a few solvent molecules in overall structural arrangements. A proper understanding of the interactions with the solvent is essential to determining the three-dimensional configuration of prolinol. This raises a crucial question: is molecular recognition governed solely by the bare solute, or do solute–solvent complexes play a decisive role? The data reported here provide a first insight into the behavior of prolinol in water, with potential implications for its role in chemical reactions and catalysis. Furthermore, the structures of the prolinol–water complexes identified here may constitute the nucleation core of a chiral solvation shell around prolinol. In addition, complementary NMR spectroscopy and explicit-solvent molecular dynamics simulations in bulk water and methanol predict that, in contrast to the compact, hydrogen-bond-stabilized conformations observed in the gas phase and in small, hydrated clusters, prolinol exhibits a significant flexibility, adopting more open conformational ensembles in solution, with no evidence of a persistent intramolecular hydrogen bond. This indicates that extensive solvation by polar environments promotes dynamic conformational averaging, which highlights the key role of collective solvent interactions in shaping the structure of prolinol. Our combined gas-phase and solution-phase analysis thus establishes a coherent picture in which water not only solvates prolinol but actively dictates its accessible conformational space, a principle that should be broadly relevant to amino alcohol chemistry in aqueous environments.

## Supplementary Material



## Notes

Details on the computational and experimental methods, tables of spectroscopic constants, NBO and SAPT calculations, NCI and RDG plots, lists of measured transitions, and analysis in solution; Also additional references within the Supporting Information;^57–72^ Data available in AMSActa: https://amsacta.unibo.it/id/eprint/8627/ (PDF)

## #

Deutsches Elektronen-Synchrotron (DESY), Notkestr. 85, 22607 Hamburg, Germany

## ∇

Grupo de Espectroscopía Molecular (GEM), Edificio Quifima, Area de Química-Física, Laboratorios de Espectroscopía y Bioespectroscopía, Parque Científico UVa, Unidad Asociada CSIC, Universidad de Valladolid, 47011 Valladolid, Spain

## ○

Departamento de Química Física y Química Inorgánica, Facultad de Ciencias – I.U. CINQUIMA, Universidad de Valladolid, Paseo Belén 7, 47011 Valladolid, Spain

## ◆

Department of Chemistry “G. Ciamician”, University of Bologna, Via Gobetti 85, Bologna, Italy
