# Supplementary material for: Stepwise Hydration Reveals Conformational Switching in Chiral Prolinol
Source: J Am Chem Soc. 2025 Dec 11;147(51):47126–37. doi: 10.1021/jacs.5c13582 (PMC12750993; doi:10.1021/jacs.5c13582)

Stepwise hydration reveals conformational switching in chiral prolinol

Donatella Loru,[a],† Elena R. Alonso,[b],‡ Aran Insausti,[b] Cristóbal Pérez,[c],¶ Luca Evangelisti, [c],§ Juan L. Asensio,[d] Francisco Corzana,[e] Brooks H. Pate,[c] Emilio J. Cocinero,*[b], M. Eugenia Sanz*[a]

[a] Department of Chemistry, King's College London, London SE1 1DB, UK

[b] Departamento de Química Física, Universidad del País Vasco (EHU), Campus de Leioa, Ap. 644, 48080 Bilbao, Spain, and Instituto Biofisika (CSIC/EHU), 48080 Bilbao, Spain

[c] Department of Chemistry, University of Virginia, Charlottesville, Virginia 22904-4319, United States

[d] Instituto de Química Orgánica General (IQOG-CSIC), Madrid, 28006, Spain

[e] Departamento de Química and Instituto de Investigación en Química (IQUR), Universidad de La Rioja, Logroño, 26006, Spain

† Current address: Deutsches Elektronen-Synchrotron (DESY), Notkestr. 85, 22607 Hamburg, Germany

‡ Current address: Grupo de Espectroscopía Molecular (GEM), Edificio Quifima, Area de Química-Física, Laboratorios de Espectroscopia y Bioespectroscopia, Parque Científico UVa, Unidad Asociada CSIC, Universidad de Valladolid, 47011 Valladolid, Spain

¶ Current address: Departamento de Química Física y Química Inorgánica, Facultad de Ciencias – I.U. CINQUIMA, Universidad de Valladolid, Paseo Belén 7, 47011 Valladolid, Spain

§ Current address: Department of Chemistry "G. Ciamician", University of Bologna, Via Gobetti 85, Bologna, Italy

* Emilio J. Cocinero, M. Eugenia Sanz

Emails: maria.sanz@kcl.ac.uk, emiliojose.cocinero@ehu.eus

Supplementary Information

Table of Contents

1. Methods 3
2. Broadband rotational spectrum 6
3. Spectroscopic parameters 8
3.1. Prolinol 8
3.2. Prolinol-H_2O 14
3.3. Prolinol-$(H_2O)_2$ 16
3.4. Prolinol-$(H_2O)_3$ 18
4. Interconversion barriers 20
4.1. Prolinol 20
4.2. Prolinol-H_2O 21
4.3. Prolinol-$(H_2O)_2$ 23
5. NBO calculations 24
5.1. Prolinol-H_2O 24
5.2. Prolinol-$(H_2O)_2$ 26
5.3. Prolinol-$(H_2O)_3$ 27
6. SAPT calculations 28
7. NCI and RDG plots 29
8. Frequencies of measured transitions and their residuals 30
8.1. Prolinol 30
8.2. Prolinol-H_2O 53
8.3. Prolinol-$(H_2O)_2$ 63
8.4. Prolinol-$(H_2O)_3$ 69
9. Conformational analysis of (S)-prolinol in solution 72

1. Methods

1.1. Experimental

1.1.1. Rotational spectroscopy in the gas phase

The broadband rotational spectrum of (*S*)-prolinol was recorded at the University of Virginia[1,2] and at the University of the Basque Country[3,4] in the 2-18 GHz frequency range using chirped pulse Fourier transform microwave (CP-FTMW) spectrometers. Both instruments operate in a cyclic manner and have been described previously[1–4]. (*S*)-prolinol (98%, Alfa Aesar) was heated to *ca*. 373 K in bespoke heating nozzles, seeded in neon at backing pressures of 2-3 bar and supersonically expanded into a vacuum chamber. The isolated and cold molecules were then polarised by short chirp microwave pulses varying linearly in frequency from 2-8 GHz or 6-18 GHz. After polarization, the molecular free induction decay (FID) signals were collected in the time domain using a fast oscilloscope. The spectrum in the frequency domain was obtained after applying a fast Fourier transform algorithm using a Kaiser-Bessel window. The final spectra comprised ~500k FIDs (Virginia) and ~4.5 MFIDs (Basque Country).

For the broadband rotational spectra of prolinol-water complexes, an external reservoir containing water was introduced into the injection line. Hydrated complexes of prolinol were formed by streamlining the neon carrier gas (2-3 bar) over the water reservoir and then over the internal prolinol reservoir. Final spectra were obtained by averaging ~2M FIDs (Virginia) and ~700k FIDs (Basque Country).

1.1.2. NMR analysis of prolinol in solution.

NMR spectra of prolinol were recorded on a Bruker Avance 600 MHz spectrometer equipped with cryo-probe in D_2O (pH 10) or CD_3OD at 25 °C. Proton assignments were obtained through standard 2D- homonuclear NMR experiments acquired with 20 mM samples. Thus, TOCSY, NOESY and ROESY experiments were performed in the phase-sensitive mode using the TPPI method for quadrature detection in F1. Typically, a data matrix of 512 × 2K points was used to digitize a spectral width of 6000 Hz. Before Fourier transformation, zero filling was used in F1 to expand the data to 2K × 2K followed by automated baseline- and phase correction. 2D-TOCSY experiments were acquired with 16 scans per increment, 70 ms mixing time and a relaxation delay of 1.0 s. NOESY experiments were acquired employing twenty-four scans per increment, a relaxation delay of 1 s and mixing times of 400, 600 and 800 ms. Similarly, ROESY experiments were acquired employing twenty-four scans per increment, a relaxation delay of 1 s, and mixing times of 600 ms. All spectra were processed with Topspin 4.2 (Bruker Biospin) and Mestrenova programs. Homonuclear coupling constants from second order spin systems were derived by computer simulation and iterative fitting, employing routines integrated in the program Mestrenova. See Figs. S10 and S11, and Tables S39-S40.

1.2. Theoretical

1.2.1. Potential energy surfaces and geometry optimization

Initial exploration of the potential energy surface of prolinol was performed using a Monte Carlo search based on atomic redistribution and energetic optimization with MMFFs force field in the Maestro software.[5] The resulting geometries were optimised at the MP2 and B3LYP-D3BJ levels of theory using Pople's 6-311++G(d,p) basis set, within the Gaussian09 and Gaussian16 packages.[6,7] The ultrafine integration grid was used for all B3LYP calculations. Seven conformers were predicted with energies below 12 kJ mol^{-1}. Their spectroscopic parameters, including rotational constants, NQCCs, dipole moment components, and relative energies are listed in Table S1. The ring-puckering of the five-membered ring is described according to the Cremer-Pople coordinates.[8] The conformers include those reported in ref. [9], plus a new low-energy conformer, **III** ($^3T_4$). The experimental substitution structures (r_s) were obtained using the programs KRA and EVAL,[10] including Costain's error[11] in the error of the coordinates. Effective r_0 structures were calculated using the program STRFIT.[10]

For the monohydrated complexes, water molecules were first manually positioned near the various prolinol conformers considering both insertion geometries, where water disrupts the intramolecular hydrogen bond binding to both –NH and –OH functional groups, and addition geometries, where the water only forms a primary hydrogen bond with the –OH of prolinol. Additional conformational searches were performed using CREST[12] and MMFFs within Macromodel.[5,13–15] Searches with CREST used the GFN2-xTB[16] method with an energy window of 25 kJ mol^{-1}. Searches within Macromodel evaluated possible molecular configurations using the Monte Carlo method based on atomic redistribution and energetic optimization through molecular mechanics, implemented in the Maestro software,[5] concretely using fast molecular mechanics methods (MMFFs) and a hybrid conformational

search protocol that combines "Large-scales Low-Mode" exploration of neighboring minima with Monte Carlo-based torsional sampling with an energy window of 25 kJ mol^{-1}.

All resulting structures were optimised at the same level of theory as the monomer. Low-energy structures (within 12 kJ mol^{-1}) are shown in Fig. S2, and their spectroscopic parameters are compiled in Table S8. All the complexes are labelled as XwY where X indicates the number of water molecules in the complex and Y is an index according to the relative energy ordering (including zero-point corrections) obtained from B3LYP-D3BJ predictions.

For the di- and trihydrated complexes, conformational searches were conducted using CREST[12] and Macromodel[5,13–15], followed by geometry optimisation at the MP2 and B3LYP-D3BJ levels of theory with the 6-311++G(d,p) basis set. Predicted low-energy structures and their spectroscopic parameters are shown in Figs. S3-S4 and Tables S9-S10. Harmonic frequency calculations were performed for all low-energy predicted structures to verify that they were true minima.

To map the location and strength of the intra- and intermolecular interactions in the observed species, non-covalent interaction (NCI) isosurfaces were calculated using Multiwfn[17], and they are shown in Figs. 2, 5 and 6 using s = 0.5 and values of −0.025 < r*sign(l$_2$) < +0.025. Additionally, the different binding contributions in each of the observed prolinol-water clusters were obtained by performing symmetry adapted perturbation theory (SAPT) calculations[18,19] with PSI4[20] at the SAPT2+/aug-cc-pVDZ level of theory. To gain more insight into the intermolecular stabilising energy contributions, a natural bond orbital (NBO) analysis was also carried out for the hydrated complexes at the B3LYP-D3BJ/6-311++G(d,p) level of theory.

1.2.2. Molecular dynamics (MD) simulations

The simulations were carried out with AMBER 20 package[21] implemented with GAFF2 force field[22]. The parameters and charges for the prolinol were generated with the antechamber module of AMBER, using GAFF2 force field and AM1-BCC method[23] for charges. The structure found in the gas phase was used as initial structure in the calculations. Each molecule was then immersed in a water box with a 15 Å buffer of TIP3P water molecules[24] or Methanol. A two-stage geometry optimization approach was performed. The first stage minimizes only the positions of solvent molecules, and the second stage is an unrestrained minimization of all the atoms in the simulation box. The systems were then gently heated by incrementing the temperature from 0 to 300 K under the constant pressure of 1 atm and periodic boundary conditions. Harmonic restraints of 30 kcal·mol^{-1} were applied to the solute, and the Andersen temperature-coupling scheme was used to control and equalize the temperature. The time step was kept at 1 fs during the heating stages, allowing potential inhomogeneities to self-adjust. Long-range electrostatic effects were modeled using the particle-mesh-Ewald method[25]. An 8 Å cut-off was applied to Lennard-Jones interactions. Each system was equilibrated for 2 ns with a 2-fs time step at a constant volume and temperature of 300 K. Production trajectories were then run for additional 1.0 μs under the same simulation conditions. For each solvent, four independent MD simulations of 1 μs were performed, and all trajectories converged to comparable conformational distributions (see Fig. 3 in main text, Figs. S12-S13 and Tables S39-S40).

References

[1] G. G. Brown, B. C. Dian, K. O. Douglass, S. M. Geyer, S. T. Shipman, B. H. Pate, *Rev. Sci. Instrum.* **2008**, *79*, 1–13.

[2] J. L. Neill, S. T. Shipman, L. Alvarez-Valtierra, A. Lesarri, Z. Kisiel, B. H. Pate, *J. Mol. Spectrosc.* **2011**, *269*, 21–29.

[3] I. Uriarte, C. Pérez, E. Caballero-Mancebo, F. J. Basterretxea, A. Lesarri, J. A. Fernández, E. J. Cocinero, *Chem. – A Eur. J.* **2017**, *23*, 7238–7244.

[4] I. Uriarte, P. Écija, L. Spada, E. Zabalza, A. Lesarri, F. J. Basterretxea, J. A. Fernández, W. Caminati, E. J. Cocinero, *Phys. Chem. Chem. Phys.* **2016**, *18*, 3966–3974.

[5] N. Maestro, Schrödinger Release 2017-1, Schrödinger, LLC, New York, **2017**.

[6] M. J. Frisch, G. W. Trucks, H. B. Schlegel, G. E. Scuseria, M. A. Robb, J. R. Cheeseman, G. Scalmani, V. Barone, B. Mennucci, G. A. Petersson, H. Nakatsuji, M. Caricato, X. Li, H. P. Hratchian, A. F. Izmaylov, J. Bloino, G. Zheng, J. L. Sonnenberg, M. Hada, M. Ehara, K. Toyota, R. Fukuda, J. Hasegawa, M. Ishida, T. Nakajima, Y. Honda, O. Kitao, H. Nakai, T. Vreven, J. Montgomery, J. A., J. E. Peralta, F. Ogliaro, M. Bearpark, J. J. Heyd, E. Brothers, K. N. Kudin, V. N. Staroverov, T. Keith, R. Kobayashi, J. Normand, K. Raghavachari, A. Rendell, J. C. Burant, S. S. Iyengar, J. Tomasi, M. Cossi, N. Rega, J. M. Millam, M. Klene, J. E. Knox, J. B. Cross, V. Bakken, C. Adamo, J. Jaramillo, R. Gomperts, R. E. Stratmann, O. Yazyev, A. J. Austin, R. Cammi, C. Pomelli, J. W. Ochterski, R. L. Martin, K. Morokuma, V. G. Zakrzewski, G. A. Voth, P. Salvador, J. J. Dannenberg, S. Dapprich, A. D. Daniels, Ö. Farkas, J. B. Foresman, J. V. Ortiz, J. Cioslowski, D. J. Fox, **2013**, DOI 111.

[7] M. J. Frisch, G. W. Trucks, H. B. Schlegel, G. E. Scuseria, M. A. Robb, J. R. Cheeseman, G. Scalmani, V. Barone, G. A. Petersson, H. Nakatsuji, X. Li, M. Caricato, A. V. Marenich, J. Bloino, B. G. Janesko, R. Gomperts, B. Mennucci, H. P. Hratchian, J. V. Ortiz, A. F. Izmaylov, J. L. Sonnenberg, D. Williams-Young, F. Ding, F. Lipparini, F.

Egidi, J. Goings, B. Peng, A. Petrone, T. Henderson, D. Ranasinghe, V. G. Zakrzewski, J. Gao, N. Rega, G. Zheng, W. Liang, M. Hada, M. Ehara, K. Toyota, R. Fukuda, J. Hasegawa, M. Ishida, T. Nakajima, Y. Honda, O. Kitao, H. Nakai, T. Vreven, K. Throssell, J. J. A. Montgomery, J. E. Peralta, F. Ogliaro, M. J. Bearpark, J. J. Heyd, E. N. Brothers, K. N. Kudin, V. N. Staroverov, T. A. Keith, R. Kobayashi, J. Normand, K. Raghavachari, A. P. Rendell, J. C. Burant, S. S. Iyengar, J. Tomasi, M. Cossi, J. M. Millam, M. Klene, C. Adamo, R. Cammi, J. W. Ochterski, R. L. Martin, K. Morokuma, O. Farkas, J. B. Foresman, and D. J. Fox, **2016**.

[8] D. Cremer, J. A. Pople, *J. Am. Chem. Soc.* **1975**, *97*, 1354–1358.

[9] J. J. Lee, S. Hesse, M. A. Suhm, *J. Mol. Struct.* **2010**, *976*, 397–404.

[10] Z. Kisiel, *Spectrosc. from Sp.* **2001**, 91–106.

[11] C. C. Costain, *J. Chem. Phys.* **1958**, *29*, 864–874.

[12] P. Pracht, F. Bohle, S. Grimme, *Phys. Chem. Chem. Phys.* **2020**, *22*, 7169–7192.

[13] T. A. Halgren, *J. Comput. Chem.* **1996**, *17*, 490–519.

[14] T. A. Halgren, *J. Comput. Chem.* **1999**, *20*, 720–729.

[15] I. Kolossváry, G. M. Keserü, *J. Comput. Chem.* **2001**, *22*, 21–30.

[16] C. Bannwarth, S. Ehlert, S. Grimme, *J. Chem. Theory Comput.* **2019**, *15*, 1652–1671.

[17] T. Lu, F. Chen, *J. Comput. Chem.* **2012**, *33*, 580–592.

[18] B. Jeziorski, R. Moszynski, K. Szalewicz, *Chem. Rev.* **1994**, *94*, 1887–1930.

[19] E. G. Hohenstein, C. D. Sherrill, *Wiley Interdiscip. Rev. Comput. Mol. Sci.* **2012**, *2*, 304–326.

[20] R. M. Parrish, L. A. Burns, D. G. A. Smith, A. C. Simmonett, A. E. DePrince, E. G. Hohenstein, U. Bozkaya, A. Y. Sokolov, R. Di Remigio, R. M. Richard, J. F. Gonthier, A. M. James, H. R. McAlexander, A. Kumar, M. Saitow, X. Wang, B. P. Pritchard, P. Verma, H. F. Schaefer, K. Patkowski, R. A. King, E. F. Valeev, F. A. Evangelista, J. M. Turney, T. D. Crawford, C. D. Sherrill, *J. Chem. Theory Comput.* **2017**, *13*, 3185–3197.

[21] D. A. Case, K. Belfon, I. Y. Ben-Shalom, S. R. Brozell, D. S. Cerutti, T. E. Cheatham, III, V. W. D. Cruzeiro, T. A. Darden, R. E. Duke, G. Giambasu, M. K. Gilson, H. Gohlke, A. W. Goetz, R. Harris, S. Izadi, S. A. Izmailov, K. Kasavajhala, A. Kovalenko, R. Krasny, T. Kurtzman, T. S. Lee, S. LeGrand, P. Li, C. Lin, J. Liu, T. Luchko, R. Luo, V. Man, K. M. Merz, Y. Miao, O. Mikhailovskii, G. Monard, H. Nguyen, A. Onufriev, F. Pan, S. Pantano, R. Qi, D. R. Roe, A. Roitberg, C. Sagui, S. Schott-Verdugo, J. Shen, C. L. Simmerling, N. R. Skrynnikov, J. Smith, J. Swails, R. C. Walker, J. Wang, L. Wilson, R. M. Wolf, X. Wu, Y. Xiong, Y. Xue, D. M. York, P. A. Kollman, *AMBER 2020*, University Of California, San Francisco, **2020**.

[22] J. Wang, R. M. Wolf, J. W. Caldwell, P. A. Kollman, D. A. Case, *J. Comput. Chem.* **2004**, *25*, 1157–1174.

[23] A. Jakalian, D. B. Jack, C. I. Bayly, *J. Comput. Chem.* **2002**, *23*, 1623–1641.

[24] W. L. Jorgensen, J. Chandrasekhar, J. D. Madura, R. W. Impey, M. L. Klein, *J. Chem. Phys.* **1983**, *79*, 926–935.

[25] T. Darden, D. York, L. Pedersen, *J. Chem. Phys.* **1993**, *98*, 10089–10092.

2. Broadband rotational spectrum

Figure S1. Sections of the broadband microwave spectrum of prolinol showing the $2_{0,2} \leftarrow 1_{0,1}$ rotational transition (parent, ^{13}C and ^{15}N isotopologues in their natural abundances) of prolinol **I** (left) and prolinol **II** (right). The experimental spectrum is shown in black trace (top) and the simulated spectrum of each isotopologue in colour (bottom) has been obtained using the experimental parameters determined from the fit and the program PGOPHER.

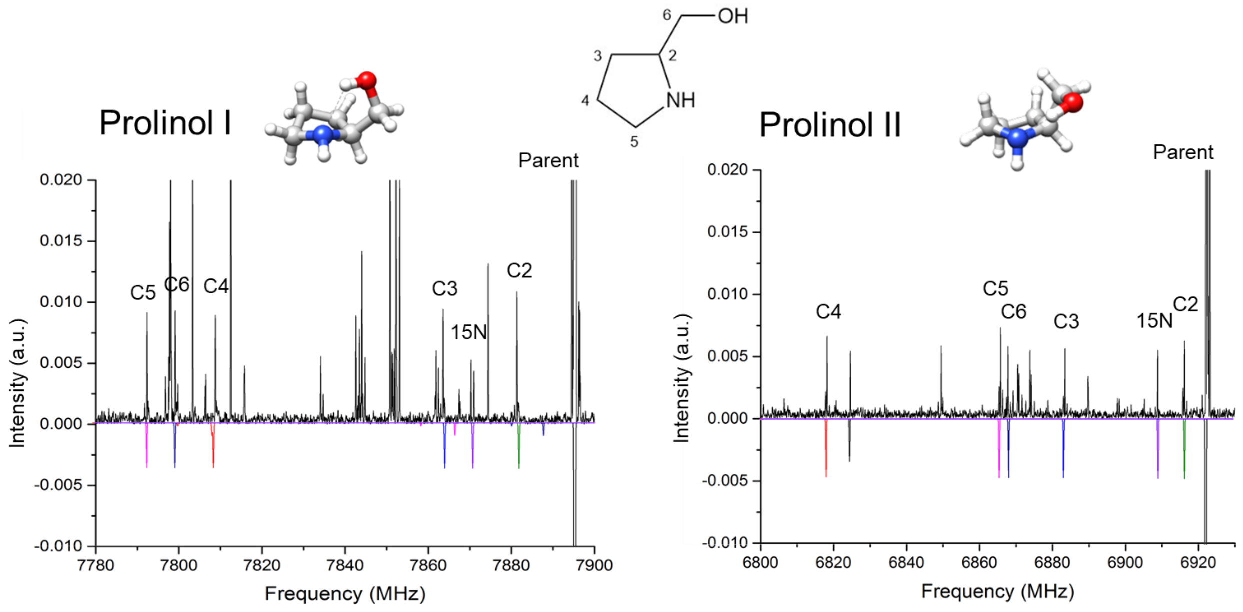

Figure S2. Broadband microwave spectrum of prolinol-water in the 2-8 GHz frequency range. Upper traces in black show the experimental spectrum, lower traces show the simulated spectrum of the observed species in different colours using the experimentally determined rotational constants. The inset shows an enlarged section to better show the least abundant species.

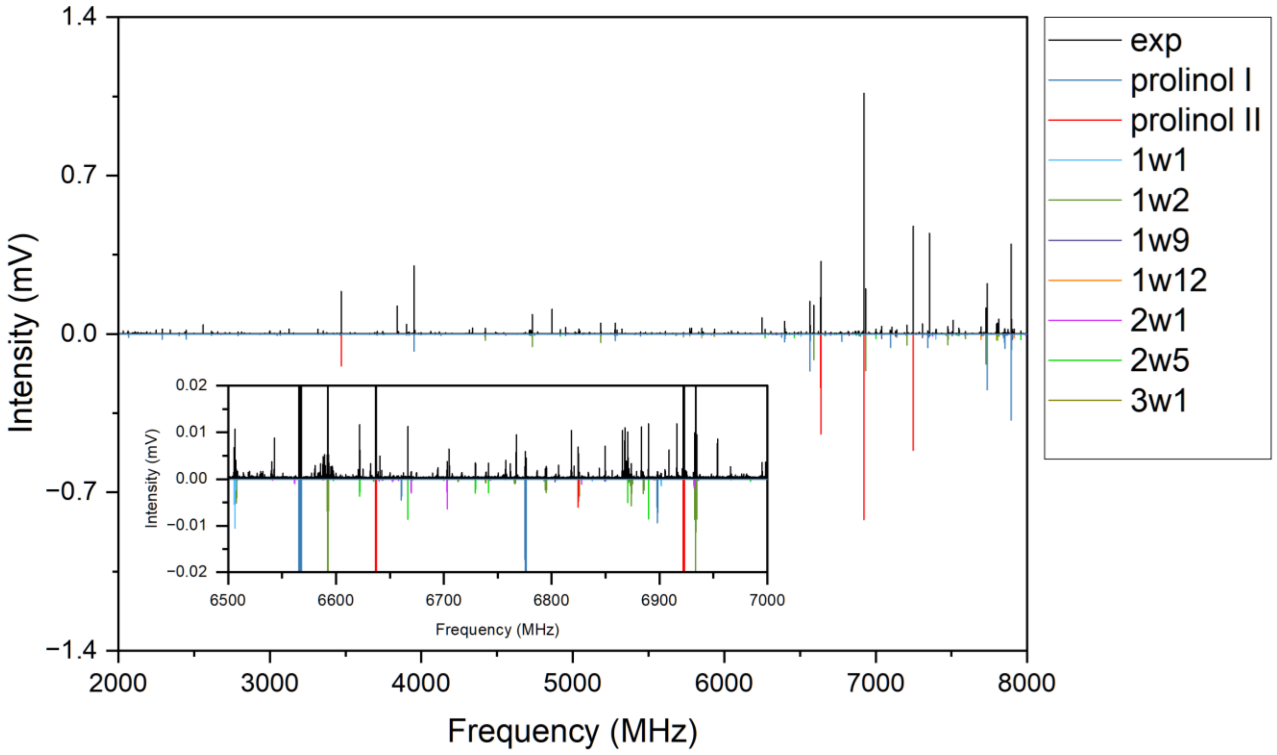

3. Spectroscopic parameters

3.1. Prolinol

Table S1. Predicted spectroscopic parameters and relative energies for the lower-energy conformers of prolinol at the MP2/6-311++G(d,p) and B3LYP-D3BJ/6-311++G(d,p) levels of theory.

	**I (E_5)**		**II (^5E)**		**III ($^3T_4$)**		**IV ($^4T_3$)**	
	B3LYP	MP2	B3LYP	MP2	B3LYP	MP2	B3LYP	MP2
A^a (MHz)	4489.2	4475.6	5198.4	5271.5	4771.9	4719.4	4794.5	4871.5
B (MHz)	2054.5	2089.3	1897.0	1898.1	1993.3	2033.6	1979.8	1978.0
C (MHz)	1888.1	1926.3	1585.4	1590.7	1764.9	1808.3	1663.2	1661.7
χ_{aa} (MHz)	1.13	1.07	-1.61	-1.44	-0.54	-0.82	0.03	0.37
χ_{bb} (MHz)	2.47	2.24	0.25	0.20	0.86	0.33	1.56	1.59
χ_{cc} (MHz)	-3.60	-3.31	1.36	1.25	-0.31	0.49	-1.59	-1.95
$\|\mu_a\|$ (D)	2.1	2.1	3.3	3.4	2.5	2.5	3.2	3.1
$\|\mu_b\|$ (D)	0.8	0.9	0.3	0.4	0.1	0.2	0.3	0.3
$\|\mu_c\|$ (D)	1.9	1.9	0.5	0.5	2.0	2.0	0.9	0.8
ΔE (cm^{-1})	0	12	30	0	131	130	176	218
ΔE_{ZPC} (cm^{-1})	0	0	63	23	108	107	175	203
ΔG^{373} (cm^{-1})	0	0	62	47	87	71	177	201
∠OCCN	-53.5	-55.0	55.1	57.2	-48.3	-48.7		50.6
N---O	2.79		2.81		2.76		2.76	
	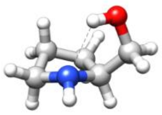		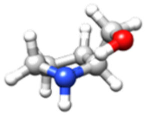		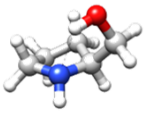		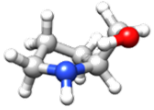	

	**V ($^1T_2$)**		**VI (^1E)**		**VII ($^1T_5$)**	
	B3LYP	MP2	B3LYP	MP2	B3LYP	MP2
A^a (MHz)	5692.4	5710.1	5789.6	5796.9	4745.0	4639.4
B (MHz)	1759.4	1769.9	1748.8	1767.0	1958.7	2021.4
C (MHz)	1443.1	1454.0	1439.1	1453.8	1765.1	1837.2
χ_{aa} (MHz)	0.82	0.68	2.89	2.64	2.82	2.61
χ_{bb} (MHz)	-2.09	-1.98	2.42	2.17	-2.99	-2.98
χ_{cc} (MHz)	1.27	1.30	-5.31	-4.80	0.17	0.36
$\|\mu_a\|$ (D)	2.8	2.8	0.1	0.1	1.3	1.3
$\|\mu_b\|$ (D)	0.8	0.9	1.0	1.0	0.9	0.9
$\|\mu_c\|$ (D)	0.9	0.9	0.1	0.1	0.3	0.2
ΔE (cm^{-1})	787	783	981	997	1085	1032
ΔE_{ZPC} (cm^{-1})	755	743	819	792	988	908
ΔG^{373} (cm^{-1})	653	603	674	584	794	716
∠OCCN		61.6		66.2	-59.1	-62.0
N--O	2.90		2.91		2.86	
	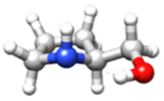		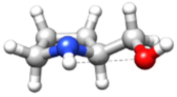		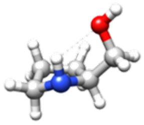	

[a] *A, B and C* are the rotational constants; χ_{aa}, χ_{bb}, and χ_{cc} are the ^{14}N nuclear quadrupole coupling constants; μ_a, μ_b and μ_c are the electric dipole moment components along the principal inertial axes; ΔE are the relative energies; ΔE_{ZPC} are the relative energies including zero-point corrections; ΔG^{373} are the free Gibbs energies at 373 K.

Table S2. Experimental spectroscopic parameters of the observed isotopic species of conformer **I** of prolinol.

Parameter	Parent	$^{13}C_5$	$^{13}C_4$	$^{13}C_3$	$^{13}C_2$
A[a] (MHz)	4507.02605(41)[f]	4486.53739(74)	4471.68125(69)	4440.61392(70)	4479.25588(62)
B (MHz)	2059.27298(20)	2033.03881(40)	2036.92932(33)	2055.76249(32)	2052.75206(35)
C (MHz)	1892.44146(20)	1867.22402(30)	1871.55673(24)	1880.69892(25)	1891.81677(30)
Δ_J[b] (kHz)	1.4677(46)	[1.4677][g]	[1.4677]	[1.4677]	[1.4677]
Δ_{JK} (kHz)	-7.022(13)	[-7.022]	[-7.022]	[-7.022]	[-7.022]
Δ_K (kHz)	13.345(41)	[13.345]	[13.345]	[13.345]	[13.345]
δ_J (kHz)	-0.1627(15)	[-0.1627]	[-0.1627]	[-0.1627]	[-0.1627]
χ_{aa}[c] (MHz)	1.1275(20)	1.1539(44)	1.1514(34)	1.1118(33)	1.1377(39)
χ_{bb} (MHz)	2.1608(29)	2.1505(62)	2.1703(53)	2.1573(50)	2.1660(60)
χ_{cc} (MHz)	-3.2883(29)	-3.3043(62)	-3.3217(53)	-3.2691(50)	-3.3036(60)
N[d]	346	70	68	70	80
σ[e] (kHz)	6.9	7.0	5.9	5.7	7.0

Parameter	$^{13}C_6$	^{15}N	^{18}O
A (MHz)	4498.64149(83)	4456.58184(75)	4442.61(29)
B (MHz)	2032.34772(34)	2057.80008(42)	1987.21073(96)
C (MHz)	1871.06168(28)	1882.01531(34)	1842.27234(95)
Δ_J (kHz)	[1.4677]	[1.4677]	[1.4677]
Δ_{JK} (kHz)	[-7.022]	[-7.022]	[-7.022]
Δ_K (kHz)	[13.345]	[13.345]	[13.345]
δ_J (kHz)	[-0.1627]	[-0.1627]	[-0.1627]
χ_{aa} (MHz)	1.1695(41)	-	0.9879(73)
χ_{bb} (MHz)	2.1561(59)	-	2.139(14)
χ_{cc} (MHz)	-3.3255(59)	-	-3.127(14)
N	72	22	25
σ (kHz)	6.8	5.0	7.0

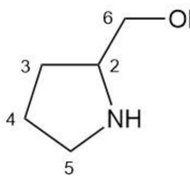

[a] A, B and C are the rotational constants. [b] Δ_J Δ_{JK} Δ_K and δ_J are the centrifugal distortion constants. [c] χ_{aa}, χ_{bb}, and χ_{cc} are the ^{14}N nuclear quadrupole coupling constants. [d] N is the number of fitted hyperfine components. [e] σ is the rms deviation of the fit. [f] Standard error in parentheses in units of the last digit. [g] Parameters in brackets are fixed to the value of the parent species.

Table S3. Experimental spectroscopic parameters of the observed isotopic species of conformer **II** of prolinol.

Parameter	Parent	$^{13}C_5$	$^{13}C_4$	$^{13}C_3$	$^{13}C_2$
A[a] (MHz)	5241.6960(14)[f]	5173.721(83)	5238.3563(49)	5158.328(67)	5219.113(69)
B (MHz)	1888.2816(12)	1874.28261(89)	1857.45828(70)	1881.65014(85)	1886.05244(87)
C (MHz)	1582.9077(10)	1568.69796(76)	1560.95492(56)	1570.58962(77)	1581.94117(72)
Δ_J[b] (kHz)	0.624(16)	[0.624][g]	[0.624]	[0.624]	[0.624]
Δ_{JK} (kHz)	-2.451(89)	[-2.451]	[-2.451]	[-2.451]	[-2.451]
Δ_K (kHz)	7.75(30)	[-7.75]	[-7.75]	[-7.75]	[-7.75]
δ_J (kHz)	0.0442(61)	[0.0442]	[0.0442]	[0.0442]	[0.0442]
δ_K (kHz)	1.11(49)	[1.11]	[1.11]	[1.11]	[1.11]
χ_{aa}[c] (MHz)	-1.3383(31)	-1.2907(73)	-1.3515(55)	-1.3926(66)	-1.3365(57)
χ_{bb} (MHz)	0.1920(48)	0.167(16)	0.202(11)	0.240(12)	0.17812)
χ_{cc} (MHz)	1.1464(48)	1.124(16)	1.149(11)	1.152(12)	1.159(12)
N[d]	203	41	52	48	44
σ[e] (kHz)	8.3	9.4	7.8	9.4	7.6

Parameter	$^{13}C_6$	^{15}N	^{18}O
A (MHz)	5214.401(95)	5175.112(51)	5233.10(26)
B (MHz)	1872.61246(96)	1887.00365(60)	1804.6676(14)
C (MHz)	1571.02386(78)	1577.80250(60)	1523.1041(16)
Δ_J (kHz)	[0.624]	[0.624]	[0.624]
Δ_{JK} (kHz)	[-2.451]	[-2.451]	[-2.451]
Δ_K (kHz)	[-7.75]	[-7.75]	[-7.75]
δ_J (kHz)	[0.0442]	[0.0442]	[0.0442]
δ_K (kHz)	[1.11]	[1.11]	[1.11]
χ_{aa} (MHz)	-1.3209(62)	-	-1.387(17)
χ_{bb} (MHz)	0.169(12)	-	[0.216][h]
χ_{cc} (MHz)	1.151(12)	-	[1.171][h]
N	42	14	11
σ (kHz)	8.4	3.9	8.1

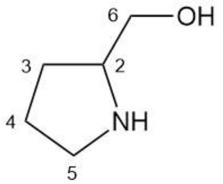

[a] A, B and C are the rotational constants. [b] Δ_J Δ_{JK} Δ_K, δ_J and δ_K are the centrifugal distortion constants. [c] χ_{aa}, χ_{bb}, and χ_{cc} are the ^{14}N nuclear quadrupole coupling constants. [d] N is the number of fitted hyperfine components. [e] σ is the rms deviation of the fit. [f] Standard error in parentheses in units of the last digit. [g] Parameters in brackets are fixed to the value of the parent species. [h] The value of χ_{bb}- χ_{cc} could not be determined from the fit and was kept fixed to the parent species' value of -0.9548(64) MHz. The values of χ_{bb} and χ_{cc} indicated in square brackets are derived from the value of χ_{aa} determined from the fit and the fixed value of χ_{bb}- χ_{cc}.

Table S4. Experimental substitution (r_s) and effective (r_0) structures of conformer **I** of prolinol, and their comparison with the equilibrium ones at the B3LYP-D3BJ/6-311G++G(d,p) and MP2/6-311G++G(d,p) levels of theory. Bond lengths are in angstroms (Å) and angles are in degrees (°).

Parameter	r_s	r_0	B3LYP	MP2
$r(C_2\text{-}C_3)$	1.499(29)	1.547(12)	1.553	1.550
$r(C_3\text{-}C_4)$	1.573(4)	1.550(12)	1.542	1.539
$r(C_4\text{-}C_5)$	1.531(3)	1.537(11)	1.529	1.526
$r(N\text{-}C_2)$	1.564(26)	1.490(17)[a]	1.480	1.479
$r(N\text{-}C_5)$	1.417(4)	1.441(14)	1.467	1.468
$r(C_6\text{-}C_2)$	1.504(5)	1.521(14)	1.530	1.527
$r(O\text{-}C_6)$	1.400(6)	1.387(9)	1.418	1.416
$\angle(C_2\text{-}C_3\text{-}C_4)$	105.7(7)	105.8(7)	105.5	105.1
$\angle(C_3\text{-}C_4\text{-}C_5)$	102.2(3)	102.6(5)	103.2	102.4
$\angle(N\text{-}C_2\text{-}C_3)$	103.1(4)	103.4(8)[a]	104.2	104.3
$\angle(C_6\text{-}C_2\text{-}N)$	108.0(15)	110.7(8)	108.5	108.2
$\angle(O\text{-}C_6\text{-}C_2)$	111.2(3)	112.0(4)	111.0	110.5
$\tau(C_2\text{-}C_3\text{-}C_4\text{-}C_5)$	-26.5(4)	-25.8(8)	-24.0	-27.0
$\tau(N\text{-}C_2\text{-}C_3\text{-}C_4)$	1.3(5)	3.7(11)[a]	0.5	2.4
$\tau(O\text{-}C_6\text{-}C_2\text{-}N)$	-51.5(49)	-53.5(14)[a]	-53.5	-55.0

[a] Parameter derived from the fit, from the values of the principal inertial axis coordinates and their estimated uncertainties.

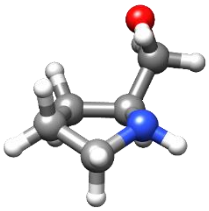

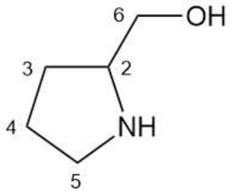

Table S5. Experimental substitution and effective structures of conformer **II** of prolinol, and their comparison with the equilibrium ones at the B3LYP-D3BJ/6-311G++G(d,p) and MP2/6-311G++G(d,p) levels of theory. Bond lengths are in angstroms (Å) and angles are in degrees (°).

Parameter	r_s	r_0	B3LYP	MP2
$r(C_2\text{-}C_3)$	1.563(15)	1.541(12)	1.556	1.553
$r(C_3\text{-}C_4)$	1.532(5)	1.554(8)	1.543	1.539
$r(C_4\text{-}C_5)$	1.539(7)	1.523(10)	1.537	1.533
$r(N\text{-}C_2)$	1.431(5)	1.469(12)[a]	1.485	1.484
$r(N\text{-}C_5)$	1.612(11)	1.498(11)	1.473	1.473
$r(C_6\text{-}C_2)$	1.553(6)	1.540(13)	1.529	1.525
$r(O\text{-}C_6)$	1.416(6)	1.423(6)	1.416	1.415
$\angle(C_2\text{-}C_3\text{-}C_4)$	102.3(7)	104.3(5)	104.9	104.5
$\angle(C_3\text{-}C_4\text{-}C_5)$	101.3(4)	102.5(2)	103.0	102.2
$\angle(N\text{-}C_2\text{-}C_3)$	114.7(7)	109.3(9)[a]	107.2	107.4
$\angle(C_6\text{-}C_2\text{-}N)$	102.6(5)	107.0(11)	108.1	107.7
$\angle(O\text{-}C_6\text{-}C_2)$	111.9(7)	111.2(7)[a]	111.2	111.0
$\tau(C_2\text{-}C_3\text{-}C_4\text{-}C_5)$	32.(2)	23.8(7)	23.2	26.4
$\tau(N\text{-}C_2\text{-}C_3\text{-}C_4)$	-14.(3)	-3.9(7)[a]	-2.9	-5.3
$\tau(O\text{-}C_6\text{-}C_2\text{-}N)$	51.6(8)	55.8(9)	55.2	57.2

[a] Parameter derived from the fit, from the values of the principal inertial axis coordinates and their estimated uncertainties.

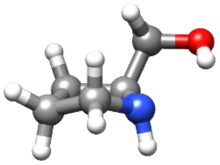

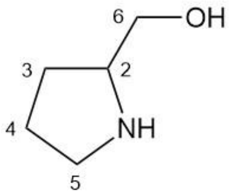

Table S6. Substitution coordinates of the heavy atoms of conformer **I** of prolinol in Å. Signs taken from the theoretical calculations.

	a	*b*	*c*
N_1	0.4400(34)[a]	-1.1428(13)	0.136i(11)[b]
C_2	-0.2941(51)	0.043(35)	-0.8356(18)
C_3	0.4511(33)	1.2083(12)	-0.4802(31)
C_4	1.54703(97))	0.7702(20)	0.5604(27)
C_5	1.77178(85)	-0.6996(21)	0.1948(77)
C_6	-1.75006(86)	0.068(22)	-0.4581(33)
O_7	-1.91362(92)	0.018i(99)[b]	0.9308(19)

[a] Errors in parentheses, including Costain's error, in units of the last digit.
[b] Imaginary coordinates were set to zero for the determination of the r_s structure.

Table S7. Substitution coordinates of the heavy atoms of conformer **II** of prolinol in Å. Signs taken from the theoretical calculations.

	a	*b*	*c*
N_1	-0.115i(13)[a,b]	1.0274(15)	0.4481(35)
C_2	0.2175(71)	-0.3850(40)	0.5214(30)
C_3	-0.9706(16)	-1.2564(12)	0.037i(42)[b]
C_4	-2.11246(71)	-0.2386(63)	-0.081(19)
C_5	-1.3457(12)	1.0462(15)	-0.4400(35)
C_6	1.4432(11)	-0.5877(27)	-0.4102(38)
O_7	2.50962(65)	0.2832(85)	-0.078(21)

[a] Errors in parentheses, including Costain's error, in units of the last digit.
[b] Imaginary coordinates were set to zero for the determination of the r_s structure.

3.2. Prolinol-H_2O

Table S8. Theoretical spectroscopic parameters and relative energies at the B3LYP-D3BJ/6-311++G(d,p) and MP2/6-311++G(d,p) levels of theory for the lower-energy isomers of prolinol-H_2O.

**Isomer**	**1w1**		**1w2**		**1w3**		**1w4**		**1w5**	
	B3LYP	MP2	B3LYP	MP2	B3LYP	MP2	B3LYP	MP2	B3LYP	MP2
A^a (MHz)	2207.3	2199.6	2740.9	2753.8	2252.9	2270.3	2756.5	2764.9	2536.8	2534.4
B (MHz)	1736.1	1766.5	1392.0	1399.5	1664.9	1700.4	1367.6	1374.2	1476.6	1483.8
C (MHz)	1300.3	1319.8	1011.8	1020.9	1255.9	1288.8	993.6	1000.4	1110.8	1128.3
χ_{aa} (MHz)	-2.60	-2.86	-3.29	-3.13	-1.87	-2.83	-2.93	-2.95	-0.67	-0.52
χ_{bb} (MHz)	1.43	1.67	1.75	1.62	1.45	2.07	1.84	1.73	1.14	0.94
χ_{cc} (MHz)	1.18	1.19	1.54	1.51	0.42	0.76	1.09	1.22	-0.47	-0.42
$|\mu_a|$ (D)	2.3	2.2	3.8	3.8	3.6	3.1	4.5	4.5	3.6	3.6
$|\mu_b|$ (D)	0.8	1.0	1.8	1.9	1.2	1.9	1.5	1.7	1.4	1.4
$|\mu_c|$ (D)	3.4	3.4	1.3	1.1	1.4	1.7	1.0	1.0	1.2	1.1
ΔE (cm^{-1})	0	0	27.2	48.3	56.7	86.2	83.4	138.5	222.0	222.1
ΔE_{ZPC} (cm^{-1})	0	0	17.7	7.7	24.0	45.1	53.9	90.4	188.0	163.3

**Isomer**	**1w6**		**1w7**		**1w8**		**1w9**		**1w10**	
	B3LYP	MP2	B3LYP	MP2	B3LYP	MP2	B3LYP	MP2	B3LYP	MP2
A^a (MHz)	2549.6	2544.3	2633.8	2627.3	2741.3	2733.1	2375.4	2475.2	2685.6	2685.5
B (MHz)	1445.4	1447.4	1458.7	1476.9	1326.7	1327.7	1479.5	1427.8	1368.3	1371.6
C (MHz)	1077.5	1087.1	1078.3	1095.4	943.0	943.6	1154.5	1130.1	1046.0	1067.1
χ_{aa} (MHz)	-0.25	0.03	-4.19	-3.99	-1.57	-1.53	-0.99	-0.51	-0.80	-0.62
χ_{bb} (MHz)	1.48	1.27	2.11	2.03	0.25	0.31	2.52	2.29	-0.99	-1.09
χ_{cc} (MHz)	-1.23	-1.30	2.08	1.96	1.32	1.22	-1.52	-1.76	1.78	1.71
$|\mu_a|$ (D)	4.2	4.1	3.5	3.4	4.0	4.0	2.4	2.5	3.1	2.9
$|\mu_b|$ (D)	0.8	0.8	1.8	2.0	1.3	1.4	1.5	1.6	1.1	1.1
$|\mu_c|$ (D)	0.9	1.0	1.7	1.6	0.2	0.1	0.4	0.5	0.4	0.2
ΔE (cm^{-1})	258.7	301.4	348.0	396.7	410.5	517.0	804.0	593.0	740.4	647.4
ΔE_{ZPC} (cm^{-1})	216.2	225.4	355.9	397.6	374.3	471.2	633.3	412.0	698.9	630.5

**Isomer**	**1w11**		**1w12**		**1w13**		**1w14**	
	B3LYP	MP2	B3LYP	MP2	B3LYP	MP2	B3LYP	MP2
A^a (MHz)	2612.1	2592.7	3297.9	3382.8	2932.2	2884.7	2226.4	2225.8
B (MHz)	1383.5	1383.9	1016.0	1013.9	1214.6	1241.4	1466.7	1464.5
C (MHz)	1056.2	1077.8	984.1	958.1	1059.3	1065.0	1175.2	1169.4
χ_{aa} (MHz)	0.52		0.72	0.79	2.05	1.96	-2.22	-1.67
χ_{bb} (MHz)	-2.01		-2.99	-2.76	1.14	0.93	2.38	1.90
χ_{cc} (MHz)	1.49		2.27	1.96	-3.19	-2.88	-0.16	-0.22
$|\mu_a|$ (D)	3.2	3.0	2.3	2.4	1.4	1.2	2.2	2.2
$|\mu_b|$ (D)	0.6	0.6	0.0	0.1	0.1	0.2	1.7	1.6
$|\mu_c|$ (D)	0.7	0.5	0.3	0.2	0.4	0.3	0.5	0.6
ΔE (cm^{-1})	882.1	886.2	989.6	729.2	993.3	737.9	993.1	726.8
ΔE_{ZPC} (cm^{-1})	799.8	815.3	828.8	541.3	841.8	591.1	842.6	583.2

[a] *A, B and C* are the rotational constants; χ_{aa}, χ_{bb}, and χ_{cc} are the ^{14}N nuclear quadrupole coupling constants; μ_a, μ_b and μ_c are the electric dipole moment components along the principal inertial axes; ΔE are the relative energies; ΔE_{ZPC} are the relative energies including zero-point corrections.

Figure S3. Structures of the lower-energy isomers of prolinol-H_2O.

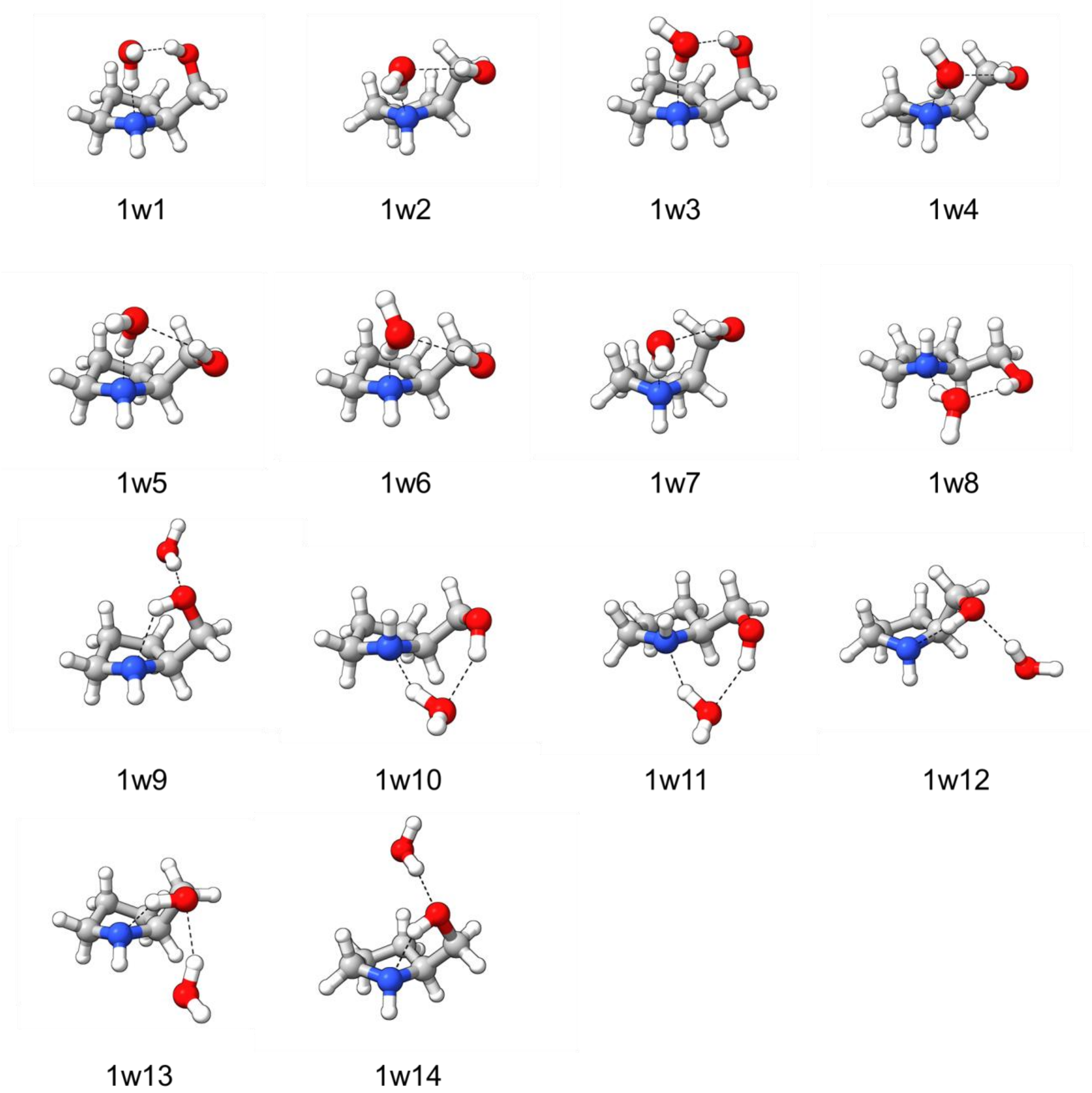

3.3. Prolinol-$(H_2O)_2$

Table S9. Theoretical spectroscopic parameters and relative energies at the B3LYP-D3BJ/6-311++G(d,p) and MP2/6-311++G(d,p) levels of theory for the lower-energy isomers of prolinol-$(H_2O)_2$.

Isomer	2w1		2w2		2w3		2w4		2w5	
	B3LYP	MP2	B3LYP	MP2	B3LYP	MP2	B3LYP	MP2	B3LYP	MP2
A^a (MHz)	1938.8	1950.9	1825.0	1817.4	1941.5	1947.4	1718.3	1706	1914.9	1950.5
B (MHz)	932.3	929.8	980.6	986.9	929.0	928.0	1012.1	1020	937.7	923.5
C (MHz)	723.4	723.4	758.5	764.0	708.2	705.3	774.9	780.9	750.4	750.4
χ_{aa} (MHz)	-0.69	-0.74	-0.20	-0.28	-0.69	-0.73	1.21	1.40	0.88	0.84
χ_{bb} (MHz)	-0.23	-0.25	-1.63	-1.56	-0.05	-0.03	-3.13	-3.33	-0.66	-0.89
χ_{cc} (MHz)	0.92	0.99	1.83	1.84	0.74	0.76	1.92	1.94	-0.22	0.06
$\|\mu_a\|$ (D)	1.0	0.9	1.4	1.4	1.5	1.3	1.7	1.6	2.7	2.5
$\|\mu_b\|$ (D)	2.2	2.2	1.5	1.6	1.9	1.9	1.4	1.5	2.1	2.1
$\|\mu_c\|$ (D)	0.8	0.9	0.4	0.3	1.0	1.0	0.5	0.5	0.6	0.7
ΔE (cm^{-1})	0	16.7	24.2	0	55.6	48.3	105.8	92.2	89.7	104.6
ΔE_{ZPC} (cm^{-1})	0	0	16.5	26.1	19.5	8.8	66.7	88.9	44.3	65.4
**Isomer**	**2w6**		**2w7**		**2w8**		**2w9**		**2w10**	
	B3LYP	MP2	B3LYP	MP2	B3LYP	MP2	B3LYP	MP2	B3LYP	MP2
A^a (MHz)	1975.3	1999.9	1915.4	1921.2	1730.2	1707.3	1995.1	2118.7	1985.9	2113.5
B (MHz)	921.5	916.7	925.5	920.3	997.9	1007.8	936.0	910.4	936.1	906.6
C (MHz)	715.0	712.5	735.7	736.8	762.6	769.1	773.3	756.8	780.9	761.6
χ_{aa} (MHz)	-0.62	-0.65	0.77	0.81	1.26	1.42	-4.56	-4.18	-4.57	-4.17
χ_{bb} (MHz)	-0.35	-0.40	-0.33	-0.49	-3.01	-3.17	2.23	2.02	2.19	1.98
χ_{cc} (MHz)	0.97	1.05	-0.44	-0.32	1.75	1.75	2.34	2.16	2.37	2.19
$\|\mu_a\|$ (D)	1.4	1.3	2.9	2.7	2.6	2.5	1.8	1.6	1.6	1.6
$\|\mu_b\|$ (D)	2.9	2.8	1.7	1.8	1.4	1.5	2.4	2.1	2.5	2.1
$\|\mu_c\|$ (D)	0.7	0.7	0.4	0.5	1.2	1.2	1.7	1.7	1.8	1.9
ΔE (cm^{-1})	174.6	139.3	119.7	144.7	99.2	148.9	422.8	301.9	424.9	327.9
ΔE_{ZPC} (cm^{-1})	84.1	59.5	53.1	83.0	87.4	157.4	414.1	322.2	426.0	345.0

[a] *A, B and C* are the rotational constants; χ_{aa}, χ_{bb} , and χ_{cc} are the ^{14}N nuclear quadrupole coupling constants; μ_a, μ_b and μ_c are the electric dipole moment components along the principal inertial axes; ΔE are the relative energies; ΔE_{ZPC} are the relative energies including zero-point corrections.

Figure S4. Structures of the lower-energy isomers of prolinol-$(H_2O)_2$.

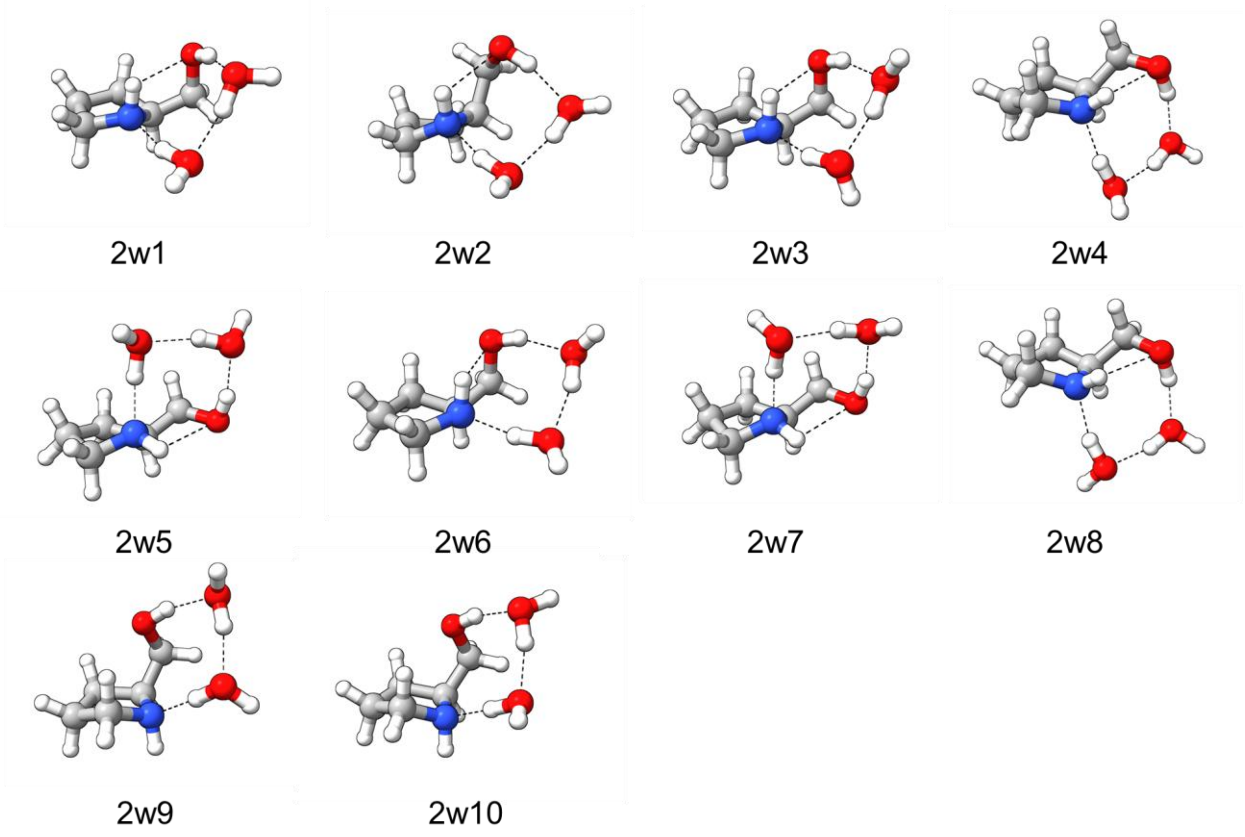

3.4. Prolinol-(H_2O)$_3$

Table S10. Theoretical spectroscopic parameters and relative energies at the B3LYP-D3BJ/6-311++G(d,p) and MP2/6-311++G(d,p) levels of theory for the lower-energy isomers of prolinol-(H_2O)$_3$.

**Isomer**	**3w1**		**3w2**		**3w3**		**3w4**		**3w5**	
	B3LYP	MP2	B3LYP	MP2	B3LYP	MP2	B3LYP	MP2	B3LYP	MP2
A[a] (MHz)	1302.5	1317.0	1173.8	1164.4	1142.8	1119.2	1195.5	1173.6	1130.5	1112.4
B (MHz)	710.3	708.7	756.6	764.5	757.1	771.3	768.6	780.8	775.6	790.0
C (MHz)	596.0	596.9	552.9	557.0	690.7	688.8	663.1	656.0	680.7	677.7
χ_{aa} (MHz)	-1.12	-1.13	1.63	1.65	1.22	1.02	1.24	1.21	1.15	0.98
χ_{bb} (MHz)	-0.01	-0.41	-3.77	-3.67	-2.00	-2.04	-3.62	-3.49	-2.39	-2.32
χ_{cc} (MHz)	1.13	1.55	2.14	2.02	0.78	1.03	2.38	2.28	1.24	1.35
$\|\mu_a\|$ (D)	0.1	0.3	1.5	1.4	1.0	0.9	1.3	1.1	0.9	0.9
$\|\mu_b\|$ (D)	2.1	1.9	1.7	1.8	1.8	1.7	1.8	1.7	1.6	1.5
$\|\mu_c\|$ (D)	0.6	0.5	0.4	0.5	0.6	0.4	0.3	0.3	0.2	0.0
ΔE (cm^{-1})	48	0	143	42	88	48	160	123	150	82
ΔE_{ZPC} (cm^{-1})	0	0	42	67	71	65	92	100	105	88
Isomer	**3w6**		**3w7**		**3w8**		**3w9**		**3w10**	
	B3LYP	MP2	B3LYP	MP2	B3LYP	MP2	B3LYP	MP2	B3LYP	MP2
A [a] (MHz)	1247.7	1239.4	1429.2	1409.5	1334.9	1306.9	1311.8	1293.4	1199.2	1193.6
B (MHz)	714.4	717.9	631.9	639.6	715.2	722.2	726.0	726.4	735.9	736.0
C (MHz)	536.6	540.4	487.7	493.7	662.3	672.1	674.8	678.6	535.1	537.2
χ_{aa} (MHz)	0.24	0.04	-0.27	-0.16	-1.51	-1.60	0.47	0.65	1.09	1.17
χ_{bb} (MHz)	-2.29	-1.98	-1.44	-1.56	2.05	2.13	1.77	2.03	-2.79	-2.83
χ_{cc} (MHz)	2.05	1.94	1.71	1.71	-0.54	-0.53	-2.45	-2.68	1.71	1.67
$\|\mu_a\|$ (D)	1.5	1.4	1.2	0.9	1.7	1.6	1.3	1.2	1.6	1.5
$\|\mu_b\|$ (D)	1.6	1.6	2.3	2.3	1.3	1.1	1.2	1.0	1.1	1.2
$\|\mu_c\|$ (D)	0.6	0.7	0.3	0.3	1.4	1.3	1.5	1.6	1.1	1.1
ΔE (cm^{-1})	202	145	277	118	0	85	17	179	316	271
ΔE_{ZPC} (cm^{-1})	110	178	151	129	154	226	165	315	174	248
Isomer	**3w11**		**3w12**		**3w13**		**3w14**		**3w15**	
	B3LYP	MP2	B3LYP	MP2	B3LYP	MP2	B3LYP	MP2	B3LYP	MP2
A [a] (MHz)	1339.1	1313.3	1313.2	1278.4	1169.7	1166.7	1262.1	1249.0	1256.9	1227.2
B (MHz)	699.6	706.9	719.9	725.8	845.6	836.7	727.1	724.8	783.9	794.2
C (MHz)	643.9	654.1	652.7	668.0	759.7	772.4	593.2	588.8	672.3	672.6
χ_{aa} (MHz)	-2.51	-2.39	1.06	0.69	-3.56	-3.65	1.05	0.91	-0.56	-0.68
χ_{bb} (MHz)	1.60	1.76	2.74	2.43	2.65	2.73	0.29	0.15	-0.14	-0.24
χ_{cc} (MHz)	0.91	0.63	-3.79	-3.12	0.91	0.92	-1.34	-1.07	0.70	0.92
$\|\mu_a\|$ (D)	1.8	1.9	1.3	1.2	1.5	1.5	1.3	1.2	3.3	3.0
$\|\mu_b\|$ (D)	1.5	1.3	0.1	0.3	0.2	0.0	2.2	2.3	0.6	0.6
$\|\mu_c\|$ (D)	1.1	1.4	2.9	2.8	1.7	1.7	0.8	-0.7	2.2	2.3
ΔE (cm^{-1})	13	232	78	232	59	371	304	307	171	261
ΔE_{ZPC} (cm^{-1})	179	389	227	256	237	483	245	301	246	239

[a] *A, B and C* are the rotational constants; χ_{aa}, χ_{bb}, and χ_{cc} are the ^{14}N nuclear quadrupole coupling constants; μ_a, μ_b and μ_c are the electric dipole moment components along the principal inertial axes; ΔE are the relative energies; ΔE_{ZPC} are the relative energies including zero-point corrections.

Figure S5. Structures of the lower-energy isomers of prolinol-$(H_2O)_3$.

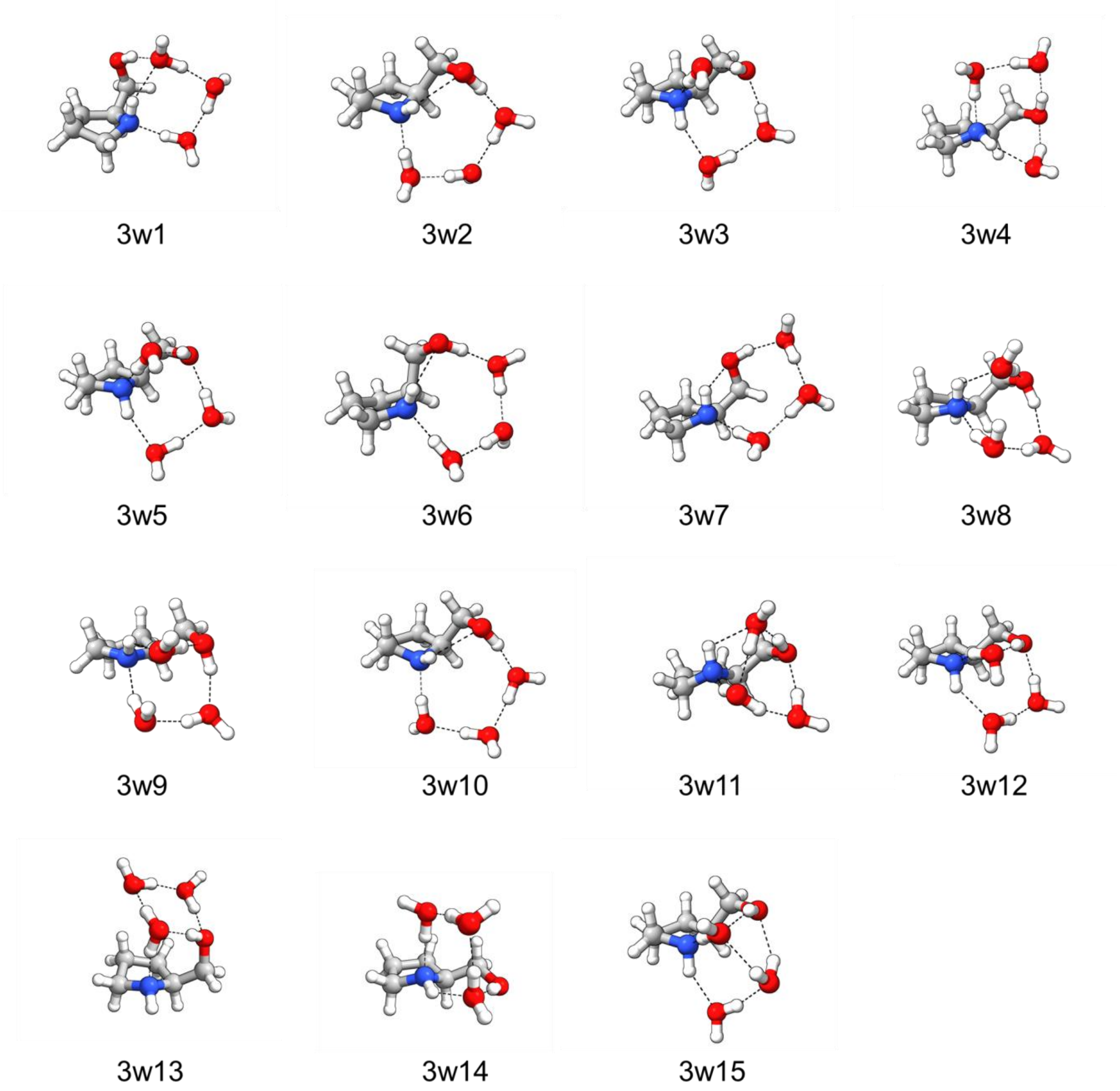

4. Interconversion barriers

4.1. Prolinol

Figure S6. Interconversion barriers between prolinol conformers **III** ↔ **I** (top) and **IV** ↔ **II** (bottom) at the B3LYP-D3BJ/6-311++G(d,p) level of theory.

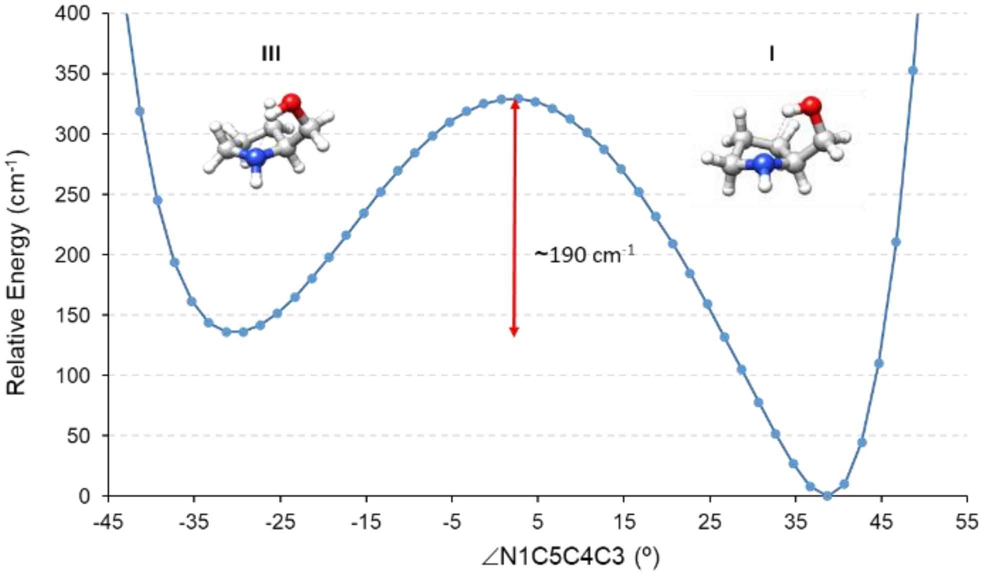

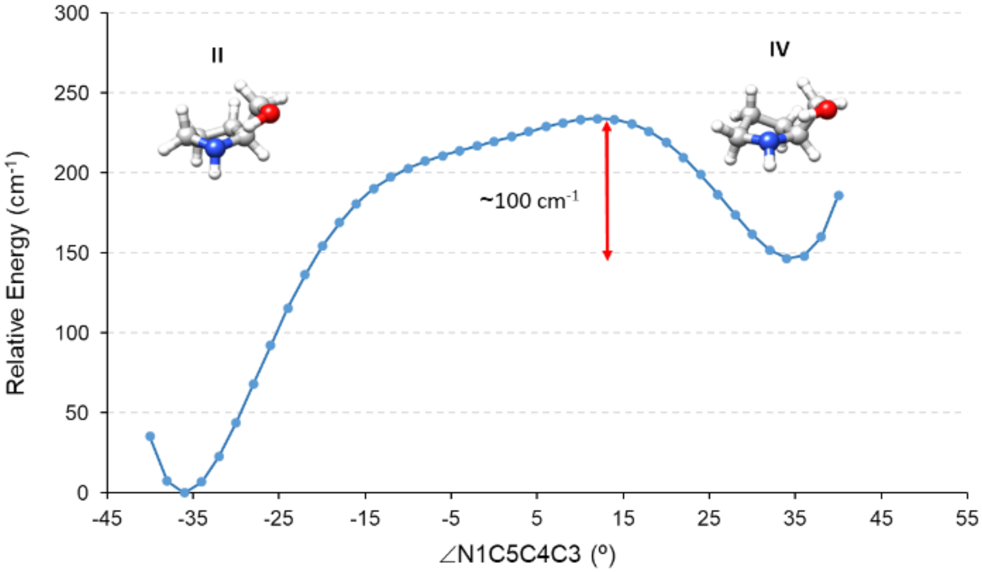

4.2. Prolinol-H_2O

Non-observation of several low-energy isomers of prolinol-H_2O can be explained in terms of collisional relaxation in the early stages of the supersonic expansion. Isomers **1w3** and **1w4** only differ from the observed complexes **1w1** and**1w2** by the orientation of the non-bonding hydrogen atom of the water molecule. A conformational interconversion path from **1w3** to **1w1** and from **1w4** to **1w2** yielded barriers of *ca*. 200 cm^{-1}, thus explaining their absence. Isomers **1w7** and **1w5** differ from **1w2** in the puckering of the ring, and they are expected to relax in the supersonic expansion to isomer **1w2**. Finally, the barrier for the conversion of isomer **1w6** to **1w5** was calculated to be 210 cm^{-1}. The conformational interconversion barriers are shown below.

Figure S7. Interconversion barriers between the isomers of prolinol-H_2O **1w3** ↔ **1w1** (top) and **1w4** ↔ **1w2** (bottom) at the B3LYP-D3BJ/6-311++G(d,p) level of theory.

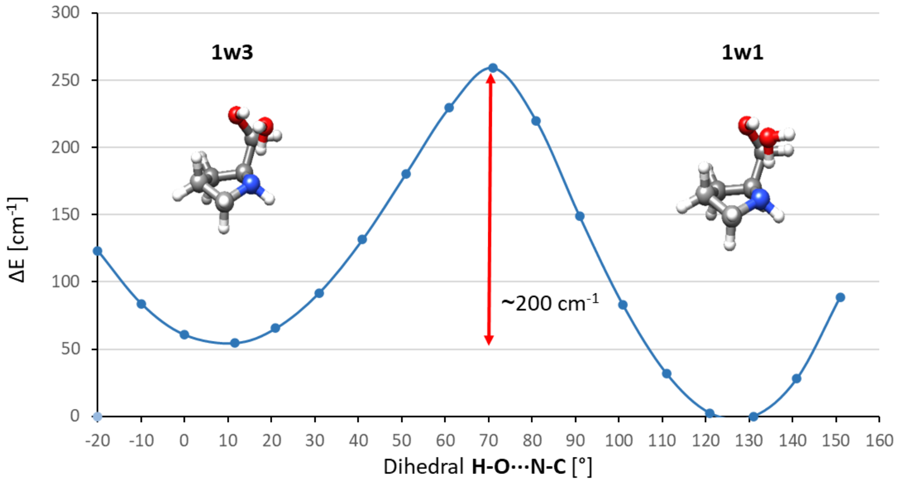

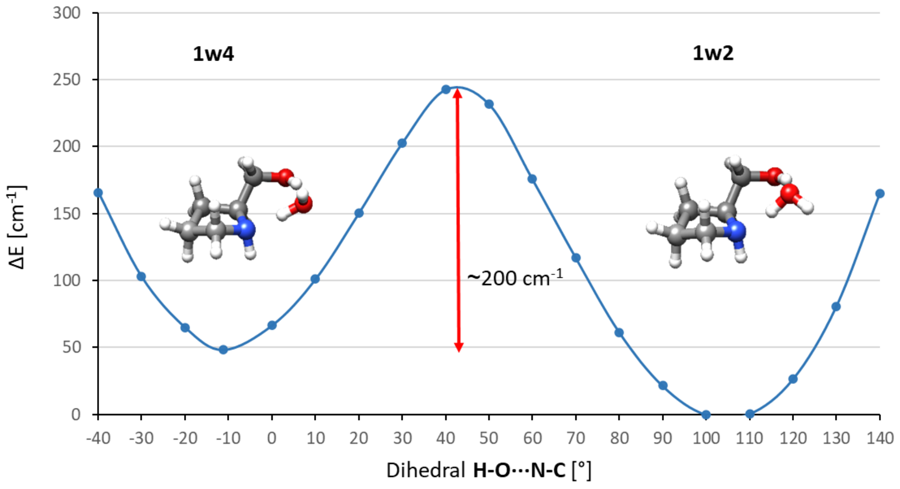

Figure S8. Interconversion barriers between the isomers of prolinol-H_2O: , **1w5** ↔ **1w2** (top), **1w6** ↔ **1w5** (middle) and **1w5** ↔ **1w7** ↔ **1w2** (bottom) at the B3LYP-D3BJ/6-311++G(d,p) level of theory.

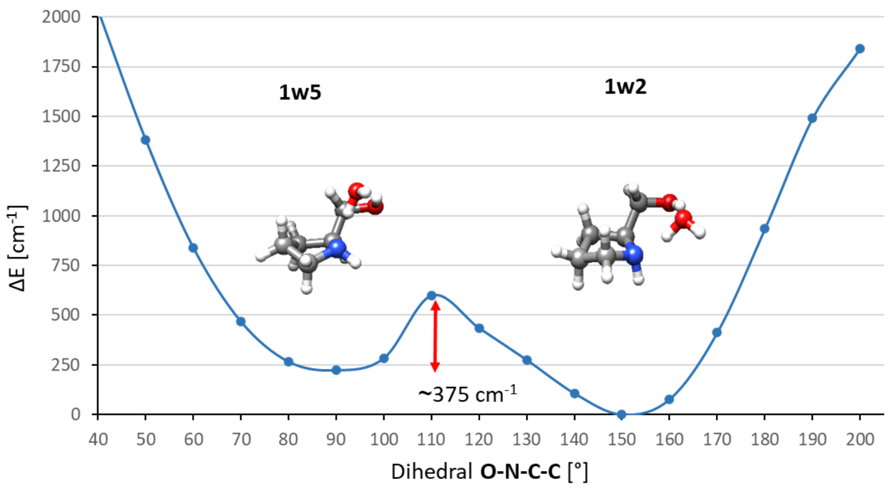

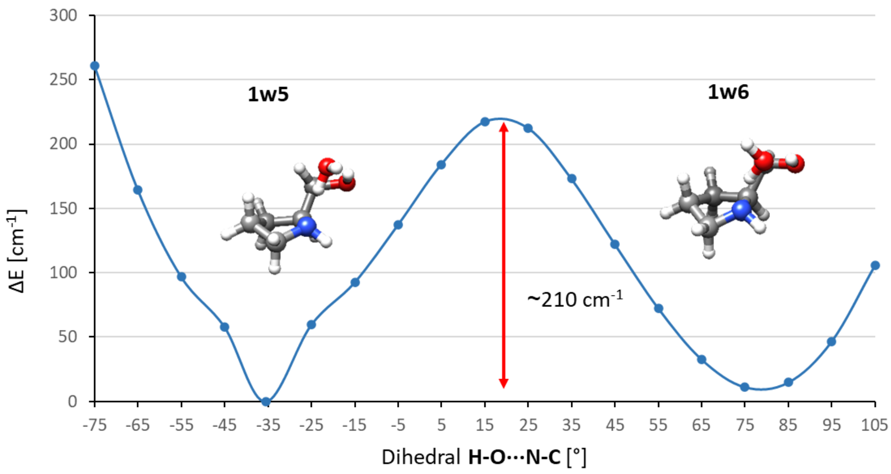

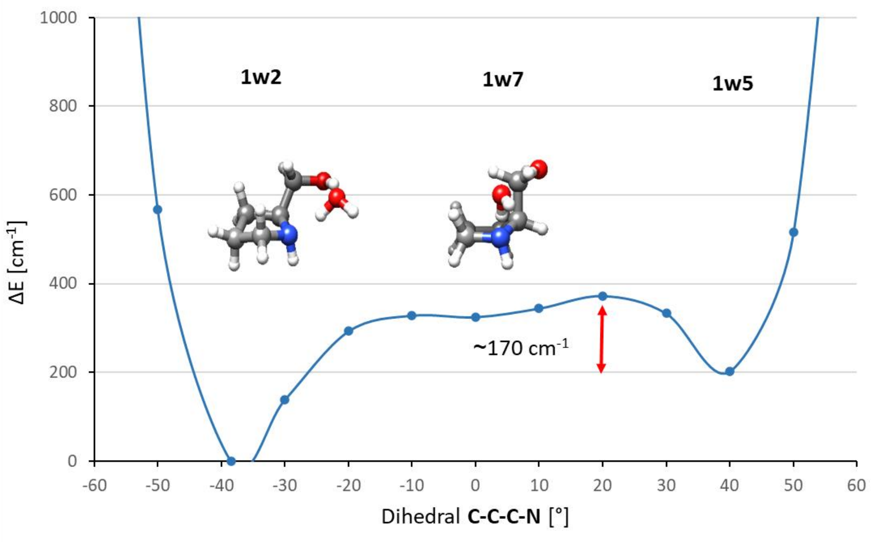

4.3. Prolinol-$(H_2O)_2$

Isomers **2w1**, **2w3** and **2w6** have very similar rotational and quadrupole coupling constants, and only differ in the orientation of the hydrogen atoms that are not involved in hydrogen bonding. Our identification of the observed species as **2w1** is supported by the low barriers for interconversion of **2w3** and **2w6** to **2w1** (see below), which rationalise their non-observation. **2w6** can convert to **2w1** through an almost barrierless pathway. **2w3** has a low barrier of about 165 cm^{-1} to **2w6**, which then rearranges to **2w1** virtually barrierless.

Figure S9. Interconversion barriers between the isomers of prolinol-$(H_2O)_2$ **2w6** ↔ **2w1** (top) and **2w3** ↔ **2w6** ↔ **2w1** (bottom) at the B3LYP-D3BJ/6-311++G(d,p) level of theory.

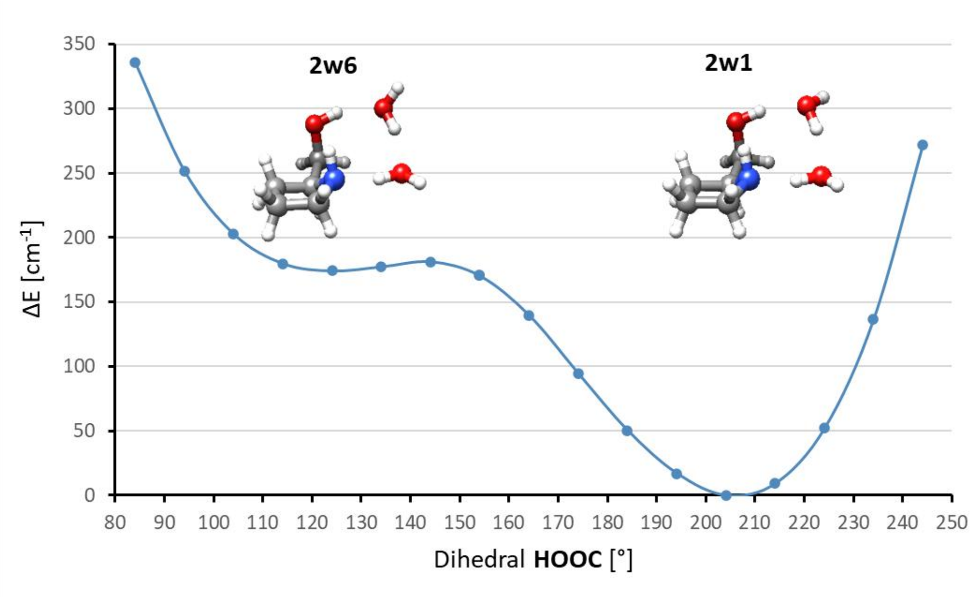

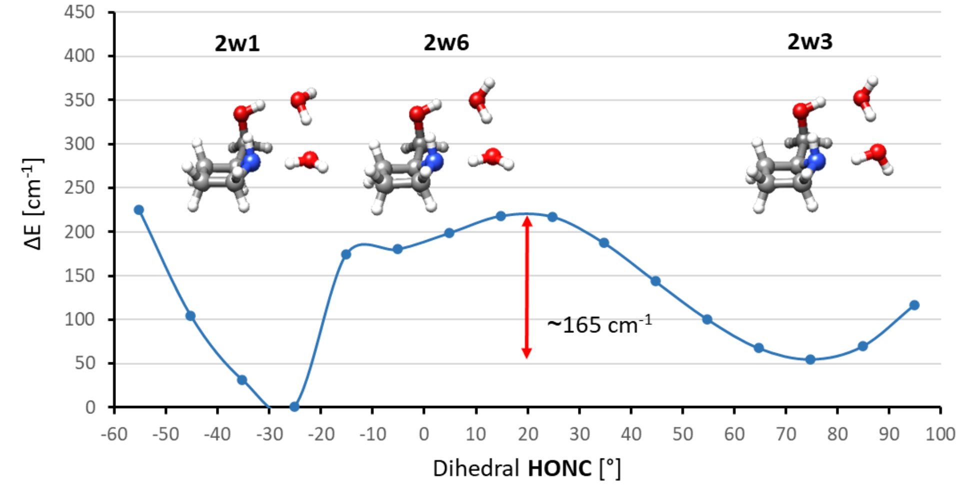

5. NBO calculations

5.1. Prolinol-H_2O

Table S11. Intermolecular stabilising energy contributions (≥0.42 kJ mol^{-1}) for **isomer 1w1** and **1w2** of prolinol-H_2O from Natural Bond Orbital (NBO) analysis at the B3LYP-D3BJ/6-311++G(d,p) level of theory.

**1w1**			**1w2**		
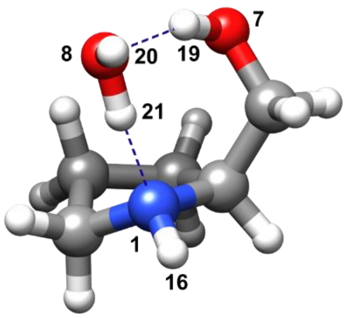			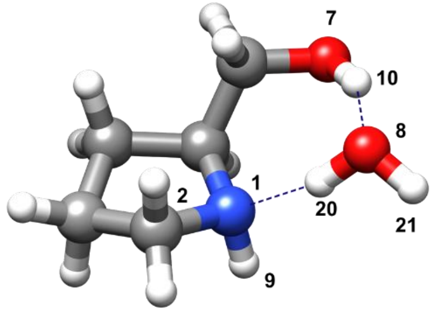		
**From prolinol to water**			**From prolinol to water**		
**Donor**	**Acceptor**	**kJ/mol**	**Donor**	**Acceptor**	**kJ/mol**
BD (1) N1 - C2	BD*(1) O8 - H21	0.46	BD (1) N1 - C2	BD*(1) O8 - H20	0.46
BD (1) N1 - H16	BD*(1) O8 - H21	0.79	BD (1) N1 - H9	BD*(1) O8 - H20	0.50
BD (1) O7 - H19	BD*(1) O8 - H20	0.75	BD (1) O7 - H10	BD*(1) O8 - H21	0.50
LP (1) N1	RY*(4) O8	0.75	LP (1) N1	RY*(2) O8	0.59
LP (1) N1	RY*(4) H21	1.13	LP (1) N1	RY*(3) O8	0.46
LP (1) N1	RY*(6) H21	0.42	LP (1) N1	RY*(4) O8	0.50
LP (1) N1	BD*(1) O8 - H21	88.07	LP (1) N1	RY*(4) H20	1.38
LP (2) O7	RY*(1) O8	0.50	LP (1) N1	RY*(6) H20	0.42
			LP (1) N1	BD*(1) O8 - H20	86.11
**From water to prolinol**			**From water to prolinol**		
**Donor**	**Acceptor**	**kJ/mol**	**Donor**	**Acceptor**	**kJ/mol**
BD (1) O8 - H20	RY*(1) N1	0.42	BD (1) O8 - H20	RY*(1) N1	0.63
BD (1) O8 - H20	RY*(1) H19	0.59	BD (1) O8 - H20	RY*(1) H10	0.50
BD (1) O8 - H21	RY*(1) N1	0.63	BD (1) O8 - H20	BD*(1) O7 - H10	1.76
BD (1) O8 - H21	RY*(1) H19	0.50	BD (1) O8 - H21	RY*(1) N1	0.42
BD (1) O8 - H21	BD*(1) O7 - H19	2.09	LP (2) O8	RY*(4) O7	0.42
LP (1) O8	BD*(1) O7 - H19	0.71	LP (2) O8	RY*(1) H10	0.42
LP (2) O8	RY*(1) H19	0.50	LP (2) O8	BD*(1) O7 - H10	41.42
LP (2) O8	RY*(5) H19	0.59			
LP (2) O8	BD*(1) O7 - H 19	43.97			

Table S12. Intermolecular stabilising energy contributions (≥0.42 kJ mol^{-1}) for **isomer 1w9** and **1w12** of prolinol-H_2O from Natural Bond Orbital (NBO) analysis at the B3LYP-D3BJ/6-311++G(d,p) level of theory.

**1w9**			**1w12**		
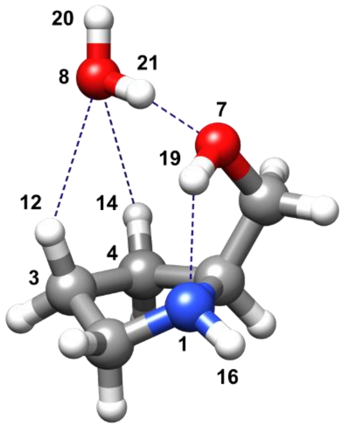			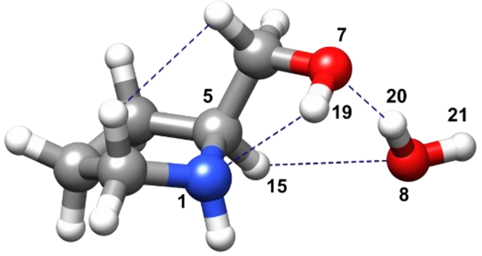		
**From prolinol to water**			**From prolinol to water**		
**Donor**	**Acceptor**	**kJ/mol**	**Donor**	**Acceptor**	**kJ/mol**
BD (1) C4 - H14	BD*(1) O8 - H20	0.50	BD (1) C5 - H15	RY*(1) O8	0.92
LP (1) O7	BD*(1) O8 - H21	9.75	BD (1) C5 - H15	BD*(1) O8 - H21	0.42
LP (2) O7	RY*(4) O8	0.84	LP (1) O7	BD*(1) O8 - H20	5.02
LP (2) O7	RY*(4) H21	0.50	LP (2) O7	RY*(5) O8	0.84
LP (2) O7	BD*(1) O8 - H21	42.80	LP (2) O7	RY*(4) H20	0.42
			LP (2) O7	BD*(1) O8 - H20	40.71
**From water to prolinol**			**From water to prolinol**		
**Donor**	**Acceptor**	**kJ/mol**	**Donor**	**Acceptor**	**kJ/mol**
BD (1) O8 - H20	RY*(1) H14	1.51	BD (1) O8 - H21	RY*(1) H15	1.30
LP (1) O8	BD*(1) C3 - H12	0.50	LP (1) O8	BD*(1) C5 - H15	0.54
LP (1) O8	BD*(1) C4 - H14	0.50	LP (2) O8	BD*(1) C5 - H15	1.34
LP (2) O8	BD*(1) C4 - H14	2.51			

5.2. Prolinol-$(H_2O)_2$

Table S13. Intermolecular stabilising energy contributions (≥0.42 kJ mol^{-1}) for isomers **2w1** and **2w5** of prolinol-$(H_2O)_2$ from Natural Bond Orbital (NBO) analysis at the B3LYP-D3BJ/6-311++G(d,p) level of theory.

**2w1**			**2w5**		
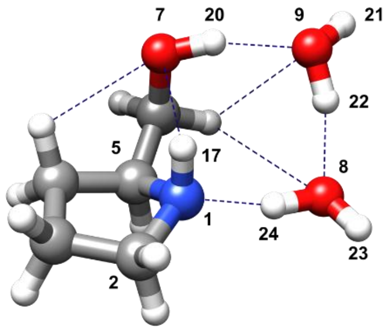			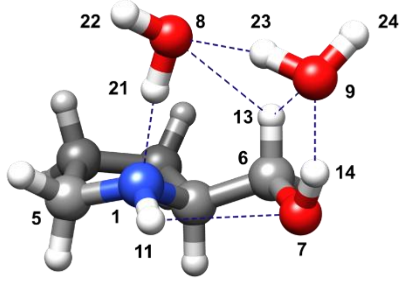		
**From prolinol to water1**			**From prolinol to water1**		
**Donor**	**Acceptor**	**kJ mol^{-1}**	**Donor**	**Acceptor**	**kJ mol^{-1}**
BD (1) N 1 - C 2	BD*(1) O 8 - H 24	0.50	BD (1) N 1 - C 5	BD* (1) O 8 - H 21	0.50
BD (1) N 1 - C 5	BD*(1) O 8 - H 24	0.71	BD (1) N 1 - H 11	BD* (1) O 8 - H 21	1.26
BD (1) N 1 - H 17	BD*(1) O 8 - H 24	1.38	BD (1) C 2 - H 15	BD* (1) O 8 - H 21	0.42
CR (1) N 1	BD*(1) O 8 - H 24	0.63	BD (1) C 6 - H 13	RY* (1) O 8	0.59
LP (1) N 1	RY*(3) O 8	0.63	CR (1) N 1	BD* (1) O 8 - H 21	0.59
LP (1) N 1	RY*(4) O 8	0.88	LP (1) N 1	RY* (3) O 8	0.42
LP (1) N 1	RY*(4) H 24	1.17	LP (1) N 1	RY* (4) O 8	0.96
LP (1) N 1	RY*(6) H 24	0.42	LP (1) N 1	RY* (4) H 21	0.88
LP (1) N 1	BD*(1) O 8 - H 24	106.86	LP (1) N 1	RY* (5) H 21	0.46
			LP (1) N 1	RY* (6) H 21	0.50
			LP (1) N 1	BD* (1) O 8 - H 21	103.93
**From prolinol to water2**			**From prolinol to water2**		
**Donor**	**Acceptor**	**kJ mol^{-1}**	**Donor**	**Acceptor**	**kJ mol^{-1}**
BD (1) O 7 - H 20	BD*(1) O 9 - H 21	0.54	BD (1) O 7 - H 14	BD*(1) O 9 - H 24	0.46
LP (2) O 7	RY*(1) O 9	0.71	LP (2) O 7	RY*(1) O 9	0.92
**From water1 to prolinol**			**From water1 to prolinol**		
**Donor**	**Acceptor**	**kJ mol^{-1}**	**Donor**	**Acceptor**	**kJ mol^{-1}**
BD (1) O 8 - H 24	RY*(2) N 1	0.46	BD (1) O 8 - H 21	RY*(1) N 1	0.46
BD (1) O 8 - H 24	RY*(6) N 1	0.42	BD (1) O 8 - H 22	RY*(1) H 13	0.54
BD (1) O 8 - H 24	BD*(1) N 1 - C 2	0.54	LP (1) O 8	BD*(1) C 6 - H 13	0.59
**From water1 to water2**			**From water1 to water2**		
**Donor**	**Acceptor**	**kJ mol^{-1}**	**Donor**	**Acceptor**	**kJ mol^{-1}**
BD (1) O 8 - H 23	RY*(1) H 22	0.46	BD (1) O 8 - H 21	BD*(1) O 9 - H 23	0.92
BD (1) O 8 - H 24	BD*(1) O 9 - H 22	1.09	LP (1) O 8	BD*(1) O 9 - H 23	0.67
LP (1) O 8	BD*(1) O 9 - H 22	0.67	LP (2) O 8	RY*(3) O 9	0.59
LP (2) O 8	RY*(5) O 9	0.71	LP (2) O 8	RY*(5) O 9	0.63
LP (2) O 8	RY*(6) H 22	0.59	LP (2) O 8	BD*(1) O 9 - H 23	67.91
LP (2) O 8	BD*(1) O 9 - H 22	67.82			
**From water2 to prolinol**			**From water2 to prolinol**		
**Donor**	**Acceptor**	**kJ mol^{-1}**	**Donor**	**Acceptor**	**kJ mol^{-1}**
LP (1) O 9	BD*(1) O 7 - H 20	1.09	BD (1) O 9 - H 23	BD*(1) O 7 - H 14	0.59
LP (2) O 9	RY*(1) H 20	0.67	LP (1) O 9	BD*(1) O 7 - H 14	1.13
LP (2) O 9	RY*(6) H 20	0.75	LP (2) O 9	RY*(1) H 14	0.42
LP (2) O 9	BD*(1) O 7 - H 20	54.52	LP (2) O 9	RY*(4) H 14	1.05
			LP (2) O 9	BD*(1) O 7 - H 14	55.56
**From water2 to water1**			**From water2 to water1**		
**Donor**	**Acceptor**	**kJ mol^{-1}**	**Donor**	**Acceptor**	**kJ mol^{-1}**
BD (1) O 9 - H 22	BD*(1) O 8 - H 23	0.79	BD (1) O 9 - H 23	BD*(1) O 8 - H 22	0.67

5.3. Prolinol-$(H_2O)_3$

Table S14. Intermolecular stabilising energy contributions (≥0.42 kJ mol^{-1}) for **isomer 3w1** of prolinol-$(H_2O)_3$ from Natural Bond Orbital (NBO) analysis at the B3LYP-D3BJ/6-311++G(d,p) level of theory.

**From prolinol to water1**		
**Donor**	**Acceptor**	**kJ/mol**
BD (1) N 1 - C 2	BD*(1) O 8 - H 23	1.09
BD (1) N 1 - C 5	BD*(1) O 8 - H 23	0.92
BD (1) N 1 - H 18	BD*(1) O 8 - H 23	1.46
CR (1) N 1	BD*(1) O 8 - H 23	0.92
LP (1) N 1	RY*(1) O 8	0.96
LP (1) N 1	RY*(8) O 8	0.59
LP (1) N 1	RY*(5) H 23	0.79
LP (1) N 1	RY*(6) H 23	1.97
LP (1) N 1	BD*(1) O 8 - H 23	127.90
**From prolinol to water3**		
**Donor**	**Acceptor**	**kJ/mol**
BD (1) N 1 - H 18	BD*(1) O 10 - H 27	0.71
BD (1) O 7 - H 21	BD*(1) O 10 - H 27	0.50
LP (2) O 7	RY*(1) O 10	0.46
**From water1 to prolinol**		
**Donor**	**Acceptor**	**kJ/mol**
BD (1) O 8 - H 23	RY*(1) N 1	0.84
**From water1 to water2**		
**Donor**	**Acceptor**	**kJ/mol**
BD (1) O 8 - H 22	RY*(1) H 25	0.42
BD (1) O 8 - H 23	BD*(1) O 9 - H 25	1.38
CR (1) O 8	BD*(1) O 9 - H 25	0.54
LP (1) O 8	BD*(1) O 9 - H 25	1.05
LP (2) O 8	RY*(2) O 9	0.67
LP (2) O 8	RY*(4) O 9	0.46
LP (2) O 8	RY*(5) O 9	0.50
LP (2) O 8	RY*(5) H 25	0.71
LP (2) O 8	BD*(1) O 9 - H 25	87.45
**From water2 to water1**		
**Donor**	**Acceptor**	**kJ/mol**
BD (1) O 9 - H 25	BD*(1) O 8 - H 22	0.75
**From water2 to water3**		
**Donor**	**Acceptor**	**kJ/mol**
BD (1) O 9 - H 25	BD*(1) O 10 - H 26	1.00
CR (1) O 9	BD*(1) O 10 - H 26	0.46
LP (1) O 9	BD*(1) O 10 - H 26	1.30
LP (2) O 9	RY*(1) O 10	0.59
LP (2) O 9	RY*(2) O 10	0.59
LP (2) O 9	RY*(5) O 10	0.54
LP (2) O 9	RY*(5) H 26	0.79
LP (2) O 9	BD*(1) O 10 - H 26	82.05
**From water3 to prolinol**		
**Donor**	**Acceptor**	**kJ/mol**
BD (1) O 10 - H 27	RY*(1) H 18	1.30
LP (1) O 10	RY*(1) H 18	0.75
LP (1) O 10	BD*(1) N 1 - H 18	12.72
LP (1) O 10	BD*(1) O 7 - H 21	0.42
LP (2) O 10	RY*(4) H 21	0.63
LP (2) O 10	BD*(1) N 1 - H 18	5.69
LP (2) O 10	BD*(1) O 7 - H 21	46.44
**From water3 to water2**		
**Donor**	**Acceptor**	**kJ/mol**
BD (1) O 10 - H 26	BD*(1) O 9 - H 24	0.59

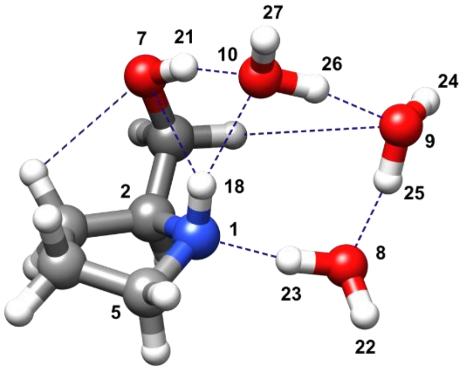

6. SAPT calculations

Table S15. Binding energy decomposition (SAPT2+/aug-cc-pVDZ) in kJ mol^{-1} for the observed prolinol-water complexes on B3LYP-D3BJ/6-311++G(d,p) geometries.

**Complex**	ΔE_{el}	ΔE_{ex}	ΔE_{ind}	ΔE_{dis}	ΔE_{total}	$\%\Delta E_{el}$[a]	$\%\Delta E_{ind}$[a]	% ΔE_{dis}[a]
**1w1**	-118.5	153.2	-48.9	-37.3	-51.6	58	24	18
**1w2**	-114.0	144.4	-46.2	-34.8	-50.5	58	24	18
**1w9**	-57.3	65.9	-20.3	-21.2	-33.0	58	21	21
**1w12**	-54.1	59.4	-18.3	-18.5	-31.4	60	20	20
**2w1**	-147.2	170.0	-63.8	-42.5	-83.4	58	25	17
**2w5**	-144.5	172.6	-63.5	-44.2	-79.7	57	25	18
**3w1**	-169.4	198.1	-76.5	-51.1	-98.8	57	26	17

[a] Percentage contribution of electrostatic, induction, or dispersion term with respect to the total attractive forces, calculated for each isomer as $\frac{\Delta E_{\mathrm{value}}}{\Delta E_{\mathrm{el}}+\Delta E_{\mathrm{ind}}+\Delta E_{\mathrm{dis}}} \times 100$

7. NCI and RDG plots

Plots of the reduced density gradient (RDG) versus sign(λ_2)ρ for the observed conformers of prolinol, prolinol-H_2O, prolinol-$(H_2O)_2$, and prolinol-$(H_2O)_3$.

Prolinol I	Prolinol II		
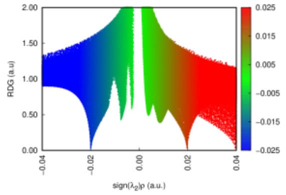	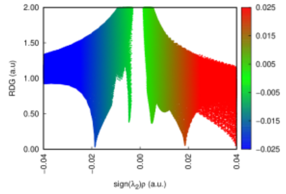		
1w1	1w2	1w9	1w12
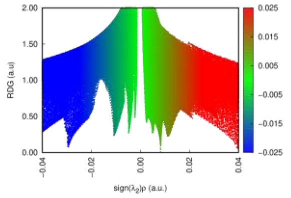	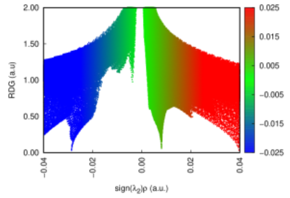	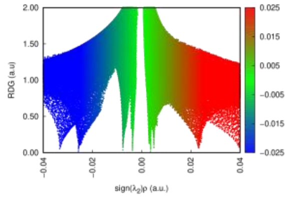	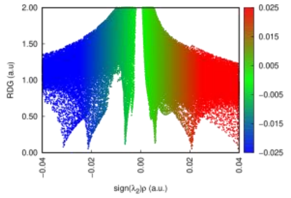
2w1	2w5	3w1	
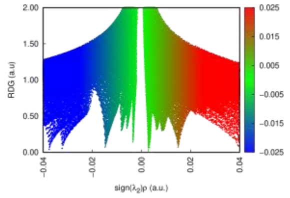	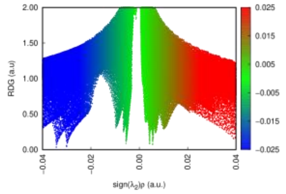	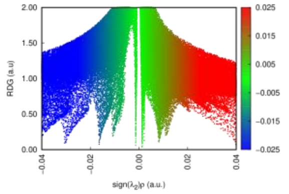	

8. Frequencies of measured transitions and their residuals

8.1. Prolinol

Table S16. Measured frequencies (ν_{obs}) and residuals ($\nu_{obs} - \nu_{cal}$) of the rotational transitions of the parent species of prolinol **I**.

J'	K'_{-1}	K'_{+1}	F	←	J''	K''_{-1}	K''_{+1}	F''	ν_{obs} /MHz	ν_{obs}- ν_{calc}/MHz
3	1	3	2	←	3	0	3	2	2066.3554	0.0024
3	1	3	4	←	3	0	3	4	2066.5943	0.0014
3	1	3	3	←	3	0	3	3	2067.2793	0.0013
2	1	2	1	←	2	0	2	2	2288.0373	0.0003
2	1	2	3	←	2	0	2	2	2288.6201	-0.0040
2	1	2	1	←	2	0	2	1	2288.6924	0.0029
2	1	2	3	←	2	0	2	3	2289.0455	0.0020
2	1	2	2	←	2	0	2	2	2289.6814	0.0003
2	1	2	2	←	2	0	2	3	2290.0986	-0.0019
2	1	2	2	←	2	0	2	1	2290.3340	0.0005
1	1	1	0	←	1	0	1	1	2446.3903	-0.0007
1	1	1	2	←	1	0	1	1	2447.3641	0.0008
1	1	1	2	←	1	0	1	2	2447.7026	0.0012
1	1	1	1	←	1	0	1	1	2448.0082	-0.0032
1	1	1	1	←	1	0	1	2	2448.3474	-0.0022
1	1	1	1	←	1	0	1	0	2448.8570	0.0001
1	1	0	1	←	1	0	1	1	2613.4763	-0.0058
1	1	0	1	←	1	0	1	2	2613.8206	0.0003
1	1	0	1	←	1	0	1	0	2614.3275	-0.0001
1	1	0	2	←	1	0	1	1	2614.4701	0.0016
1	1	0	2	←	1	0	1	2	2614.8063	-0.0005
1	1	0	0	←	1	0	1	1	2615.9478	-0.0005
2	1	1	2	←	2	0	2	2	2788.8240	-0.0011
2	1	1	2	←	2	0	2	3	2789.2407	-0.0039
2	1	1	2	←	2	0	2	1	2789.4924	-0.0062
2	1	1	3	←	2	0	2	2	2789.4924	-0.0062
2	1	1	3	←	2	0	2	3	2789.9390	-0.0001
2	1	1	1	←	2	0	2	1	2790.5586	0.0005
3	1	2	3	←	3	0	3	3	3066.6976	-0.0004
3	1	2	3	←	3	0	3	4	3067.2193	-0.0014
3	1	2	4	←	3	0	3	4	3067.8282	0.0004
3	1	2	2	←	3	0	3	2	3068.2246	0.0014
4	1	3	4	←	4	0	4	4	3464.3792	-0.0028
4	1	3	5	←	4	0	4	4	3464.9225	-0.0109
4	1	3	4	←	4	0	4	3	3465.1928	-0.0074
4	1	3	5	←	4	0	4	5	3465.5817	-0.0026
4	1	3	3	←	4	0	4	3	3465.8914	-0.0020
3	1	3	4	←	2	2	0	3	3747.4135	0.0025
3	1	3	3	←	2	2	0	2	3749.0318	-0.0064
3	1	3	2	←	2	2	1	1	3755.0391	-0.0011
3	1	3	4	←	2	2	1	3	3755.6561	-0.0083
3	1	3	3	←	2	2	1	3	3756.8794	0.0072
3	1	3	3	←	2	2	1	2	3757.2337	-0.0011
1	0	1	0	←	0	0	0	1	3951.1434	-0.0015
1	0	1	2	←	0	0	0	1	3951.6517	-0.0005
1	0	1	1	←	0	0	0	1	3951.9898	-0.0006
5	1	4	5	←	5	0	5	5	4001.8370	0.0005
5	1	4	6	←	5	0	5	6	4003.1333	-0.0005
5	1	4	4	←	5	0	5	4	4003.3989	0.0007
6	1	5	6	←	6	0	6	6	4697.8836	-0.0018
6	1	5	7	←	6	0	6	7	4699.2788	-0.0035
6	1	5	5	←	6	0	6	5	4699.5108	-0.0074
3	1	2	3	←	2	2	0	3	4748.0257	-0.0131
3	1	2	3	←	2	2	0	2	4748.4498	-0.0084
3	1	2	2	←	2	2	0	1	4748.6385	-0.0009
3	1	2	4	←	2	2	0	3	4748.6385	-0.0009
3	1	2	2	←	2	2	0	2	4749.2917	0.0139

3	1	2	3	←	2	2	1	2	4756.6600	0.0052
3	1	2	4	←	2	2	1	3	4756.9086	0.0057
3	1	2	2	←	2	2	1	1	4756.9086	0.0057
2	0	2	1	←	1	1	0	0	5278.5835	-0.0014
2	0	2	1	←	1	1	0	2	5280.0705	0.0059
2	0	2	3	←	1	1	0	2	5280.2964	-0.0013
2	0	2	2	←	1	1	0	2	5280.7156	-0.0015
2	0	2	1	←	1	1	0	1	5281.0506	-0.0005
2	0	2	2	←	1	1	0	1	5281.7018	-0.0016
7	2	6	6	←	7	1	6	6	5391.5969	-0.0003
7	2	6	7	←	7	1	6	6	5392.0691	-0.0026
7	2	6	7	←	7	1	6	7	5392.4958	0.0089
2	0	2	1	←	1	1	1	1	5446.5249	0.0032
2	0	2	2	←	1	1	1	1	5447.1757	0.0015
2	0	2	3	←	1	1	1	2	5447.3996	-0.0033
2	0	2	2	←	1	1	1	2	5447.8193	-0.0030
2	0	2	1	←	1	1	1	0	5448.1406	-0.0016
6	2	5	5	←	6	1	5	5	5914.6794	0.0023
6	2	5	7	←	6	1	5	7	5914.8113	0.0017
6	2	5	6	←	6	1	5	6	5915.5963	0.0022
5	2	4	4	←	5	1	4	4	6378.8148	-0.0060
5	2	4	6	←	5	1	4	6	6378.9794	0.0019
5	2	4	6	←	5	1	4	5	6379.4777	0.0027
5	2	4	5	←	5	1	4	5	6379.7477	0.0016
1	1	1	0	←	0	0	0	1	6398.3797	-0.0017
1	1	1	2	←	0	0	0	1	6399.3527	-0.0010
1	1	1	1	←	0	0	0	1	6400.0023	0.0005
1	1	0	1	←	0	0	0	1	6565.4716	-0.0010
1	1	0	2	←	0	0	0	1	6566.4577	-0.0012
1	1	0	0	←	0	0	0	1	6567.9378	-0.0009
4	2	3	3	←	4	1	3	3	6775.2738	0.0041
4	2	3	5	←	4	1	3	5	6775.4579	0.0032
4	2	3	4	←	4	1	3	4	6776.1793	0.0054
4	2	2	3	←	4	1	3	3	6897.7101	0.0064
4	2	2	5	←	4	1	3	5	6897.8236	0.0035
4	2	2	4	←	4	1	3	4	6898.2799	0.0071
5	2	4	6	←	4	3	2	5	7056.8131	-0.0024
5	2	4	5	←	4	3	2	4	7057.2732	0.0073
3	2	2	2	←	3	1	2	2	7097.9630	0.0019
3	2	2	4	←	3	1	2	4	7098.1763	0.0028
3	2	2	3	←	3	1	2	3	7098.7825	0.0019
4	1	4	3	←	3	2	1	2	7316.1410	0.0003
4	1	4	5	←	3	2	1	4	7316.5347	0.0081
4	1	4	4	←	3	2	1	3	7317.9763	0.0025
2	2	1	2	←	2	1	1	1	7342.3582	0.0010
2	2	1	2	←	2	1	1	3	7342.7437	0.0004
2	2	1	1	←	2	1	1	1	7342.9190	-0.0022
2	2	1	3	←	2	1	1	3	7343.1083	0.0025
2	2	1	2	←	2	1	1	2	7343.4369	-0.0009
2	2	1	3	←	2	1	1	2	7343.8027	0.0025
2	2	1	1	←	2	1	1	2	7343.9989	-0.0029
2	2	0	2	←	2	1	1	1	7350.5450	-0.0087
2	2	0	2	←	2	1	1	3	7350.9297	-0.0101
2	2	0	1	←	2	1	1	1	7351.2022	-0.0041
2	2	0	3	←	2	1	1	3	7351.3635	0.0043
2	2	0	2	←	2	1	1	2	7351.6349	0.0006
2	2	0	3	←	2	1	1	2	7352.0442	-0.0094
2	2	0	1	←	2	1	1	2	7352.2897	0.0029
4	1	4	3	←	3	2	2	2	7357.2965	-0.0042
4	1	4	5	←	3	2	2	4	7357.6314	-0.0037
4	1	4	4	←	3	2	2	3	7358.9409	0.0054
2	1	2	1	←	1	1	1	1	7735.2109	-0.0003
2	1	2	1	←	1	1	1	2	7735.8566	-0.0027
2	1	2	3	←	1	1	1	2	7736.4456	-0.0009
2	1	2	2	←	1	1	1	1	7736.8629	0.0076

2	1	2	2	←	1	1	1	2	7737.5020	-0.0014
2	2	1	2	←	2	1	2	2	7842.5812	-0.0007
2	2	1	3	←	2	1	2	2	7842.9359	-0.0084
2	2	1	1	←	2	1	2	2	7843.1495	0.0036
2	2	1	2	←	2	1	2	3	7843.6488	0.0101
2	2	1	3	←	2	1	2	3	7844.0026	0.0014
2	2	1	2	←	2	1	2	1	7844.2220	-0.0039
2	2	1	1	←	2	1	2	1	7844.7956	0.0057
2	2	0	2	←	2	1	2	2	7850.7776	-0.0008
2	2	0	3	←	2	1	2	2	7851.2001	0.0023
2	2	0	1	←	2	1	2	2	7851.4292	-0.0018
2	2	0	2	←	2	1	2	3	7851.8392	0.0039
2	2	0	3	←	2	1	2	3	7852.2552	0.0005
2	2	0	2	←	2	1	2	1	7852.4224	-0.0001
2	2	0	1	←	2	1	2	1	7853.0737	-0.0013
2	0	2	1	←	1	0	1	1	7894.5315	-0.0017
2	0	2	1	←	1	0	1	2	7894.8657	-0.0056
2	0	2	3	←	1	0	1	2	7895.1014	-0.0030
2	0	2	2	←	1	0	1	1	7895.1879	0.0024
2	0	2	1	←	1	0	1	0	7895.3779	-0.0008
2	0	2	1	←	1	0	1	0	7895.3780	-0.0007
2	0	2	2	←	1	0	1	2	7895.5222	-0.0015
2	0	2	2	←	1	0	1	2	7895.5222	-0.0015
2	1	1	1	←	1	1	0	0	8069.1425	-0.0005
2	1	1	2	←	1	1	0	2	8069.5417	-0.0005
2	1	1	3	←	1	1	0	2	8070.2365	-0.0002
2	1	1	2	←	1	1	0	1	8070.5274	-0.0013
2	1	1	1	←	1	1	0	1	8071.6081	-0.0011
3	2	2	3	←	3	1	3	3	8098.2056	0.0050
3	2	2	4	←	3	1	3	4	8099.4098	0.0014
3	2	2	2	←	3	1	3	2	8099.8386	0.0073
3	2	2	3	←	3	1	3	2	8099.8386	0.0073
3	2	1	3	←	3	1	3	3	8139.1619	-0.0004
3	2	1	4	←	3	1	3	3	8139.3134	0.0043
3	2	1	2	←	3	1	3	3	8139.3826	0.0220
3	2	1	3	←	3	1	3	4	8140.3600	-0.0102
3	2	1	4	←	3	1	3	4	8140.5195	0.0025
3	2	1	3	←	3	1	3	2	8140.7926	-0.0002
3	2	1	2	←	3	1	3	2	8140.9931	0.0018
4	2	3	3	←	4	1	4	4	8441.2621	-0.0070
4	2	3	4	←	4	1	4	4	8441.4823	0.0022
4	2	3	5	←	4	1	4	5	8442.6106	-0.0020
4	2	3	3	←	4	1	4	3	8442.8992	-0.0046
4	2	2	4	←	4	1	4	4	8563.5877	0.0087
4	2	2	5	←	4	1	4	5	8564.9838	0.0058
4	2	2	3	←	4	1	4	3	8565.3406	0.0028
4	1	3	4	←	3	2	1	3	8983.2944	0.0144
4	1	3	5	←	3	2	1	4	8983.7062	-0.0116
4	1	3	3	←	3	2	1	2	8983.7062	-0.0116
4	1	3	4	←	3	2	2	3	9024.2511	0.0095
4	1	3	5	←	3	2	2	4	9024.7971	0.0040
4	1	3	3	←	3	2	2	2	9024.9408	0.0060
3	0	3	2	←	2	1	1	1	9031.6103	0.0019
3	0	3	4	←	2	1	1	3	9032.1795	0.0023
3	0	3	2	←	2	1	1	2	9032.7032	0.0087
3	0	3	3	←	2	1	1	3	9032.7032	0.0087
3	0	3	3	←	2	1	1	2	9033.3968	0.0023
5	2	3	5	←	5	1	5	5	9155.0919	0.0046
5	2	3	6	←	5	1	5	6	9156.6009	0.0027
5	2	3	4	←	5	1	5	4	9156.9133	0.0071
6	2	5	5	←	6	1	6	6	9396.6544	-0.0069
6	2	5	6	←	6	1	6	6	9397.0689	0.0006
6	2	5	7	←	6	1	6	7	9398.1520	-0.0050
6	2	5	5	←	6	1	6	5	9398.3328	-0.0080
3	0	3	2	←	2	1	2	2	9531.8389	0.0059

3	0	3	3	←	2	1	2	2	9532.5379	-0.0007
3	0	3	4	←	2	1	2	3	9533.0717	-0.0010
3	0	3	2	←	2	1	2	1	9533.4746	-0.0025
2	1	2	1	←	1	0	1	1	10183.2222	-0.0004
2	1	2	1	←	1	0	1	2	10183.5631	0.0023
2	1	2	3	←	1	0	1	2	10184.1378	-0.0101
2	1	2	2	←	1	0	1	1	10184.8648	-0.0018
2	1	2	2	←	1	0	1	2	10185.2052	0.0004
4	2	3	4	←	4	0	4	4	10240.5713	0.0154
4	2	3	5	←	4	0	4	5	10241.0403	0.0015
4	2	3	3	←	4	0	4	3	10241.1829	0.0198
2	1	1	2	←	1	0	1	1	10684.0105	-0.0002
2	1	1	2	←	1	0	1	2	10684.3479	-0.0010
2	1	1	3	←	1	0	1	2	10685.0386	-0.0048
2	1	1	1	←	1	0	1	2	10685.4314	0.0019
2	1	1	1	←	1	0	1	0	10685.9379	0.0012
5	1	5	4	←	4	2	2	3	10741.9179	-0.0314
5	1	5	6	←	4	2	2	5	10742.2547	0.0004
5	1	5	5	←	4	2	2	4	10743.7352	0.0095
5	1	5	4	←	4	2	3	3	10864.3966	0.0135
5	1	5	6	←	4	2	3	5	10864.6327	0.0129
5	1	5	5	←	4	2	3	4	10865.8350	0.0104
5	1	5	5	←	4	2	3	5	10865.9916	-0.0009
6	2	5	7	←	5	3	3	6	10944.9556	0.0152
6	2	5	6	←	5	3	3	5	10945.3164	-0.0001
7	2	5	7	←	7	1	7	7	10966.8398	0.0117
7	2	5	6	←	7	1	7	6	10968.8994	0.0064
6	2	4	6	←	5	3	2	5	11487.5469	-0.0107
6	2	4	7	←	5	3	2	6	11487.7370	-0.0057
6	2	4	5	←	5	3	2	4	11487.7370	-0.0057
6	2	4	6	←	5	3	3	5	11494.6474	-0.0053
6	2	4	7	←	5	3	3	6	11494.8487	-0.0062
6	2	4	5	←	5	3	3	4	11494.8487	-0.0062
3	1	3	2	←	2	1	2	2	11598.1871	0.0011
3	1	3	2	←	2	1	2	3	11599.2390	-0.0039
3	1	3	4	←	2	1	2	3	11599.6753	0.0097
3	1	3	3	←	2	1	2	2	11599.8164	-0.0056
3	1	3	2	←	2	1	2	1	11599.8164	-0.0056
3	1	3	3	←	2	1	2	3	11600.8779	0.0044
3	0	3	2	←	2	0	2	2	11821.5154	0.0013
3	0	3	4	←	2	0	2	3	11822.1046	-0.0117
3	0	3	3	←	2	0	2	2	11822.2298	0.0101
3	0	3	3	←	2	0	2	3	11822.6427	0.0037
3	2	2	2	←	2	2	1	1	11854.8743	0.0028
3	2	2	4	←	2	2	1	3	11855.0771	0.0043
3	2	2	3	←	2	2	1	3	11855.0772	0.0043
3	2	2	3	←	2	2	1	2	11855.4406	0.0053
3	2	1	2	←	2	2	0	1	11887.7483	0.0019
3	2	1	4	←	2	2	0	3	11887.9315	0.0036
3	2	1	3	←	2	2	0	2	11888.2061	0.0057
3	2	1	2	←	2	2	0	2	11888.3939	-0.0049
3	1	2	3	←	2	1	1	3	12099.3988	0.0008
3	1	2	2	←	2	1	1	1	12099.8425	0.0109
3	1	2	4	←	2	1	1	3	12100.0341	-0.0067
3	1	2	3	←	2	1	1	2	12100.0341	-0.0067
3	1	2	2	←	2	1	1	2	12100.9134	0.0012
5	3	3	6	←	5	2	3	6	12445.9249	0.0069
5	3	3	5	←	5	2	3	5	12445.9989	-0.0295
4	3	2	4	←	4	2	2	4	12560.5769	0.0025
4	3	2	5	←	4	2	2	5	12560.5769	0.0025
4	3	2	3	←	4	2	2	3	12560.5769	0.0025
4	3	1	4	←	4	2	2	4	12562.3213	-0.0189
4	3	1	5	←	4	2	2	5	12562.3213	-0.0189
3	3	1	3	←	3	2	1	3	12619.1001	0.0000
3	3	1	4	←	3	2	1	4	12619.4239	-0.0169

3	3	1	2	←	3	2	1	2	12619.5558	-0.0042
4	0	4	3	←	3	1	2	2	12657.0098	0.0074
4	0	4	5	←	3	1	2	4	12657.3853	0.0030
4	0	4	3	←	3	1	2	3	12657.8257	0.0037
4	0	4	4	←	3	1	2	3	12658.6448	0.0046
3	3	1	3	←	3	2	2	3	12660.0643	0.0025
3	3	0	3	←	3	2	2	3	12660.3198	0.0043
3	3	1	4	←	3	2	2	4	12660.5616	0.0123
3	3	0	4	←	3	2	2	4	12660.8015	-0.0030
3	3	0	2	←	3	2	2	2	12660.9651	-0.0105
4	3	2	4	←	4	2	3	4	12682.6112	-0.0018
4	3	2	5	←	4	2	3	5	12682.9918	-0.0028
4	3	2	3	←	4	2	3	3	12682.9918	-0.0028
4	3	1	4	←	4	2	3	4	12684.3917	0.0015
4	3	1	5	←	4	2	3	5	12684.7793	0.0013
4	3	1	3	←	4	2	3	3	12684.7793	0.0013
5	3	3	5	←	5	2	4	5	12727.0593	-0.0020
5	3	3	5	←	5	2	4	6	12727.3480	0.0157
5	3	3	4	←	5	2	4	4	12727.4455	0.0244
5	3	2	5	←	5	2	4	5	12734.1622	0.0058
5	3	2	6	←	5	2	4	6	12734.4719	0.0004
5	3	2	5	←	5	2	4	4	12734.4719	0.0004
7	3	4	7	←	7	2	6	7	12973.3868	-0.0086
7	3	4	6	←	7	2	6	6	12973.8281	0.0035
5	1	4	5	←	4	2	2	4	13238.0226	-0.0114
5	1	4	6	←	4	2	2	5	13238.4335	0.0006
5	1	4	5	←	4	2	3	4	13360.1395	0.0066
5	1	4	6	←	4	2	3	5	13360.8066	0.0083
4	0	4	3	←	3	1	3	3	13657.2543	0.0123
4	0	4	4	←	3	1	3	3	13658.0635	0.0033
4	0	4	5	←	3	1	3	4	13658.6158	-0.0015
4	0	4	3	←	3	1	3	2	13658.8767	0.0041
4	0	4	4	←	3	1	3	4	13659.2754	0.0072
3	1	3	2	←	2	0	2	2	13887.8642	-0.0028
3	1	3	2	←	2	0	2	3	13888.2827	-0.0038
3	1	3	2	←	2	0	2	1	13888.5213	0.0019
3	1	3	4	←	2	0	2	3	13888.7115	0.0023
3	1	3	3	←	2	0	2	2	13889.5004	0.0028
3	1	3	3	←	2	0	2	3	13889.9194	0.0024
3	1	2	3	←	2	0	2	2	14888.9189	0.0013
3	1	2	3	←	2	0	2	3	14889.3379	0.0009
3	1	2	2	←	2	0	2	2	14889.7418	0.0045
3	1	2	4	←	2	0	2	3	14889.9467	0.0026
3	1	2	2	←	2	0	2	3	14890.1567	0.0000
3	1	2	2	←	2	0	2	1	14890.3898	0.0001
2	2	1	1	←	1	1	0	0	15412.0608	-0.0034
2	2	1	2	←	1	1	0	2	15412.9807	0.0008
2	2	1	3	←	1	1	0	2	15413.3419	-0.0005
2	2	1	2	←	1	1	0	1	15413.9634	-0.0030
2	2	1	1	←	1	1	0	1	15414.5285	-0.0019
2	2	0	1	←	1	1	0	0	15420.3453	-0.0039
2	2	0	2	←	1	1	0	2	15421.1760	-0.0004
2	2	0	3	←	1	1	0	2	15421.5923	-0.0035
2	2	0	1	←	1	1	0	2	15421.8331	0.0041
2	2	0	2	←	1	1	0	1	15422.1591	-0.0038
2	2	0	1	←	1	1	0	1	15422.8145	-0.0009
4	1	4	3	←	3	1	3	3	15455.5010	-0.0003
4	1	4	3	←	3	1	3	2	15457.1081	0.0124
4	1	4	4	←	3	1	3	3	15457.1081	0.0124
4	1	4	5	←	3	1	3	4	15457.1081	0.0124
4	1	4	4	←	3	1	3	4	15458.3280	-0.0159
2	2	1	2	←	1	1	1	1	15579.4323	-0.0048
2	2	1	1	←	1	1	1	1	15580.0472	0.0041
2	2	1	2	←	1	1	1	2	15580.0472	0.0041
2	2	1	3	←	1	1	1	2	15580.4413	-0.0064

2	2	1	1	←	1	1	1	0	15581.6165	-0.0050
2	2	0	2	←	1	1	1	1	15587.6335	-0.0002
2	2	0	2	←	1	1	1	2	15588.2849	0.0009
2	2	0	1	←	1	1	1	1	15588.2849	0.0009
2	2	0	3	←	1	1	1	2	15588.7001	-0.0011
2	2	0	1	←	1	1	1	0	15589.9038	-0.0028
4	0	4	3	←	3	0	3	3	15724.5210	0.0009
4	0	4	5	←	3	0	3	4	15725.2126	-0.0033
4	0	4	3	←	3	0	3	2	15725.2126	-0.0033
4	0	4	4	←	3	0	3	4	15725.8552	-0.0058
4	2	3	5	←	3	2	2	4	15800.2535	0.0058
4	2	3	4	←	3	2	2	3	15800.3974	-0.0182
4	3	2	4	←	3	3	1	4	15822.5150	-0.0148
4	3	2	3	←	3	3	1	2	15822.5150	-0.0148
4	3	2	5	←	3	3	1	4	15822.6371	-0.0216
4	3	2	4	←	3	3	1	3	15822.9613	-0.0055
4	3	1	5	←	3	3	0	4	15824.1606	-0.0258
4	3	1	4	←	3	3	0	3	15824.4823	-0.0080
4	2	2	3	←	3	2	1	2	15881.4816	-0.0133
4	2	2	5	←	3	2	1	4	15881.4816	-0.0133
4	1	3	4	←	3	1	2	4	16122.4293	0.0141
4	1	3	3	←	3	1	2	2	16122.9317	-0.0087
4	1	3	5	←	3	1	2	4	16122.9317	-0.0087
4	1	3	3	←	3	1	2	3	16123.7280	0.0125
5	0	5	6	←	4	1	3	5	16133.1335	0.0144
5	0	5	5	←	4	1	3	4	16134.4607	-0.0096
4	1	4	3	←	3	0	3	2	17523.4681	-0.0168
4	1	4	5	←	3	0	3	4	17523.6557	0.0193
4	1	4	4	←	3	0	3	3	17524.4021	-0.0120

Table S17. Measured frequencies (ν_{obs}) and residuals ($\nu_{obs} - \nu_{cal}$) of the rotational transitions of the $^{13}C_2$ isotopic species of prolinol I.

J'	*K'*$_{-1}$	*K'*$_{+1}$	*F*	←	*J"*	*K"*$_{-1}$	*K"*$_{+1}$	*F"*	ν_{obs} /MHz	ν_{obs}- ν_{calc}/MHz
1	0	1	0	←	0	0	0	1	3899.6695	-0.0106
1	0	1	2	←	0	0	0	1	3900.1961	-0.0032
1	0	1	1	←	0	0	0	1	3900.5406	-0.0048
1	1	1	2	←	0	0	0	1	6353.6337	-0.0144
1	1	0	1	←	0	0	0	1	6518.7412	-0.0044
1	1	0	2	←	0	0	0	1	6519.7399	0.0030
1	1	0	0	←	0	0	0	1	6521.2357	0.0117
2	1	2	1	←	1	1	1	1	7633.3196	-0.0036
2	1	2	3	←	1	1	1	2	7634.5556	-0.0027
2	1	2	2	←	1	1	1	1	7634.9815	0.0062
2	1	2	2	←	1	1	1	2	7635.6157	-0.0047
2	0	2	1	←	1	0	1	1	7791.7340	0.0001
2	0	2	3	←	1	0	1	2	7792.3321	-0.0138
2	0	2	2	←	1	0	1	1	7792.3321	-0.0138
2	0	2	1	←	1	0	1	0	7792.5921	-0.0071
2	0	2	2	←	1	0	1	2	7792.7445	-0.0005
2	1	1	1	←	1	1	0	0	7965.2159	0.0036
2	1	1	2	←	1	1	0	2	7965.6269	0.0029
2	1	1	3	←	1	1	0	2	7966.3167	0.0015
2	1	1	2	←	1	1	0	1	7966.6142	-0.0011
2	1	1	1	←	1	1	0	1	7967.6906	-0.0001
2	1	2	1	←	1	0	1	1	10087.0674	-0.0035
2	1	2	1	←	1	0	1	0	10088.0015	0.0080
2	1	2	3	←	1	0	1	2	10088.0015	0.0080
2	1	2	2	←	1	0	1	1	10088.7211	-0.0020
2	1	2	2	←	1	0	1	2	10089.0572	-0.0120
2	1	1	2	←	1	0	1	1	10584.8234	0.0079
2	1	1	2	←	1	0	1	2	10585.1608	-0.0008
2	1	1	3	←	1	0	1	2	10585.8752	0.0166
2	1	1	1	←	1	0	1	1	10585.8752	0.0166
2	1	1	1	←	1	0	1	0	10586.7607	0.0044
3	1	3	2	←	2	1	2	2	11445.4222	0.0041
3	1	3	4	←	2	1	2	3	11446.8957	-0.0098
3	1	3	3	←	2	1	2	2	11447.0593	-0.0041
3	1	3	2	←	2	1	2	1	11447.0593	-0.0041
3	1	3	3	←	2	1	2	3	11448.1137	-0.0072
3	0	3	2	←	2	0	2	2	11667.6149	0.0032
3	0	3	4	←	2	0	2	3	11668.2176	-0.0071
3	0	3	3	←	2	0	2	2	11668.3288	0.0011
3	0	3	3	←	2	0	2	3	11668.7558	0.0007
3	2	2	2	←	2	2	1	1	11700.5120	0.0020
3	2	2	4	←	2	2	1	3	11700.7146	-0.0015
3	2	2	3	←	2	2	1	2	11701.0822	-0.0049
3	2	2	2	←	2	2	1	2	11701.0822	-0.0050
3	2	1	2	←	2	2	0	1	11732.9131	-0.0081
3	2	1	4	←	2	2	0	3	11733.1033	-0.0044
3	2	1	3	←	2	2	0	2	11733.3878	-0.0015
3	2	1	2	←	2	2	0	2	11733.5695	-0.0168
3	1	2	3	←	2	1	1	3	11943.6088	0.0099
3	1	2	2	←	2	1	1	1	11944.0321	0.0051
3	1	2	4	←	2	1	1	3	11944.2311	-0.0060
3	1	2	3	←	2	1	1	2	11944.2311	-0.0060
3	1	2	2	←	2	1	1	2	11945.1122	0.0098
4	0	4	3	←	3	1	2	2	12450.6529	0.0071
4	0	4	5	←	3	1	2	4	12451.0402	0.0146
4	0	4	4	←	3	1	2	3	12452.2964	0.0111
3	1	3	2	←	2	0	2	2	13741.7372	-0.0051
3	1	3	2	←	2	0	2	1	13742.4108	0.0034
3	1	3	4	←	2	0	2	3	13742.5999	0.0047
3	1	3	3	←	2	0	2	2	13743.3783	-0.0047
3	1	2	4	←	2	0	2	3	14737.7381	0.0023

3	1	2	2	←	2	0	2	1	14738.1850	0.0010
2	2	1	3	←	1	1	0	2	15326.6509	-0.0092
2	2	1	2	←	1	1	0	1	15327.2761	-0.0043
2	2	0	1	←	1	1	0	0	15333.5534	0.0062
2	2	0	3	←	1	1	0	2	15334.8005	0.0039
2	2	0	2	←	1	1	0	1	15335.3646	0.0041
2	2	1	2	←	1	1	1	1	15491.7259	-0.0070
2	2	1	3	←	1	1	1	2	15492.7574	0.0085
2	2	1	1	←	1	1	1	0	15493.9256	0.0027

Table S18. Measured frequencies (ν_{obs}) and residuals ($\nu_{obs} - \nu_{cal}$) of the rotational transitions of the $^{13}C_3$ isotopic species of prolinol I.

J'	*K'*$_{-1}$	*K'*$_{+1}$	*F*	←	*J"*	*K"*$_{-1}$	*K"*$_{+1}$	*F"*	ν_{obs} /MHz	ν_{obs}- ν_{calc}/MHz
1	0	1	0	←	0	0	0	1	3907.9012	-0.0033
1	0	1	2	←	0	0	0	1	3908.4217	-0.0009
1	0	1	1	←	0	0	0	1	3908.7706	0.0026
2	0	2	1	←	1	1	0	0	5206.6663	0.0018
2	0	2	3	←	1	1	0	2	5208.3985	0.0017
2	0	2	1	←	1	1	0	1	5209.1558	-0.0001
2	0	2	2	←	1	1	0	1	5209.8209	0.0003
2	0	2	2	←	1	1	1	1	5373.8269	0.0053
2	0	2	3	←	1	1	1	2	5374.0464	0.0012
2	0	2	2	←	1	1	1	2	5374.4869	0.0143
2	0	2	1	←	1	1	1	0	5374.7970	0.0125
1	1	0	1	←	0	0	0	1	6507.7730	-0.0027
1	1	0	2	←	0	0	0	1	6508.7704	-0.0018
3	2	2	2	←	3	1	2	2	7061.0838	0.0025
3	2	2	4	←	3	1	2	4	7061.2944	0.0003
3	2	2	3	←	3	1	2	3	7061.9238	0.0215
2	1	2	1	←	1	1	1	1	7650.2063	0.0038
2	1	2	3	←	1	1	1	2	7651.4451	-0.0016
2	1	2	2	←	1	1	1	1	7651.8737	0.0103
2	1	2	2	←	1	1	1	2	7652.5002	-0.0142
2	0	2	1	←	1	0	1	1	7808.1609	-0.0026
2	0	2	1	←	1	0	1	0	7809.0262	-0.0008
2	0	2	2	←	1	0	1	2	7809.1748	0.0011
2	2	0	1	←	2	1	2	1	7809.6034	-0.0067
2	1	1	1	←	1	1	0	0	7981.2106	-0.0023
2	1	1	2	←	1	1	0	2	7981.6255	0.0031
2	1	1	3	←	1	1	0	2	7982.3186	-0.0014
2	1	1	2	←	1	1	0	1	7982.6190	0.0001
2	1	1	1	←	1	1	0	1	7983.7027	-0.0015
3	0	3	4	←	2	1	1	3	8918.8989	-0.0033
3	0	3	3	←	2	1	1	2	8920.1278	-0.0033
2	1	2	1	←	1	0	1	1	10085.2057	-0.0036
2	1	2	3	←	1	0	1	2	10086.1422	-0.0055
2	1	2	2	←	1	0	1	1	10086.8735	0.0035
2	1	2	2	←	1	0	1	2	10087.2097	-0.0057
2	1	1	2	←	1	0	1	1	10581.6271	0.0005
2	1	1	2	←	1	0	1	2	10581.9727	0.0008
2	1	1	3	←	1	0	1	2	10582.6645	-0.0051
2	1	1	1	←	1	0	1	0	10583.5774	0.0021
3	1	3	2	←	2	1	2	2	11470.7283	-0.0049
3	1	3	4	←	2	1	2	3	11472.2256	-0.0025
3	1	3	3	←	2	1	2	2	11472.3852	-0.0013
3	1	3	2	←	2	1	2	1	11472.3852	-0.0013
3	0	3	2	←	2	0	2	2	11692.2119	-0.0004
3	0	3	4	←	2	0	2	3	11692.8141	-0.0113
3	0	3	3	←	2	0	2	2	11692.9339	0.0044
3	0	3	3	←	2	0	2	3	11693.3531	-0.0037
3	2	2	2	←	2	2	1	1	11725.1827	0.0024
3	2	2	4	←	2	2	1	3	11725.3908	0.0048
3	2	2	3	←	2	2	1	2	11725.7597	0.0036
3	2	1	4	←	2	2	0	3	11757.8487	0.0022
3	2	1	3	←	2	2	0	2	11758.1248	-0.0015
3	1	2	3	←	2	1	1	3	11967.5876	0.0006
3	1	2	2	←	2	1	1	1	11968.0196	-0.0008
3	1	2	4	←	2	1	1	3	11968.2136	0.0184
3	1	2	2	←	2	1	1	2	11969.1050	-0.0008
4	0	4	3	←	3	1	2	2	12503.5892	-0.0023
4	0	4	5	←	3	1	2	4	12503.9690	-0.0053
4	0	4	4	←	3	1	2	3	12505.2424	-0.0002
3	1	3	2	←	2	0	2	1	13749.4526	0.0130
3	1	3	4	←	2	0	2	3	13749.6320	0.0024

3	1	3	3	←	2	0	2	2	13750.4187	-0.0047
3	1	2	4	←	2	0	2	3	14742.1176	-0.0008
2	2	0	3	←	1	1	0	2	15294.5763	-0.0012
2	2	1	3	←	1	1	1	2	15452.0657	-0.0061
4	0	4	5	←	3	0	3	4	15553.2719	-0.0013
4	0	4	3	←	3	0	3	2	15553.2719	-0.0013
4	0	4	4	←	3	0	3	3	15553.3919	-0.0042

Table S19. Measured frequencies (ν_{obs}) and residuals ($\nu_{obs} - \nu_{cal}$) of the rotational transitions of the ^{13}**C**$_4$ isotopic species of prolinol **I**.

J'	*K'*$_{-1}$	*K'*$_{+1}$	*F*	←	*J"*	*K"*$_{-1}$	*K"*$_{+1}$	*F"*	ν_{obs} /MHz	ν_{obs}- ν_{calc}/MHz
1	0	1	0	←	0	0	0	1	3935.8960	-0.0037
1	0	1	2	←	0	0	0	1	3936.3996	-0.0003
1	0	1	1	←	0	0	0	1	3936.7349	0.0014
2	0	2	1	←	1	1	0	0	5301.7162	0.0052
2	0	2	3	←	1	1	0	2	5303.4151	0.0006
2	0	2	2	←	1	1	0	2	5303.8343	0.0016
2	0	2	1	←	1	1	0	1	5304.1587	-0.0041
2	0	2	2	←	1	1	0	1	5304.8111	-0.0023
2	0	2	2	←	1	1	1	1	5478.5267	0.0048
2	0	2	3	←	1	1	1	2	5478.7578	0.0072
2	0	2	1	←	1	1	1	0	5479.4986	0.0096
1	1	0	1	←	0	0	0	1	6495.5591	0.0044
1	1	0	2	←	0	0	0	1	6496.5324	-0.0030
2	2	1	1	←	2	1	1	1	7154.2279	0.0146
2	2	1	3	←	2	1	1	3	7154.4100	0.0101
2	2	1	2	←	2	1	1	2	7154.7405	0.0046
2	1	2	1	←	1	1	1	1	7696.4772	-0.0016
2	1	2	3	←	1	1	1	2	7697.7064	-0.0031
2	1	2	1	←	1	1	1	0	7698.1027	-0.0054
2	1	2	2	←	1	1	1	1	7698.1027	-0.0054
2	1	2	2	←	1	1	1	2	7698.7538	-0.0065
2	0	2	1	←	1	0	1	1	7862.9838	-0.0002
2	0	2	3	←	1	0	1	2	7863.5632	0.0134
2	0	2	1	←	1	0	1	0	7863.8199	0.0022
2	0	2	2	←	1	0	1	2	7863.9679	-0.0003
2	1	1	1	←	1	1	0	0	8046.8754	-0.0023
2	1	1	2	←	1	1	0	2	8047.2718	0.0018
2	1	1	3	←	1	1	0	2	8047.9627	-0.0007
2	1	1	2	←	1	1	0	1	8048.2488	-0.0019
2	1	1	1	←	1	1	0	1	8049.3380	0.0085
3	0	3	2	←	2	1	1	1	9027.0841	-0.0011
3	0	3	4	←	2	1	1	3	9027.6566	0.0015
3	0	3	3	←	2	1	1	2	9028.8764	0.0005
2	1	2	3	←	1	0	1	2	10082.4970	-0.0118
2	1	2	2	←	1	0	1	1	10083.2235	-0.0025
2	1	1	2	←	1	0	1	1	10607.0750	0.0031
2	1	1	2	←	1	0	1	2	10607.4065	0.0011
2	1	1	3	←	1	0	1	2	10608.0945	-0.0043
2	1	1	1	←	1	0	1	0	10608.9868	0.0024
3	1	3	2	←	2	1	2	2	11539.4569	-0.0008
3	1	3	4	←	2	1	2	3	11540.9246	-0.0043
3	1	3	3	←	2	1	2	2	11541.0815	-0.0030
3	1	3	2	←	2	1	2	1	11541.0815	-0.0030
3	1	3	3	←	2	1	2	3	11542.1285	-0.0017
3	0	3	2	←	2	0	2	2	11771.6062	0.0050
3	0	3	4	←	2	0	2	3	11772.1948	-0.0092
3	0	3	3	←	2	0	2	2	11772.3169	0.0038
3	0	3	3	←	2	0	2	3	11772.7358	0.0044
3	2	2	2	←	2	2	1	1	11809.1112	-0.0051
3	2	2	4	←	2	2	1	3	11809.3117	-0.0032
3	2	2	3	←	2	2	1	2	11809.6701	-0.0022
3	2	1	2	←	2	2	0	1	11846.1503	0.0045
3	2	1	4	←	2	2	0	3	11846.3232	-0.0002
3	2	1	3	←	2	2	0	2	11846.5828	-0.0020
3	1	2	3	←	2	1	1	3	12065.3105	0.0060
3	1	2	2	←	2	1	1	1	12065.7399	0.0028
3	1	2	4	←	2	1	1	3	12065.9356	-0.0105
3	1	2	3	←	2	1	1	2	12065.9356	-0.0105
3	1	2	2	←	2	1	1	2	12066.8217	0.0059
4	0	4	5	←	3	1	2	4	12615.8141	0.0019

4	0	4	4	←	3	1	2	3	12617.0913	0.0103
3	1	3	2	←	2	0	2	1	13759.6991	-0.0006
3	1	3	4	←	2	0	2	3	13759.8784	-0.0093
3	1	3	3	←	2	0	2	2	13760.6626	-0.0081
3	1	2	3	←	2	0	2	2	14809.4310	-0.0041
3	1	2	4	←	2	0	2	3	14810.4599	0.0007
2	2	0	3	←	1	1	0	2	15211.6729	0.0074
2	2	1	3	←	1	1	1	2	15377.6851	-0.0145
4	0	4	5	←	3	0	3	4	15654.0794	0.0067
4	0	4	3	←	3	0	3	2	15654.0794	0.0067

Table S20. Measured frequencies (ν_{obs}) and residuals ($\nu_{obs} - \nu_{cal}$) of the rotational transitions of the $^{13}C_5$ isotopic species of prolinol I.

J'	*K'*$_{-1}$	*K'*$_{+1}$	*F*	←	*J"*	*K"*$_{-1}$	*K"*$_{+1}$	*F"*	ν_{obs} /MHz	ν_{obs}- ν_{calc}/MHz
1	0	1	0	←	0	0	0	1	3943.9919	-0.0023
1	0	1	2	←	0	0	0	1	3944.5059	-0.0002
1	0	1	1	←	0	0	0	1	3944.8457	-0.0016
2	0	2	1	←	1	1	0	0	5291.9297	0.0021
2	0	2	3	←	1	1	0	2	5293.6468	-0.0017
2	0	2	2	←	1	1	0	2	5294.0646	-0.0053
2	0	2	1	←	1	1	0	1	5294.4076	0.0021
2	0	2	2	←	1	1	0	1	5295.0602	-0.0008
2	0	2	1	←	1	1	1	2	5454.6372	0.0127
2	0	2	3	←	1	1	1	2	5454.8628	0.0042
2	0	2	1	←	1	1	1	0	5455.5858	-0.0134
1	1	0	1	←	0	0	0	1	6531.1531	-0.0245
1	1	0	2	←	0	0	0	1	6532.1691	0.0004
1	1	0	0	←	0	0	0	1	6533.6543	-0.0011
3	2	2	2	←	3	1	2	2	7042.7363	0.0022
3	2	2	4	←	3	1	2	4	7042.9438	-0.0034
3	2	2	3	←	3	1	2	3	7043.5531	-0.0025
2	2	1	1	←	2	1	1	1	7279.1768	0.0021
2	2	1	3	←	2	1	1	3	7279.3619	0.0036
2	2	1	2	←	2	1	1	2	7279.6939	0.0051
2	1	2	3	←	1	1	1	2	7728.0538	0.0032
2	1	2	2	←	1	1	1	1	7728.4563	-0.0064
2	1	2	2	←	1	1	1	2	7729.1091	-0.0035
2	0	2	1	←	1	0	1	1	7880.7348	-0.0009
2	0	2	2	←	1	0	1	1	7881.3249	-0.0141
2	0	2	3	←	1	0	1	2	7881.3249	-0.0141
2	0	2	1	←	1	0	1	0	7881.5888	0.0000
2	0	2	2	←	1	0	1	2	7881.7318	-0.0006
2	1	1	1	←	1	1	0	0	8048.9492	0.0001
2	1	1	2	←	1	1	0	2	8049.3380	-0.0147
2	1	1	3	←	1	1	0	2	8050.0487	-0.0001
2	1	1	2	←	1	1	0	1	8050.3420	-0.0017
2	1	1	1	←	1	1	0	1	8051.4216	-0.0053
3	0	3	2	←	2	1	1	1	9045.6908	0.0042
3	0	3	4	←	2	1	1	3	9046.2674	0.0109
3	0	3	2	←	2	1	1	2	9046.7769	0.0023
3	0	3	3	←	2	1	1	3	9046.7769	0.0023
3	0	3	3	←	2	1	1	2	9047.4745	-0.0012
2	1	2	3	←	1	0	1	2	10154.4997	-0.0034
2	1	2	2	←	1	0	1	1	10155.2232	-0.0006
2	1	2	2	←	1	0	1	2	10155.5549	-0.0100
2	1	1	2	←	1	0	1	1	10636.6759	0.0020
2	1	1	2	←	1	0	1	2	10637.0151	0.0000
2	1	1	3	←	1	0	1	2	10637.7046	-0.0067
2	1	1	1	←	1	0	1	0	10638.6103	0.0000
3	1	3	2	←	2	1	2	2	11585.8923	0.0052
3	1	3	4	←	2	1	2	3	11587.3758	0.0020
3	1	3	3	←	2	1	2	2	11587.5330	0.0021

3	1	3	2	←	2	1	2	1	11587.5330	0.0021
3	1	3	3	←	2	1	2	3	11588.6063	0.0189
3	0	3	2	←	2	0	2	2	11802.0544	0.0019
3	0	3	4	←	2	0	2	3	11802.6367	-0.0202
3	2	2	2	←	2	2	1	1	11833.4386	0.0065
3	2	2	4	←	2	2	1	3	11833.6349	-0.0004
3	2	2	3	←	2	2	1	2	11834.0008	-0.0003
3	2	1	2	←	2	2	0	1	11864.3227	-0.0063
3	2	1	4	←	2	2	0	3	11864.5193	0.0064
3	2	1	3	←	2	2	0	2	11864.7960	0.0055
3	1	2	3	←	2	1	1	3	12069.4427	0.0047
3	1	2	2	←	2	1	1	1	12069.8749	0.0024
3	1	2	4	←	2	1	1	3	12070.0726	-0.0097
3	1	2	3	←	2	1	1	2	12070.0726	-0.0097
3	1	2	2	←	2	1	1	2	12070.9571	0.0013
4	0	4	3	←	3	1	2	2	12677.3014	-0.0082
4	0	4	5	←	3	1	2	4	12677.6940	0.0046
3	1	3	4	←	2	0	2	3	13860.5639	-0.0019
3	1	3	3	←	2	0	2	2	13861.3567	-0.0012
3	1	2	3	←	2	0	2	2	14825.4223	0.0054
3	1	2	4	←	2	0	2	3	14826.4519	0.0051
3	1	2	2	←	2	0	2	1	14826.9050	0.0110
2	2	1	3	←	1	1	0	2	15329.4152	0.0080
2	2	0	3	←	1	1	0	2	15337.1620	-0.0005
2	2	0	2	←	1	1	0	1	15337.7367	0.0045
2	2	1	2	←	1	1	1	1	15489.6065	0.0048
2	2	1	1	←	1	1	1	1	15490.1971	-0.0141
2	2	1	2	←	1	1	1	2	15490.1971	-0.0141
2	2	1	3	←	1	1	1	2	15490.6203	0.0030
4	0	4	5	←	3	0	3	4	15701.4900	0.0047
4	0	4	3	←	3	0	3	2	15701.4900	0.0047
4	0	4	4	←	3	0	3	3	15701.5988	-0.0063

Table S21. Measured frequencies (ν_{obs}) and residuals ($\nu_{obs} - \nu_{cal}$) of the rotational transitions of the **$^{13}C_6$** isotopic species of prolinol I.

J'	*K'*$_{-1}$	*K'*$_{+1}$	*F*	←	*J"*	*K"*$_{-1}$	*K"*$_{+1}$	*F"*	ν_{obs} /MHz	ν_{obs}- ν_{calc}/MHz
1	0	1	2	←	0	0	0	1	3903.3432	-0.0019
1	0	1	1	←	0	0	0	1	3903.6908	-0.0051
2	0	2	1	←	1	1	0	0	5169.5344	-0.0034
2	0	2	3	←	1	1	0	2	5171.2713	-0.0025
2	0	2	1	←	1	1	0	1	5172.0425	0.0105
2	0	2	2	←	1	1	1	1	5332.6288	0.0093
2	0	2	3	←	1	1	1	2	5332.8344	-0.0009
2	0	2	1	←	1	1	1	0	5333.5615	-0.0045
1	1	0	1	←	0	0	0	1	6530.1561	0.0028
1	1	0	2	←	0	0	0	1	6531.1531	0.0021
1	1	0	0	←	0	0	0	1	6532.6545	0.0070
3	2	2	2	←	3	1	2	2	7161.5205	-0.0027
3	2	2	4	←	3	1	2	4	7161.7433	0.0091
3	2	2	3	←	3	1	2	3	7162.3375	0.0006
2	1	2	1	←	1	1	1	1	7644.1352	-0.0032
2	1	2	3	←	1	1	1	2	7645.3770	-0.0020
2	1	2	2	←	1	1	1	1	7645.8138	0.0126
2	1	2	2	←	1	1	1	2	7646.4433	-0.0045
2	0	2	1	←	1	0	1	1	7798.4972	0.0077
2	0	2	3	←	1	0	1	2	7799.0925	0.0128
2	0	2	1	←	1	0	1	0	7799.3628	-0.0037
2	0	2	2	←	1	0	1	2	7799.5086	-0.0022
2	1	1	1	←	1	1	0	0	7966.9707	0.0031
2	1	1	2	←	1	1	0	2	7967.3815	-0.0045
2	1	1	3	←	1	1	0	2	7968.0786	-0.0003

2	1	1	2	←	1	1	0	1	7968.3820	-0.0016
2	1	1	1	←	1	1	0	1	7969.4608	-0.0009
3	0	3	2	←	2	1	1	1	8882.1482	-0.0018
3	0	3	4	←	2	1	1	3	8882.7205	-0.0008
2	1	2	1	←	1	0	1	1	10110.6824	0.0036
2	1	2	1	←	1	0	1	0	10111.6170	0.0065
2	1	2	3	←	1	0	1	2	10111.6170	0.0065
2	1	2	2	←	1	0	1	1	10112.3401	-0.0015
2	1	2	2	←	1	0	1	2	10112.6898	-0.0026
2	1	1	2	←	1	0	1	1	10594.8450	0.0040
2	1	1	2	←	1	0	1	2	10595.1940	0.0022
2	1	1	3	←	1	0	1	2	10595.8831	-0.0070
2	1	1	1	←	1	0	1	1	10595.8831	-0.0070
2	1	1	1	←	1	0	1	0	10596.7953	-0.0009
3	1	3	2	←	2	1	2	2	11461.9211	-0.0028
3	1	3	4	←	2	1	2	3	11463.4180	-0.0029
3	1	3	3	←	2	1	2	2	11463.5741	-0.0058
3	1	3	2	←	2	1	2	1	11463.5741	-0.0058
3	1	3	3	←	2	1	2	3	11464.6357	-0.0084
3	0	3	2	←	2	0	2	2	11678.9161	0.0067
3	0	3	4	←	2	0	2	3	11679.5211	-0.0053
3	0	3	3	←	2	0	2	2	11679.6206	-0.0067
3	0	3	3	←	2	0	2	3	11680.0596	0.0014
3	2	2	2	←	2	2	1	1	11709.9442	-0.0017
3	2	2	4	←	2	2	1	3	11710.1547	0.0000
3	2	2	3	←	2	2	1	2	11710.5275	-0.0032
3	2	1	2	←	2	2	0	1	11740.4781	-0.0164
3	2	1	4	←	2	2	0	3	11740.6780	-0.0062
3	2	1	3	←	2	2	0	2	11740.9647	-0.0084
3	1	2	3	←	2	1	1	3	11946.5475	-0.0016
3	1	2	2	←	2	1	1	1	11946.9783	0.0008
3	1	2	4	←	2	1	1	3	11947.1708	0.0191
3	1	2	2	←	2	1	1	2	11948.0548	-0.0009
4	0	4	3	←	3	1	2	2	12472.8712	-0.0016
4	0	4	5	←	3	1	2	4	12473.2566	0.0040
4	0	4	4	←	3	1	2	3	12474.5182	0.0061
3	1	3	2	←	2	0	2	1	13775.7733	-0.0027
3	1	3	4	←	2	0	2	3	13775.9671	0.0025
3	1	2	3	←	2	0	2	2	14742.9318	0.0087
3	1	2	4	←	2	0	2	3	14743.9606	0.0038
3	1	2	2	←	2	0	2	1	14744.4023	-0.0050
4	1	4	3	←	3	1	3	2	15276.0885	-0.0106
4	1	4	4	←	3	1	3	3	15276.0885	-0.0106
2	2	1	2	←	1	1	1	1	15527.3320	-0.0168
2	2	1	3	←	1	1	1	2	15528.3780	0.0065
4	0	4	5	←	3	0	3	4	15537.7109	0.0214
4	0	4	3	←	3	0	3	2	15537.7109	0.0214

Table S22. Measured frequencies (ν_{obs}) and residuals ($\nu_{obs} - \nu_{cal}$) of the rotational transitions of the **^{15}N** isotopic species of prolinol I.

J'	K'_{-1}	K'_{+1}	←	J''	K''_{-1}	K''_{+1}	ν_{obs} /MHz	ν_{obs}- ν_{calc}/MHz
2	0	2	←	1	1	0	5295.7051	0.0006
1	1	0	←	0	0	0	6514.3744	-0.0030
2	1	2	←	1	1	1	7703.8177	-0.0042
2	0	2	←	1	0	1	7870.2691	-0.0032
3	2	1	←	3	1	3	8039.5125	0.0002

2	1	1	←	1	1	0	8055.4003	-0.0016
3	0	3	←	2	1	1	9022.5106	-0.0050
3	0	3	←	2	1	2	9549.8776	-0.0040
2	1	2	←	1	0	1	10102.6020	-0.0017
2	1	1	←	1	0	1	10629.9664	-0.0034
3	1	3	←	2	1	2	11549.9634	0.0018
3	0	3	←	2	0	2	11782.2123	-0.0008
3	2	2	←	2	2	1	11819.4525	-0.0037
3	2	1	←	2	2	0	11856.5274	-0.0034
3	1	2	←	2	1	1	12077.0371	-0.0051
4	3	2	←	4	2	2	12325.8875	-0.0075
4	0	4	←	3	1	2	12612.7776	0.0060
4	0	4	←	3	1	3	13667.2197	0.0014
3	1	3	←	2	0	2	13782.2999	0.0069
3	1	2	←	2	0	2	14836.7531	0.0134
2	2	0	←	1	1	0	15260.9871	0.0081
4	0	4	←	3	0	3	15667.2943	-0.0040

Table S23. Measured frequencies (ν_{obs}) and residuals ($\nu_{obs} - \nu_{cal}$) of the rotational transitions of the **^{18}O** isotopic species of prolinol **I**.

J'	*K'*$_{-1}$	*K'*$_{+1}$	*F*	←	*J"*	*K"*$_{-1}$	*K"*$_{+1}$	*F"*	ν_{obs} /MHz	ν_{obs}- ν_{calc}/MHz
1	0	1	0	←	0	0	0	1	3828.9815	-0.0018
1	0	1	2	←	0	0	0	1	3829.4324	0.0045
1	0	1	1	←	0	0	0	1	3829.7350	0.0108
2	1	2	3	←	1	1	1	2	7513.8846	-0.0026
2	1	2	2	←	1	1	1	2	7514.8853	-0.0070
2	0	2	1	←	1	0	1	1	7652.1588	0.0000
2	0	2	3	←	1	0	1	2	7652.6607	0.0025
2	0	2	1	←	1	0	1	0	7652.8908	-0.0087
2	0	2	2	←	1	0	1	2	7653.0220	-0.0017
2	1	1	1	←	1	1	0	0	7802.8538	-0.0084
2	1	1	3	←	1	1	0	2	7803.8839	-0.0033
2	1	1	2	←	1	1	0	1	7804.1358	-0.0020
2	1	1	1	←	1	1	0	1	7805.2208	0.0132
3	1	3	2	←	2	1	2	2	11265.6450	0.0160
3	1	3	4	←	2	1	2	3	11267.0267	-0.0062
3	1	3	3	←	2	1	2	2	11267.1744	-0.0032
3	1	3	2	←	2	1	2	1	11267.1744	-0.0032
3	0	3	4	←	2	0	2	3	11463.4179	0.0008
3	2	2	2	←	2	2	1	1	11488.2028	-0.0094
3	2	2	4	←	2	2	1	3	11488.3878	-0.0009
3	2	2	3	←	2	2	1	2	11488.7044	-0.0018
3	2	1	4	←	2	2	0	3	11513.2581	0.0075
3	1	2	2	←	2	1	1	1	11701.6025	-0.0086
3	1	2	4	←	2	1	1	3	11701.7874	0.0092
4	0	4	5	←	3	0	3	4	15255.9067	0.0030

Table S24. Measured frequencies (ν_{obs}) and residuals ($\nu_{obs} - \nu_{cal}$) of the rotational transitions of the parent species of prolinol conformer **II**.

J'	*K'*$_{-1}$	*K'*$_{+1}$	*F*	←	*J"*	*K"*$_{-1}$	*K"*$_{+1}$	*F"*	ν_{obs} /MHz	ν_{obs}- ν_{calc}/MHz
4	1	3	3	←	4	1	4	3	3047.7990	-0.0064
4	1	3	5	←	4	1	4	5	3047.8907	0.0020
4	1	3	4	←	4	1	4	4	3048.2127	0.0002
2	1	2	2	←	2	0	2	1	3067.3536	0.0102
2	1	2	2	←	2	0	2	1	3067.3536	0.0102
2	1	2	2	←	2	0	2	3	3067.5858	-0.0035
2	1	2	3	←	2	0	2	3	3067.9768	-0.0073
2	1	2	2	←	2	0	2	2	3067.9768	-0.0073
2	1	2	3	←	2	0	2	2	3068.4016	0.0016

2	1	2	1	←	2	0	2	2	3068.6091	0.0043
2	0	2	2	←	1	1	0	1	3263.0211	-0.0053
2	0	2	2	←	1	1	0	2	3263.3646	-0.0058
2	0	2	3	←	1	1	0	2	3263.8116	-0.0011
2	0	2	1	←	1	1	0	0	3264.5719	-0.0024
1	1	1	1	←	1	0	1	0	3352.7954	0.0027
1	1	1	1	←	1	0	1	0	3352.7954	0.0028
1	1	1	2	←	1	0	1	2	3353.3513	-0.0005
1	1	1	1	←	1	0	1	2	3353.3513	-0.0005
1	1	1	2	←	1	0	1	2	3353.3513	0.0139
1	1	1	0	←	1	0	1	1	3353.6439	-0.0087
1	1	1	0	←	1	0	1	1	3353.6439	-0.0086
1	1	1	2	←	1	0	1	1	3353.7674	0.0070
1	1	1	1	←	1	0	1	1	3353.7674	0.0070
1	0	1	1	←	0	0	0	1	3470.8482	-0.0041
1	0	1	2	←	0	0	0	1	3471.2506	-0.0032
1	0	1	0	←	0	0	0	1	3471.8538	-0.0022
2	0	2	2	←	1	1	1	1	3568.6408	0.0065
2	0	2	2	←	1	1	1	2	3568.6408	0.0065
2	0	2	3	←	1	1	1	2	3569.1316	-0.0025
2	0	2	1	←	1	1	1	1	3569.3288	0.0065
2	0	2	1	←	1	1	1	0	3569.4616	-0.0045
1	1	0	1	←	1	0	1	0	3658.4055	0.0051
1	1	0	0	←	1	0	1	1	3658.5449	0.0005
1	1	0	2	←	1	0	1	2	3658.6620	0.0032
1	1	0	1	←	1	0	1	2	3659.0339	0.0024
1	1	0	2	←	1	0	1	1	3659.0339	0.0024
1	1	0	1	←	1	0	1	1	3659.4082	0.0040
2	1	1	2	←	2	0	2	1	3983.6656	-0.0220
2	1	1	1	←	2	0	2	1	3983.7900	0.0065
2	1	1	3	←	2	0	2	3	3983.9917	-0.0033
2	1	1	2	←	2	0	2	2	3984.3808	0.0052
3	1	2	3	←	3	0	3	2	4507.9657	0.0082
3	1	2	3	←	3	0	3	4	4508.1836	0.0591
3	1	2	2	←	3	0	3	2	4508.1836	-0.0164
3	1	2	4	←	3	0	3	4	4508.3120	0.0078
3	1	2	3	←	3	0	3	3	4508.6051	0.0034
3	1	2	4	←	3	0	3	3	4508.8168	0.0042
3	1	2	2	←	3	0	3	3	4508.8168	0.0042
5	1	4	4	←	5	1	5	4	4557.7169	0.0042
5	1	4	6	←	5	1	5	6	4557.7169	0.0042
5	1	4	5	←	5	1	5	5	4558.0648	-0.0010
4	1	3	4	←	3	2	1	3	4738.6968	0.0100
4	1	3	5	←	3	2	1	4	4738.9774	0.0235
4	1	3	3	←	4	0	4	3	5270.9153	-0.0054
4	1	3	5	←	4	0	4	5	5270.9153	-0.0054
4	1	3	4	←	4	0	4	4	5271.2284	0.0045
3	0	3	3	←	2	1	1	2	6349.9984	0.0026
3	0	3	4	←	2	1	1	3	6350.4027	-0.0086
3	0	3	2	←	2	1	1	1	6350.5479	0.0039
2	1	2	2	←	1	1	1	1	6636.6534	-0.0124
2	1	2	3	←	1	1	1	2	6637.0896	-0.0022
2	1	2	1	←	1	1	1	1	6637.2387	-0.0004
2	1	2	1	←	1	1	1	0	6637.3817	-0.0012
1	1	1	2	←	0	0	0	1	6824.5769	-0.0142
2	0	2	2	←	1	0	1	2	6922.0889	0.0107
2	0	2	1	←	1	0	1	0	6922.0889	0.0107
2	0	2	3	←	1	0	1	2	6922.4829	0.0114
2	0	2	1	←	1	0	1	2	6922.7159	-0.0013
2	0	2	1	←	1	0	1	1	6923.1131	-0.0056
1	1	0	0	←	0	0	0	1	7129.3942	-0.0024
1	1	0	2	←	0	0	0	1	7129.9101	-0.0025
1	1	0	1	←	0	0	0	1	7130.2542	-0.0023
2	1	1	2	←	1	1	0	1	7247.4044	0.0024
2	1	1	2	←	1	1	0	2	7247.8060	0.0077

2	1	1	3	←	1	1	0	2	7247.8060	0.0077
2	1	1	1	←	1	1	0	0	7248.3573	-0.0006
3	0	3	3	←	2	1	2	2	7266.3469	0.0070
3	0	3	4	←	2	1	2	3	7266.4505	0.0020
4	0	4	4	←	3	1	2	3	9181.7030	-0.0059
4	0	4	5	←	3	1	2	4	9182.0443	0.0029
3	2	2	4	←	3	1	2	4	9615.0060	-0.0033
3	2	2	3	←	3	1	2	3	9615.1875	-0.0014
3	2	1	2	←	3	1	2	2	9714.0227	0.0127
3	2	1	4	←	3	1	2	4	9714.0227	0.0127
3	2	1	3	←	3	1	2	3	9714.2509	0.0049
3	1	3	3	←	2	1	2	3	9942.9401	-0.0042
3	1	3	3	←	2	1	2	2	9943.3142	0.0013
3	1	3	4	←	2	1	2	3	9943.4474	0.0056
3	1	3	2	←	2	1	2	2	9943.9839	-0.0006
2	1	2	1	←	1	0	1	0	9990.0407	-0.0034
2	1	2	2	←	1	0	1	2	9990.0407	-0.0034
2	1	2	3	←	1	0	1	2	9990.4223	-0.0069
2	1	2	1	←	1	0	1	1	9991.0368	0.0014
2	2	1	1	←	2	1	1	1	10059.8037	0.0072
2	2	1	3	←	2	1	1	3	10060.0704	0.0006
2	2	1	2	←	2	1	1	2	10060.5618	0.0002
2	2	0	1	←	2	1	1	1	10079.7113	0.0057
2	2	0	3	←	2	1	1	3	10079.9910	0.0053
2	2	0	2	←	2	1	1	2	10080.4895	-0.0001
3	0	3	3	←	2	0	2	3	10333.9269	-0.0022
3	0	3	3	←	2	0	2	2	10334.4145	0.0225
3	0	3	4	←	2	0	2	3	10334.4145	0.0225
3	0	3	2	←	2	0	2	3	10334.5909	0.0176
3	0	3	2	←	2	0	2	2	10335.0164	0.0008
3	2	2	3	←	2	2	1	2	10413.2243	-0.0005
3	2	2	4	←	2	2	1	3	10413.6542	-0.0008
3	2	2	2	←	2	2	1	1	10413.8941	0.0001
3	2	1	2	←	2	2	0	2	10492.3579	0.0086
3	2	1	3	←	2	2	0	2	10492.3579	0.0086
3	2	1	2	←	2	2	0	3	10492.7639	-0.0037
3	2	1	4	←	2	2	0	3	10492.7639	-0.0037
3	2	1	3	←	2	2	0	3	10492.7639	-0.0037
3	2	1	2	←	2	2	0	1	10492.9993	-0.0008
3	1	2	3	←	2	1	1	2	10858.5952	-0.0024
3	1	2	4	←	2	1	1	3	10858.7130	-0.0116
3	1	2	2	←	2	1	1	1	10858.7130	-0.0116
2	1	1	1	←	1	0	1	0	10905.8937	-0.0047
2	1	1	2	←	1	0	1	2	10906.4622	0.0052
2	1	1	3	←	1	0	1	2	10906.4622	0.0052
2	1	1	2	←	1	0	1	1	10906.8069	0.0007
2	2	1	1	←	2	1	2	1	10975.6560	-0.0073
2	2	1	2	←	2	1	2	2	10976.8967	-0.0090
2	2	0	1	←	2	1	2	1	10995.5727	0.0003
2	2	0	3	←	2	1	2	3	10996.0217	-0.0012
2	2	0	2	←	2	1	2	2	10996.8338	0.0001
4	0	4	3	←	3	1	3	2	11013.3479	0.0109
4	0	4	4	←	3	1	3	3	11013.3479	0.0109
4	0	4	5	←	3	1	3	4	11013.3479	0.0109
3	2	2	2	←	3	1	3	2	11446.1402	-0.0059
3	2	2	4	←	3	1	3	4	11446.3172	-0.0030
3	2	1	2	←	3	1	3	2	11545.1517	-0.0096
3	2	1	4	←	3	1	3	4	11545.3453	-0.0010
3	2	1	3	←	3	1	3	3	11545.8791	0.0043
5	0	5	5	←	4	1	3	4	11709.3325	0.0083
5	0	5	6	←	4	1	3	5	11709.6235	-0.0124
4	2	3	5	←	4	1	4	5	12079.1379	0.0087
4	2	3	4	←	4	1	4	4	12079.4994	-0.0041
4	2	2	5	←	4	1	4	5	12372.3775	-0.0123
4	2	2	4	←	4	1	4	4	12372.8212	0.0012

6	1	5	7	←	5	2	3	6	12572.2368	-0.0138
3	1	3	3	←	2	0	2	3	13010.9013	-0.0008
3	1	3	2	←	2	0	2	1	13011.3388	0.0011
3	1	3	3	←	2	0	2	2	13011.3388	0.0011
3	1	3	4	←	2	0	2	3	13011.4049	0.0054
3	1	3	2	←	2	0	2	2	13012.0158	-0.0002
4	1	4	4	←	3	1	3	4	13235.8474	-0.0041
4	1	4	4	←	3	1	3	3	13236.3161	-0.0329
4	1	4	3	←	3	1	3	2	13236.4348	0.0345
4	1	4	5	←	3	1	3	4	13236.4348	0.0345
4	1	4	3	←	3	1	3	3	13237.0524	-0.0005
5	2	3	4	←	5	1	5	4	13545.4629	0.0067
5	2	3	6	←	5	1	5	6	13545.4629	0.0067
5	2	3	5	←	5	1	5	5	13545.8787	-0.0073
4	0	4	4	←	3	0	3	4	13689.8372	0.0037
4	0	4	5	←	3	0	3	4	13690.3109	-0.0347
4	0	4	3	←	3	0	3	2	13690.3109	0.0004
4	0	4	4	←	3	0	3	3	13690.3109	0.0004
4	0	4	3	←	3	0	3	3	13690.9608	0.0064
4	2	3	4	←	3	2	2	3	13869.0330	-0.0019
4	2	3	5	←	3	2	2	4	13869.1964	-0.0240
4	2	3	3	←	3	2	2	2	13869.3160	0.0479
4	3	2	4	←	3	3	1	3	13921.9848	0.0050
4	3	2	5	←	3	3	1	4	13922.3741	0.0019
4	3	2	3	←	3	3	1	2	13922.5266	0.0001
4	3	2	4	←	3	3	1	4	13922.5266	0.0001
4	3	1	3	←	3	3	0	3	13926.6336	-0.0034
4	3	1	4	←	3	3	0	3	13926.8439	-0.0077
4	3	1	5	←	3	3	0	4	13927.2375	-0.0051
4	3	1	3	←	3	3	0	2	13927.3977	0.0009
4	3	1	4	←	3	3	0	4	13927.3977	0.0009
4	2	2	4	←	3	2	1	3	14063.2985	0.0024
4	2	2	4	←	3	2	1	4	14063.2985	0.0024
4	2	2	5	←	3	2	1	4	14063.4371	-0.0179
4	2	2	3	←	3	2	1	3	14063.4371	-0.0179
4	1	3	4	←	3	1	2	4	14452.7516	-0.0017
4	1	3	4	←	3	1	2	3	14452.8841	-0.0487
4	1	3	3	←	3	1	2	2	14453.0001	0.0130
4	1	3	3	←	3	1	2	3	14453.2276	-0.0019
5	0	5	6	←	4	1	4	5	14757.5437	0.0136
5	0	5	5	←	4	1	4	4	14757.5437	0.0136
3	1	2	3	←	2	0	2	3	14842.5253	-0.0055
3	1	2	4	←	2	0	2	3	14842.7078	-0.0026
3	1	2	3	←	2	0	2	2	14842.9751	0.0020
3	1	2	2	←	2	0	2	2	14843.2234	0.0078
5	1	5	5	←	4	1	4	5	16512.6830	0.0165
5	1	5	5	←	4	1	4	4	16513.1844	-0.0419
5	1	5	6	←	4	1	4	5	16513.2813	0.0164
5	1	5	4	←	4	1	4	4	16513.9369	-0.0098
5	0	5	5	←	4	0	4	4	16980.5964	0.0298
5	0	5	4	←	4	0	4	3	16980.5964	0.0298
5	0	5	6	←	4	0	4	5	16980.5964	0.0298
5	2	4	5	←	4	2	3	4	17311.4756	0.0495
5	2	4	6	←	4	2	3	5	17311.5614	0.0308
5	2	4	4	←	4	2	3	3	17311.5614	0.0308
2	2	0	3	←	1	1	0	2	17327.8108	0.0175
2	2	0	1	←	1	1	0	0	17328.0644	0.0009
2	2	1	3	←	1	1	1	2	17613.2008	0.0020
2	2	1	2	←	1	1	1	1	17613.5608	-0.0107
5	2	3	6	←	4	2	2	5	17686.4035	0.0364
5	2	3	4	←	4	2	2	3	17686.4035	0.0364

Table S25. Measured frequencies (ν_{obs}) and residuals ($\nu_{obs} - \nu_{cal}$) of the rotational transitions of the $^{13}C_2$ isotopic species of prolinol II.

J'	K'_{-1}	K'_{+1}	F	←	J''	K''_{-1}	K''_{+1}	F''	ν_{obs} /MHz	ν_{obs}- ν_{calc}/MHz
1	0	1	1	←	0	0	0	1	3442.6556	-0.0002
1	0	1	2	←	0	0	0	1	3443.0404	-0.0025
2	1	2	2	←	1	1	1	1	6580.0415	-0.0093
2	1	2	3	←	1	1	1	2	6580.4629	-0.0001
2	1	2	1	←	1	1	1	0	6580.7298	0.0023
2	0	2	1	←	1	0	1	0	6865.3562	-0.0172
2	0	2	3	←	1	0	1	2	6865.7299	0.0136
2	0	2	1	←	1	0	1	1	6866.3340	-0.0075
2	1	1	2	←	1	1	0	1	7191.2010	-0.0062
2	1	1	2	←	1	1	0	2	7191.5989	0.0075
2	1	1	3	←	1	1	0	2	7191.5989	0.0075
2	1	1	1	←	1	1	0	2	7191.5989	0.0075
2	1	1	1	←	1	1	0	0	7192.1367	-0.0073
3	1	3	3	←	2	1	2	3	9857.7996	-0.0022
3	1	3	3	←	2	1	2	2	9858.1815	0.0049
3	1	3	4	←	2	1	2	3	9858.3117	0.0094
3	1	3	2	←	2	1	2	2	9858.8552	0.0031
3	0	3	3	←	2	0	2	3	10247.9913	-0.0016
3	0	3	4	←	2	0	2	3	10248.4677	0.0098
3	2	2	3	←	2	2	1	2	10328.6150	0.0033
3	2	2	4	←	2	2	1	3	10329.0310	0.0045
3	2	2	2	←	2	2	1	1	10329.2509	-0.0060
3	2	1	3	←	2	2	0	2	10409.0713	0.0055
3	2	1	4	←	2	2	0	3	10409.4537	-0.0057
3	2	1	2	←	2	2	0	1	10409.6903	0.0049
3	1	2	3	←	2	1	1	2	10774.0677	0.0035
3	1	2	4	←	2	1	1	3	10774.1709	-0.0068
4	1	4	4	←	3	1	3	4	13121.9977	0.0011
4	1	4	4	←	3	1	3	3	13122.4651	-0.0317
4	1	4	5	←	3	1	3	4	13122.5668	0.0084
4	1	4	3	←	3	1	3	3	13123.2042	0.0010
4	0	4	4	←	3	0	3	4	13573.8741	0.0016
4	0	4	4	←	3	0	3	3	13574.3307	-0.0084
4	0	4	3	←	3	0	3	2	13574.3307	-0.0084
4	0	4	3	←	3	0	3	3	13574.9679	-0.0011
4	2	3	4	←	3	2	2	3	13755.9497	0.0072
4	2	3	5	←	3	2	2	4	13756.0950	-0.0268
4	2	2	4	←	3	2	1	3	13953.3419	0.0017
4	2	2	3	←	3	2	1	3	13953.4840	-0.0076
4	2	2	5	←	3	2	1	4	13953.4840	-0.0076
4	1	3	5	←	3	1	2	4	14339.8226	0.0097

Table S26. Measured frequencies (ν_{obs}) and residuals ($\nu_{obs} - \nu_{cal}$) of the rotational transitions of the $^{13}C_3$ isotopic species of prolinol II.

J'	K'_{-1}	K'_{+1}	F	←	J''	K''_{-1}	K''_{+1}	F''	ν_{obs} /MHz	ν_{obs}- ν_{calc}/MHz
1	0	1	1	←	0	0	0	1	3418.0709	-0.0019
1	0	1	2	←	0	0	0	1	3418.4777	-0.0007
1	0	1	0	←	0	0	0	1	3419.0847	-0.0018
2	1	2	2	←	1	1	1	1	6539.9735	-0.0075
2	1	2	3	←	1	1	1	2	6540.4079	-0.0031
2	1	2	1	←	1	1	1	1	6540.5583	0.0026
2	1	2	1	←	1	1	1	0	6540.6960	-0.0113
2	0	2	2	←	1	0	1	2	6817.7578	-0.0258
2	0	2	1	←	1	0	1	0	6817.7578	-0.0258
2	0	2	3	←	1	0	1	2	6818.1874	0.0067
2	0	2	1	←	1	0	1	1	6818.8295	-0.0045
2	1	1	2	←	1	1	0	1	7132.9778	0.0019
2	1	1	2	←	1	1	0	2	7133.3759	0.0000
2	1	1	3	←	1	1	0	2	7133.3759	0.0000
2	1	1	1	←	1	1	0	0	7133.9447	0.0056

3	1	3	3	←	2	1	2	3	9798.6726	0.0001
3	1	3	3	←	2	1	2	2	9799.0392	-0.0028
3	1	3	4	←	2	1	2	3	9799.1714	-0.0005
3	0	3	3	←	2	0	2	3	10180.5707	-0.0007
3	0	3	4	←	2	0	2	3	10181.0599	0.0082
3	0	3	2	←	2	0	2	2	10181.6669	0.0011
3	2	2	3	←	2	2	1	2	10254.8964	0.0032
3	2	2	4	←	2	2	1	3	10255.3315	0.0039
3	2	2	2	←	2	2	1	1	10255.5713	0.0023
3	2	1	2	←	2	2	0	2	10329.0310	-0.0126
3	2	1	3	←	2	2	0	2	10329.0310	-0.0126
3	2	1	4	←	2	2	0	3	10329.4680	0.0013
3	2	1	3	←	2	2	0	3	10329.4680	0.0013
3	2	1	2	←	2	2	0	1	10329.7043	0.0026
3	1	2	3	←	2	1	1	2	10687.7927	-0.0007
3	1	2	4	←	2	1	1	3	10687.9159	-0.0054
3	1	2	2	←	2	1	1	1	10687.9159	-0.0054
2	1	1	1	←	1	0	1	0	10810.0811	-0.0037
2	1	1	3	←	1	0	1	2	10810.6586	0.0019
2	1	1	2	←	1	0	1	1	10810.9983	0.0010
4	1	4	4	←	3	1	3	4	13044.8010	0.0136
4	1	4	4	←	3	1	3	3	13045.2609	-0.0259
4	1	4	5	←	3	1	3	4	13045.3777	0.0282
4	1	4	3	←	3	1	3	3	13045.9932	-0.0002
4	0	4	4	←	3	0	3	4	13490.7324	0.0114
4	0	4	3	←	3	0	3	2	13491.2037	0.0042
4	0	4	5	←	3	0	3	4	13491.2037	-0.0317
4	0	4	3	←	3	0	3	3	13491.8432	-0.0047
4	2	2	4	←	3	2	1	3	13841.1832	0.0013
4	2	2	4	←	3	2	1	4	13841.1832	0.0013
4	2	2	5	←	3	2	1	4	13841.3338	-0.0098
4	2	2	3	←	3	2	1	3	13841.3338	-0.0098
4	1	3	3	←	3	1	2	2	14226.9266	0.0133
4	1	3	5	←	3	1	2	4	14226.9266	0.0133
5	0	5	5	←	4	0	4	4	16739.0529	-0.0021
5	0	5	4	←	4	0	4	3	16739.0529	-0.0021
5	0	5	6	←	4	0	4	5	16739.0529	-0.0021

Table S27. Measured frequencies (ν_{obs}) and residuals ($\nu_{obs} - \nu_{cal}$) of the rotational transitions of the $^{13}C_4$ isotopic species of prolinol **II**.

J'	*K'*$_{-1}$	*K'*$_{+1}$	*F*	←	*J"*	*K"*$_{-1}$	*K"*$_{+1}$	*F"*	ν_{obs} /MHz	ν_{obs}- ν_{calc}/MHz
1	0	1	1	←	0	0	0	1	3451.8896	-0.0002
1	0	1	2	←	0	0	0	1	3452.3047	-0.0026
1	0	1	0	←	0	0	0	1	3452.9320	-0.0016
2	1	2	2	←	1	1	1	2	6593.1230	-0.0166
2	1	2	3	←	1	1	1	2	6593.5078	-0.0023
2	1	2	1	←	1	1	1	0	6593.8114	-0.0122
2	0	2	1	←	1	0	1	0	6882.9996	-0.0122
2	0	2	3	←	1	0	1	2	6883.3927	0.0098
2	0	2	1	←	1	0	1	1	6884.0525	-0.0033
2	1	1	2	←	1	1	0	1	7215.1749	-0.0018
2	1	1	1	←	1	1	0	1	7215.2990	0.0025
2	1	1	3	←	1	1	0	2	7215.5902	-0.0092
2	1	1	1	←	1	1	0	0	7216.1556	-0.0052
3	1	3	3	←	2	1	2	3	9876.8541	-0.0006
3	1	3	3	←	2	1	2	2	9877.2238	-0.0013
3	1	3	4	←	2	1	2	3	9877.3624	0.0039
3	1	3	2	←	2	1	2	2	9877.9041	-0.0013
3	0	3	3	←	2	0	2	3	10272.3794	-0.0018
3	0	3	3	←	2	0	2	2	10272.8695	0.0082
3	0	3	4	←	2	0	2	3	10272.8695	0.0082
3	0	3	2	←	2	0	2	2	10273.5293	0.0213
3	2	2	3	←	2	2	1	2	10356.3652	0.0011

3	2	2	4	←	2	2	1	3	10356.8106	-0.0008
3	2	2	2	←	2	2	1	1	10357.0589	-0.0011
3	2	1	3	←	2	2	0	2	10440.1886	0.0118
3	2	1	4	←	2	2	0	3	10440.6079	-0.0001
3	2	1	3	←	2	2	0	3	10440.6079	-0.0001
3	2	1	2	←	2	2	0	1	10440.8524	0.0023
3	1	2	3	←	2	1	1	2	10809.4801	-0.0050
3	1	2	4	←	2	1	1	3	10809.6108	-0.0058
3	1	2	2	←	2	1	1	1	10809.6108	-0.0058
3	1	2	2	←	2	1	1	2	10809.7634	0.0085
4	1	4	4	←	3	1	3	4	13146.5316	0.0083
4	1	4	5	←	3	1	3	4	13147.1107	0.0193
4	1	4	3	←	3	1	3	3	13147.7389	-0.0025
4	0	4	4	←	3	0	3	4	13602.7990	-0.0120
4	0	4	3	←	3	0	3	2	13603.2966	-0.0079
4	0	4	4	←	3	0	3	3	13603.2966	-0.0079
4	0	4	3	←	3	0	3	3	13603.9604	-0.0103
4	2	3	4	←	3	2	2	3	13792.2988	-0.0016
4	2	3	5	←	3	2	2	4	13792.4699	-0.0234
4	2	2	4	←	3	2	1	3	13997.7752	0.0108
4	2	2	5	←	3	2	1	4	13997.9197	-0.0135
4	2	2	3	←	3	2	1	3	13997.9197	-0.0135
4	1	3	5	←	3	1	2	4	14385.9337	0.0190
5	0	5	5	←	4	0	4	4	16865.2284	0.0086
5	0	5	4	←	4	0	4	3	16865.2284	0.0086
5	0	5	6	←	4	0	4	5	16865.2284	0.0086

Table S28. Measured frequencies (ν_{obs}) and residuals ($\nu_{obs} - \nu_{cal}$) of the rotational transitions of the ^{13}**C**$_5$ isotopic species of prolinol II.

J'	*K'*$_{-1}$	*K'*$_{+1}$	*F*	←	*J''*	*K''*$_{-1}$	*K''*$_{+1}$	*F''*	ν_{obs} /MHz	ν_{obs}- ν_{calc}/MHz
1	0	1	1	←	0	0	0	1	3467.6534	-0.0040
1	0	1	2	←	0	0	0	1	3468.0553	-0.0029
1	0	1	0	←	0	0	0	1	3468.6677	0.0083
2	1	2	3	←	1	1	1	2	6631.9599	-0.0043
2	1	2	1	←	1	1	1	1	6632.1100	-0.0080
2	1	2	1	←	1	1	1	0	6632.2503	-0.0007
2	0	2	2	←	1	0	1	2	6915.7404	0.0069
2	0	2	1	←	1	0	1	0	6915.7404	0.0069
2	0	2	3	←	1	0	1	2	6916.1389	0.0127
2	0	2	1	←	1	0	1	2	6916.3652	-0.0065
2	0	2	1	←	1	0	1	1	6916.7672	-0.0053
2	1	1	2	←	1	1	0	1	7239.7539	0.0053
2	1	1	2	←	1	1	0	2	7240.1498	0.0052
2	1	1	3	←	1	1	0	2	7240.1498	0.0052
2	1	1	1	←	1	1	0	0	7240.7006	-0.0057
3	1	3	3	←	2	1	2	3	9935.2739	-0.0022
3	1	3	3	←	2	1	2	2	9935.6516	0.0031
3	1	3	4	←	2	1	2	3	9935.7867	0.0092
3	1	3	2	←	2	1	2	2	9936.3272	0.0018
3	0	3	3	←	2	0	2	3	10324.5256	0.0001
3	0	3	4	←	2	0	2	3	10325.0137	0.0107
3	0	3	2	←	2	0	2	2	10325.6110	-0.0009
3	2	2	3	←	2	2	1	2	10403.6397	0.0006
3	2	2	4	←	2	2	1	3	10404.0673	-0.0013
3	2	2	2	←	2	2	1	1	10404.3058	-0.0013
3	2	1	2	←	2	2	0	2	10482.5905	0.0094
3	2	1	3	←	2	2	0	2	10482.5905	0.0094
3	2	1	4	←	2	2	0	3	10482.9935	-0.0048

3	2	1	3	←	2	2	0	3	10482.9935	-0.0048
3	2	1	2	←	2	2	0	1	10483.2295	-0.0006
3	1	2	3	←	2	1	1	2	10847.1484	0.0043
3	1	2	4	←	2	1	1	3	10847.2627	-0.0084
3	1	2	2	←	2	1	1	1	10847.2627	-0.0084
4	1	4	4	←	3	1	3	4	13225.6750	-0.0036
4	1	4	4	←	3	1	3	3	13226.1513	-0.0287
4	1	4	5	←	3	1	3	4	13226.2691	0.0265
4	1	4	3	←	3	1	3	3	13226.8901	0.0010
4	0	4	4	←	3	0	3	4	13677.5095	-0.0027
4	0	4	3	←	3	0	3	2	13677.9854	-0.0047
4	0	4	5	←	3	0	3	4	13677.9854	-0.0398
4	0	4	3	←	3	0	3	3	13678.6482	0.0136
4	2	3	4	←	3	2	2	3	13856.2886	0.0035
4	2	3	5	←	3	2	2	4	13856.4579	-0.0125
5	0	5	6	←	4	0	4	5	16965.5420	0.0007

Table S29. Measured frequencies (ν_{obs}) and residuals ($\nu_{obs} - \nu_{cal}$) of the rotational transitions of the **$^{13}C_6$** isotopic species of prolinol **II**.

J'	*K'*$_{-1}$	*K'*$_{+1}$	*F*	←	*J"*	*K"*$_{-1}$	*K"*$_{+1}$	*F"*	ν_{obs} /MHz	ν_{obs}- ν_{calc}/MHz
1	0	1	1		0	0	0	1	3443.3005	-0.0035
1	0	1	2		0	0	0	1	3443.6997	-0.0004
1	0	1	0		0	0	0	1	3444.2920	-0.0024
2	1	2	2		1	1	1	1	6585.3321	-0.0185
2	1	2	3		1	1	1	2	6585.7669	-0.0045
2	1	2	1		1	1	1	1	6585.9171	-0.0092
2	1	2	1		1	1	1	0	6586.0483	-0.0048
2	0	2	2		1	0	1	2	6867.4091	0.0165
2	0	2	1		1	0	1	0	6867.4091	0.0165
2	0	2	3		1	0	1	2	6867.7959	0.0152
2	0	2	1		1	0	1	1	6868.4197	0.0000
2	1	1	2		1	1	0	1	7188.5199	0.0047
2	1	1	2		1	1	0	2	7188.9109	0.0041
2	1	1	3		1	1	0	2	7188.9109	0.0041
2	1	1	1		1	1	0	0	7189.4572	-0.0062
3	1	3	3		2	1	2	3	9866.2085	-0.0032
3	1	3	3		2	1	2	2	9866.5861	0.0043
3	1	3	4		2	1	2	3	9866.7184	0.0090
3	1	3	2		2	1	2	2	9867.2509	-0.0028
3	0	3	3		2	0	2	3	10252.9100	-0.0055
3	0	3	4		2	0	2	3	10253.3959	0.0085
3	0	3	2		2	0	2	2	10253.9939	0.0044
3	2	2	3		2	2	1	2	10330.5783	0.0072
3	2	2	4		2	2	1	3	10330.9947	-0.0009
3	2	2	2		2	2	1	1	10331.2326	0.0012
3	2	1	2		2	2	0	2	10408.0588	0.0024
3	2	1	3		2	2	0	2	10408.0588	0.0024
3	2	1	4		2	2	0	3	10408.4647	-0.0040
3	2	1	3		2	2	0	3	10408.4647	-0.0040
3	2	1	2		2	2	0	1	10408.6954	-0.0024
3	1	2	3		2	1	1	2	10770.5340	0.0011
3	1	2	4		2	1	1	3	10770.6460	-0.0032
4	1	4	4		3	1	3	4	13133.9752	0.0033
4	1	4	4		3	1	3	3	13134.4422	-0.0274
4	1	4	5		3	1	3	4	13134.5596	0.0280
4	1	4	3		3	1	3	3	13135.1825	0.0093
4	0	4	4		3	0	3	4	13583.5980	0.0012
4	0	4	4		3	0	3	3	13584.0608	-0.0080

4	0	4	5	3	0	3	4	13584.0608	-0.0434
4	0	4	3	3	0	3	3	13584.7144	0.0077
4	2	3	4	3	2	2	3	13759.1463	0.0007
4	2	3	5	3	2	2	4	13759.3161	-0.0126

Table S30. Measured frequencies (ν_{obs}) and residuals ($\nu_{obs} - \nu_{cal}$) of the rotational transitions of the ^{15}**N** isotopic species of prolinol II.

J'	*K'*$_{-1}$	*K'*$_{+1}$	←	*J"*	*K"*$_{-1}$	*K"*$_{+1}$	ν_{obs} /MHz	ν_{obs}- ν_{calc}/MHz
1	0	1	←	0	0	0	3464.7945	-0.0096
2	1	2	←	1	1	1	6620.3999	-0.0078
2	0	2	←	1	0	1	6908.7953	-0.0027
2	1	1	←	1	1	0	7238.7998	0.0015
3	1	3	←	2	1	2	9917.9912	0.0014
3	0	3	←	2	0	2	10311.7861	-0.0054
3	2	2	←	2	2	1	10394.4061	-0.0051
3	2	1	←	2	2	0	10476.9744	0.0022
3	1	2	←	2	1	1	10844.6961	0.0021
4	1	4	←	3	1	3	13201.6244	0.0051
4	0	4	←	3	0	3	13656.5571	0.0001
4	2	3	←	3	2	2	13842.9570	0.0041
4	1	3	←	3	1	2	14433.1403	0.0000
5	0	5	←	4	0	4	16933.5317	0.0010

Table S31. Measured frequencies (ν_{obs}) and residuals ($\nu_{obs} - \nu_{cal}$) of the rotational transitions of the ^{18}**O** isotopic species of prolinol II.

J'	*K'*$_{-1}$	*K'*$_{+1}$	*F*	←	*J"*	*K"*$_{-1}$	*K"*$_{+1}$	*F"*	ν_{obs} /MHz	ν_{obs}- ν_{calc}/MHz
1	0	1	1	←	0	0	0	1	3327.4187	-0.0037
1	0	1	2	←	0	0	0	1	3327.8396	0.0010
2	1	1	2	←	1	1	0	1	6936.7394	-0.0046
2	1	1	3	←	1	1	0	2	6937.1617	-0.0031
3	2	2	3	←	2	2	1	2	9982.9581	-0.0016
3	2	2	4	←	2	2	1	3	9983.4262	0.0205
3	2	2	2	←	2	2	1	1	9983.6404	-0.0129
3	1	2	3	←	2	1	1	2	10394.7877	0.0072
3	1	2	4	←	2	1	1	3	10394.8977	-0.0046
4	1	4	5	←	3	1	3	4	12716.3535	-0.0019
4	0	4	5	←	3	0	3	4	13147.9156	0.0001

8.2. Prolinol-H_2O

Table S32. Measured frequencies (ν_{obs}) and residuals ($\nu_{obs} - \nu_{cal}$) of the rotational transitions of isomer 1w1 of prolinol-H_2O.

J'	K'_{-1}	K'_{+1}	*F*	←	*J''*	K''_{-1}	K''_{+1}	*F''*	ν_{obs} /MHz	ν_{obs}- ν_{calc}/MHz
1	0	1	2	←	0	0	0	1	3029.8993	0.0049
1	0	1	0	←	0	0	0	1	3031.2185	-0.0109
1	1	1	0	←	0	0	0	1	3471.4494	-0.0079
1	1	1	2	←	0	0	0	1	3472.3263	-0.0054
1	1	1	1	←	0	0	0	1	3472.9142	-0.0005
1	1	0	0	←	0	0	0	1	3918.6612	0.0038
1	1	0	2	←	0	0	0	1	3919.1190	0.0014
1	1	0	1	←	0	0	0	1	3919.4198	-0.0045
2	1	2	2	←	1	1	1	1	5611.9931	0.0010
2	1	2	1	←	1	1	1	1	5612.4984	-0.0055
2	1	2	2	←	1	1	1	2	5612.5822	0.0071
2	1	2	3	←	1	1	1	2	5612.9067	0.0028
2	1	2	1	←	1	1	1	0	5613.9668	0.0054
2	0	2	1	←	1	0	1	0	5850.1413	-0.0012
2	0	2	2	←	1	0	1	2	5850.3293	0.0011
2	0	2	3	←	1	0	1	2	5851.0628	-0.0042
2	0	2	2	←	1	0	1	1	5851.2168	-0.0013
2	0	2	1	←	1	0	1	1	5852.3685	0.0011
2	1	2	1	←	1	0	1	0	6054.1959	0.0068
2	1	2	3	←	1	0	1	2	6055.3398	-0.0013
2	1	2	2	←	1	0	1	1	6055.9032	0.0009
2	1	2	1	←	1	0	1	1	6056.4271	0.0130
2	1	1	2	←	1	1	0	1	6505.4697	0.0029
2	1	1	2	←	1	1	0	2	6505.7791	0.0056
2	1	1	3	←	1	1	0	2	6506.4042	-0.0001
2	1	1	1	←	1	1	0	1	6506.4042	-0.0001
2	1	1	1	←	1	1	0	0	6507.2078	0.0021
2	1	1	1	←	1	0	1	0	7394.6366	0.0030
2	1	1	2	←	1	0	1	2	7394.9976	0.0009
2	1	1	3	←	1	0	1	2	7395.6235	0.0021
2	1	1	2	←	1	0	1	1	7395.8852	-0.0013
2	1	1	1	←	1	0	1	1	7396.8623	0.0038
2	2	1	3	←	1	1	0	2	7834.0823	-0.0002
2	2	1	2	←	1	1	0	1	7834.7307	0.0015
2	2	1	2	←	1	1	0	2	7835.0230	-0.0130
2	2	0	1	←	1	1	0	1	8041.8365	-0.0019
2	2	0	3	←	1	1	0	2	8042.5486	-0.0067
2	2	0	2	←	1	1	0	1	8042.9840	-0.0034
2	2	0	2	←	1	1	0	2	8043.2924	-0.0017
3	1	2	4	←	2	2	0	3	8043.5879	-0.0026
3	0	3	3	←	2	1	2	2	8241.5156	0.0066
3	0	3	4	←	2	1	2	3	8241.8052	-0.0142
2	2	1	1	←	1	1	1	1	8279.7580	0.0023
2	2	1	1	←	1	1	1	2	8280.3256	-0.0131
2	2	1	3	←	1	1	1	2	8280.8696	0.0012
2	2	1	1	←	1	1	1	0	8281.2395	0.0086
2	2	1	2	←	1	1	1	1	8281.2395	0.0086
2	2	1	2	←	1	1	1	2	8281.8228	0.0009
3	1	3	3	←	2	1	2	3	8308.1935	0.0007
3	1	3	3	←	2	1	2	2	8308.5198	-0.0018
3	1	3	4	←	2	1	2	3	8308.7274	-0.0008
3	1	3	2	←	2	1	2	1	8308.7274	-0.0008
3	1	3	2	←	2	1	2	2	8309.2406	-0.0021
3	0	3	3	←	2	0	2	3	8445.4625	0.0080
3	0	3	2	←	2	0	2	1	8445.9002	-0.0067
3	0	3	4	←	2	0	2	3	8446.0896	-0.0039
3	0	3	2	←	2	0	2	2	8447.0573	0.0012
2	2	0	3	←	1	1	1	2	8489.3452	0.0040

2	2	0	2	←	1	1	1	2	8490.0782	-0.0017
3	1	3	2	←	2	0	2	1	8512.7852	0.0076
3	1	3	4	←	2	0	2	3	8512.9974	-0.0036
3	1	3	3	←	2	0	2	2	8513.2119	0.0061
3	2	2	3	←	2	2	1	2	9088.4184	0.0172
3	2	2	4	←	2	2	1	3	9089.3691	0.0145
3	2	2	2	←	2	2	1	1	9089.8923	0.0078
3	1	2	3	←	2	1	1	3	9578.9870	0.0070
3	1	2	2	←	2	1	1	1	9579.7101	-0.0126
3	1	2	4	←	2	1	1	3	9579.7101	-0.0126
3	2	1	3	←	2	2	0	2	9731.3749	-0.0078
3	2	1	4	←	2	2	0	3	9732.4688	-0.0023
3	2	1	2	←	2	2	0	1	9732.9979	-0.0060
4	0	4	3	←	3	1	3	2	10922.7770	0.0007
4	0	4	5	←	3	1	3	4	10922.7770	0.0007
4	1	4	5	←	3	1	3	4	10940.7452	-0.0126
4	0	4	3	←	3	0	3	2	10989.6876	0.0044
4	0	4	5	←	3	0	3	4	10989.6876	0.0044
4	0	4	4	←	3	0	3	3	10989.6876	0.0044
3	1	2	3	←	2	0	2	3	11123.5247	-0.0097
3	1	2	4	←	2	0	2	3	11124.2991	-0.0029
3	1	2	2	←	2	0	2	2	11125.2998	-0.0097
3	2	1	2	←	2	1	1	1	11268.3940	-0.0095
3	2	1	4	←	2	1	1	3	11268.6186	-0.0096
3	2	1	3	←	2	1	1	2	11268.8948	-0.0084
3	2	1	2	←	2	1	1	2	11269.3647	-0.0107
3	2	2	2	←	2	1	2	1	11757.1496	0.0133
3	2	2	4	←	2	1	2	3	11757.3313	0.0121
3	2	2	3	←	2	1	2	2	11757.6628	0.0149
4	2	3	4	←	3	2	2	3	11947.4127	0.0084
4	2	3	5	←	3	2	2	4	11947.7709	-0.0096
4	2	3	3	←	3	2	2	2	11947.7709	-0.0096
4	1	3	5	←	3	1	2	4	12385.9660	-0.0018
4	1	3	3	←	3	1	2	3	12386.9058	-0.0065
3	3	0	2	←	2	2	0	2	12389.2335	-0.0134
3	3	0	4	←	2	2	0	3	12390.3774	-0.0033
3	3	0	2	←	2	2	0	1	12390.3774	-0.0033
3	3	0	3	←	2	2	0	2	12390.7453	0.0018
3	3	0	3	←	2	2	0	3	12391.4919	0.0097
3	3	1	2	←	2	2	1	2	12528.2936	0.0037
3	3	1	4	←	2	2	1	3	12529.6553	-0.0017
3	3	1	2	←	2	2	1	1	12529.7736	0.0006
3	3	1	3	←	2	2	1	2	12529.8924	0.0069
3	3	1	3	←	2	2	1	3	12530.8480	0.0090
3	3	0	4	←	2	2	1	3	12598.8511	0.0047
3	3	0	2	←	2	2	1	1	12598.9936	-0.0025
3	3	0	3	←	2	2	1	2	12598.9936	-0.0025
5	1	5	5	←	4	1	4	4	13540.0502	-0.0068
5	1	5	4	←	4	1	4	3	13540.0502	-0.0068
5	1	5	6	←	4	1	4	5	13540.0502	-0.0068
5	0	5	6	←	4	0	4	5	13553.7061	-0.0055
5	1	5	6	←	4	0	4	5	13558.0391	-0.0061
5	2	4	6	←	4	2	3	5	14694.2663	0.0135
4	2	3	5	←	3	1	3	4	15396.3392	-0.0010
4	2	3	4	←	3	1	3	3	15396.5442	0.0136
4	3	1	5	←	3	2	1	4	15403.1964	0.0008
4	3	1	4	←	3	2	1	3	15403.5675	-0.0033
4	3	2	5	←	3	2	2	4	15843.8913	0.0013
4	3	2	4	←	3	2	2	3	15844.1547	0.0137
6	1	6	6	←	5	1	5	5	16127.2941	0.0000
6	1	6	7	←	5	1	5	6	16127.2941	0.0000
6	0	6	5	←	5	0	5	4	16130.6525	0.0051
6	0	6	6	←	5	0	5	5	16130.6525	0.0051
6	0	6	7	←	5	0	5	6	16130.6525	0.0051
4	4	0	5	←	3	3	0	4	16791.2221	-0.0130

4	4	0	4	←	3	3	0	3	16791.4757	0.0127
4	4	1	5	←	3	3	1	4	16841.6112	-0.0044
4	4	1	3	←	3	3	1	2	16841.6112	-0.0044
4	4	1	4	←	3	3	1	3	16841.7478	-0.0166

Table S33. Measured frequencies (ν_{obs}) and residuals ($\nu_{obs} - \nu_{cal}$) of the rotational transitions of isomer 1w2 of prolinol-H_2O.

J'	K'_{-1}	K'_{+1}	F	←	J''	K''_{-1}	K''_{+1}	F''	ν_{obs} /MHz	ν_{obs}- ν_{calc}/MHz
1	0	1	1	←	0	0	0	1	2401.0045	-0.0011
1	0	1	2	←	0	0	0	1	2401.9410	-0.0001
1	0	1	0	←	0	0	0	1	2403.3438	-0.0007
3	1	2	4	←	3	0	3	4	2935.6059	-0.0132
3	1	2	3	←	3	0	3	3	2935.7587	0.0046
2	0	2	2	←	1	1	1	1	3422.4997	-0.0029
2	0	2	2	←	1	1	1	2	3423.0160	-0.0035
2	0	2	3	←	1	1	1	2	3423.9787	-0.0096
2	0	2	1	←	1	1	1	1	3423.9787	-0.0096
4	2	2	3	←	4	1	3	3	3701.9177	0.0041
4	2	2	5	←	4	1	3	5	3702.0117	0.0014
4	2	2	4	←	4	1	3	4	3702.3892	0.0023
1	1	1	0	←	0	0	0	1	3708.9768	0.0012
1	1	1	2	←	0	0	0	1	3709.7496	-0.0013
1	1	1	1	←	0	0	0	1	3710.2692	0.0013
3	2	1	2	←	3	1	2	2	3745.7410	-0.0011
3	2	1	4	←	3	1	2	4	3746.0001	0.0007
3	2	1	3	←	3	1	2	3	3746.7344	-0.0002
2	2	1	3	←	2	1	1	3	3923.7923	-0.0010
2	2	0	1	←	2	1	1	1	3994.7855	0.0017
2	2	0	3	←	2	1	1	3	3995.6215	-0.0068
2	2	0	2	←	2	1	1	2	3997.1497	0.0017
5	2	3	4	←	5	1	4	4	3997.9631	0.0065
5	2	3	6	←	5	1	4	6	3997.9631	0.0065
5	2	3	5	←	5	1	4	5	3998.1407	-0.0055
1	1	0	0	←	0	0	0	1	4090.2436	-0.0033
1	1	0	2	←	0	0	0	1	4090.8735	-0.0011
1	1	0	1	←	0	0	0	1	4091.3022	0.0093
4	1	3	5	←	4	0	4	5	4129.2559	0.0070
4	1	3	4	←	4	0	4	4	4129.2559	0.0070
2	1	2	2	←	1	1	1	1	4421.6723	-0.0030
2	1	2	2	←	1	1	1	2	4422.1910	-0.0011
2	1	2	1	←	1	1	1	1	4422.3726	-0.0006
2	1	2	3	←	1	1	1	2	4422.6406	0.0001
2	1	2	1	←	1	1	1	2	4422.8977	0.0077
2	1	2	1	←	1	1	1	0	4423.6708	0.0054
2	0	2	2	←	1	0	1	2	4730.8290	-0.0004
2	0	2	1	←	1	0	1	0	4730.9289	0.0004
2	0	2	3	←	1	0	1	2	4731.7966	0.0014
2	0	2	1	←	1	0	1	1	4733.2683	0.0010
2	2	1	1	←	2	1	2	1	5066.3204	-0.0086
2	2	1	2	←	2	1	2	2	5068.5875	0.0015
2	1	1	2	←	1	1	0	1	5183.8866	-0.0002
2	1	1	2	←	1	1	0	2	5184.3050	-0.0001
2	1	1	1	←	1	1	0	1	5184.7505	0.0018
2	1	1	3	←	1	1	0	2	5184.8591	0.0001
2	1	1	1	←	1	1	0	0	5185.7947	-0.0001
3	2	2	2	←	3	1	3	2	5680.0071	-0.0056
3	2	2	4	←	3	1	3	4	5680.2761	0.0133
3	2	2	3	←	3	1	3	3	5680.9784	0.0009
2	1	2	1	←	1	0	1	0	5729.2925	-0.0040
2	1	2	2	←	1	0	1	2	5729.9978	-0.0042
2	1	2	3	←	1	0	1	2	5730.4492	-0.0012

2	1	2	2	←	1	0	1	1	5730.9394	0.0019
2	1	2	1	←	1	0	1	1	5731.6307	-0.0047
3	0	3	3	←	2	1	2	2	5934.2902	-0.0007
3	0	3	4	←	2	1	2	3	5934.7964	-0.0009
3	0	3	2	←	2	1	2	1	5934.8817	-0.0005
3	0	3	2	←	2	1	2	2	5935.5763	-0.0037
5	3	2	4	←	5	2	3	4	6319.9385	-0.0002
5	3	2	6	←	5	2	3	6	6320.0554	-0.0042
5	3	2	5	←	5	2	3	5	6320.6436	-0.0096
3	2	2	2	←	3	0	3	2	6337.5300	-0.0068
3	2	2	4	←	3	0	3	4	6337.8707	-0.0003
3	2	2	3	←	3	0	3	3	6338.8287	0.0029
4	2	3	3	←	4	1	4	3	6507.4458	0.0073
4	2	3	5	←	4	1	4	5	6507.5487	0.0052
4	2	3	4	←	4	1	4	4	6507.9524	0.0006
3	1	3	3	←	2	1	2	3	6591.6903	-0.0006
3	1	3	3	←	2	1	2	2	6592.1387	-0.0005
3	1	3	4	←	2	1	2	3	6592.4058	0.0001
3	1	3	2	←	2	1	2	1	6592.4058	0.0001
3	1	3	2	←	2	1	2	2	6593.0999	-0.0043
4	1	3	4	←	3	2	2	3	6794.1538	-0.0034
4	1	3	5	←	3	2	2	4	6795.0740	-0.0019
4	1	3	3	←	3	2	2	2	6795.3098	-0.0023
4	3	1	5	←	4	2	2	5	6824.7475	-0.0037
4	3	1	4	←	4	2	2	4	6825.6283	0.0037
2	1	1	1	←	1	0	1	0	6872.7001	0.0030
2	1	1	2	←	1	0	1	2	6873.2420	0.0034
2	1	1	3	←	1	0	1	2	6873.7921	-0.0004
2	1	1	2	←	1	0	1	1	6874.1770	0.0030
2	1	1	1	←	1	0	1	1	6875.0353	-0.0007
4	2	3	3	←	4	0	4	3	6884.8393	-0.0010
4	2	3	5	←	4	0	4	5	6884.9757	0.0016
4	2	3	4	←	4	0	4	4	6885.4941	-0.0005
3	0	3	3	←	2	0	2	3	6932.4959	-0.0019
3	0	3	2	←	2	0	2	1	6933.2494	-0.0007
3	0	3	4	←	2	0	2	3	6933.4557	-0.0013
3	0	3	3	←	2	0	2	2	6933.4557	-0.0013
3	0	3	2	←	2	0	2	2	6934.7526	-0.0002
3	3	0	2	←	3	2	1	2	7198.3778	-0.0034
3	3	0	4	←	3	2	1	4	7198.8554	-0.0053
3	3	0	3	←	3	2	1	3	7200.2293	-0.0009
3	2	2	3	←	2	2	1	2	7204.5296	-0.0011
3	2	2	4	←	2	2	1	3	7205.5331	0.0002
3	2	2	2	←	2	2	1	1	7206.0892	-0.0007
3	2	1	3	←	2	2	0	2	7476.3937	-0.0013
3	2	1	2	←	2	2	0	2	7476.5080	-0.0015
3	2	1	3	←	2	2	0	3	7477.3579	-0.0028
3	2	1	4	←	2	2	0	3	7477.4447	-0.0006
3	2	1	2	←	2	2	0	1	7478.0103	-0.0014
3	3	1	2	←	3	2	2	2	7533.5098	-0.0023
3	3	1	4	←	3	2	2	4	7533.9590	-0.0044
3	3	1	3	←	3	2	2	3	7535.2518	-0.0006
5	2	4	6	←	5	1	5	6	7539.5970	0.0008
5	2	4	5	←	5	1	5	5	7539.8604	0.0099
3	1	3	3	←	2	0	2	3	7590.3479	0.0017
3	1	3	2	←	2	0	2	1	7590.7763	0.0020
3	1	3	4	←	2	0	2	3	7591.0606	-0.0001
3	1	3	2	←	2	0	2	3	7591.3123	0.0004
3	1	3	3	←	2	0	2	2	7591.3123	0.0004
3	1	3	2	←	2	0	2	2	7592.2793	0.0024
4	3	2	3	←	4	2	3	3	7711.5818	-0.0035
4	3	2	5	←	4	2	3	5	7711.7844	0.0025
4	3	2	4	←	4	2	3	4	7712.5389	-0.0077
3	1	2	3	←	2	1	1	3	7726.2527	-0.0018
3	1	2	3	←	2	1	1	2	7726.8075	-0.0009

3	1	2	2	←	2	1	1	1	7727.0782	0.0106
3	1	2	4	←	2	1	1	3	7727.0782	0.0106
3	1	2	2	←	2	1	1	2	7727.9152	-0.0001
4	0	4	4	←	3	1	3	3	8345.8745	-0.0012
4	0	4	3	←	3	1	3	2	8346.0942	0.0002
4	0	4	5	←	3	1	3	4	8346.0942	0.0002
4	1	4	4	←	3	1	3	4	8722.7039	0.0000
4	1	4	4	←	3	1	3	3	8723.4187	0.0002
4	1	4	5	←	3	1	3	4	8723.5516	0.0235
4	1	4	3	←	3	1	3	3	8724.4617	0.0068
4	0	4	4	←	3	0	3	4	9002.7691	-0.0002
4	0	4	5	←	3	0	3	4	9003.7137	0.0001
4	0	4	4	←	3	0	3	3	9003.7137	0.0001
4	0	4	3	←	3	0	3	3	9004.9035	0.0021
2	2	1	1	←	1	1	0	1	9107.6792	0.0020
2	2	1	3	←	1	1	0	2	9108.6620	-0.0039
2	2	1	1	←	1	1	0	0	9108.6620	-0.0039
2	2	1	2	←	1	1	0	1	9109.2248	-0.0114
2	2	1	2	←	1	1	0	2	9109.6506	-0.0039
2	2	0	1	←	1	1	0	1	9179.5210	-0.0115
2	2	0	3	←	1	1	0	2	9180.4900	0.0027
2	2	0	2	←	1	1	0	1	9181.0395	0.0048
2	2	0	2	←	1	1	0	2	9181.4595	0.0065
4	1	4	3	←	3	0	3	2	9381.0015	-0.0125
4	1	4	5	←	3	0	3	4	9381.1388	0.0024
4	1	4	4	←	3	0	3	3	9381.2666	-0.0003
2	2	1	1	←	1	1	1	1	9488.7011	-0.0011
2	2	1	3	←	1	1	1	2	9489.7735	-0.0024
2	2	1	1	←	1	1	1	0	9489.9967	0.0022
2	2	1	2	←	1	1	1	1	9490.2562	-0.0051
2	2	1	2	←	1	1	1	2	9490.7836	0.0056
4	2	3	4	←	3	2	2	3	9550.3912	-0.0016
4	2	3	5	←	3	2	2	4	9550.8003	-0.0085
4	2	3	3	←	3	2	2	2	9550.9181	0.0023
2	2	0	1	←	1	1	1	1	9560.5532	-0.0043
2	2	0	3	←	1	1	1	2	9561.6114	0.0005
2	2	0	1	←	1	1	1	0	9561.8502	0.0004
2	2	0	2	←	1	1	1	1	9562.0581	-0.0017
2	2	0	2	←	1	1	1	2	9562.5773	0.0006
4	3	2	4	←	3	3	1	3	9727.6882	0.0012
4	3	2	5	←	3	3	1	4	9728.6268	-0.0006
4	3	2	4	←	3	3	1	4	9728.9899	0.0019
4	3	2	3	←	3	3	1	2	9728.9899	0.0019
4	3	1	4	←	3	3	0	3	9778.2755	-0.0002
4	3	1	5	←	3	3	0	4	9779.2292	-0.0001
4	3	1	4	←	3	3	0	4	9779.5911	-0.0001
4	3	1	3	←	3	3	0	2	9779.5911	-0.0001
3	1	2	2	←	2	0	2	1	9868.8189	-0.0030
3	1	2	4	←	2	0	2	3	9869.0760	0.0043
3	1	2	3	←	2	0	2	2	9869.2117	-0.0059
4	2	2	4	←	3	2	1	3	10152.8787	-0.0025
4	2	2	5	←	3	2	1	4	10153.3312	-0.0076
4	2	2	3	←	3	2	1	2	10153.4451	-0.0035
4	1	3	4	←	3	1	2	4	10196.4078	-0.0013
4	1	3	4	←	3	1	2	3	10197.2075	-0.0213
4	1	3	5	←	3	1	2	4	10197.3382	0.0104
4	1	3	3	←	3	1	2	3	10198.3783	-0.0056
5	1	5	5	←	4	1	4	5	10816.7918	0.0005
5	1	5	5	←	4	1	4	4	10817.5957	-0.0198
5	1	5	6	←	4	1	4	5	10817.6932	0.0237
5	1	5	4	←	4	1	4	4	10818.6653	-0.0076
5	0	5	5	←	4	0	4	5	10999.5386	0.0080
5	0	5	6	←	4	0	4	5	11000.4618	-0.0003
5	0	5	5	←	4	0	4	4	11000.4618	-0.0003
5	0	5	4	←	4	0	4	4	11001.5796	-0.0042

3	2	2	2	←	2	1	1	1	11129.0194	0.0011
3	2	2	4	←	2	1	1	3	11129.3283	0.0022
3	2	2	3	←	2	1	1	2	11129.8813	0.0012
5	1	5	4	←	4	0	4	3	11195.1028	-0.0001
5	1	5	6	←	4	0	4	5	11195.1028	-0.0001
5	1	5	5	←	4	0	4	4	11195.1028	-0.0001
3	2	1	2	←	2	1	1	1	11472.7982	0.0027
3	2	1	4	←	2	1	1	3	11473.0734	-0.0003
3	2	1	3	←	2	1	1	2	11473.5418	-0.0011
5	2	4	5	←	4	2	3	4	11849.5147	0.0005
5	2	4	6	←	4	2	3	5	11849.7260	-0.0043
5	2	4	4	←	4	2	3	3	11849.7260	-0.0043
5	4	2	5	←	4	4	1	4	12167.1307	-0.0048
5	4	2	6	←	4	4	1	5	12167.9925	0.0020
5	4	2	4	←	4	4	1	3	12168.2447	0.0010
5	4	1	5	←	4	4	0	4	12173.9082	0.0028
5	4	1	6	←	4	4	0	5	12174.7639	0.0016
5	4	1	4	←	4	4	0	3	12175.0181	0.0022
5	3	3	5	←	4	3	2	4	12178.3849	-0.0010
5	3	3	6	←	4	3	2	5	12178.8655	-0.0057
5	3	3	4	←	4	3	2	3	12178.9980	0.0093
3	2	2	2	←	2	1	2	1	12272.4213	0.0024
3	2	2	4	←	2	1	2	3	12272.6645	-0.0038
3	2	2	3	←	2	1	2	2	12273.1169	0.0003
5	3	2	5	←	4	3	1	4	12346.0935	-0.0008
5	3	2	6	←	4	3	1	5	12346.5990	-0.0065
5	3	2	4	←	4	3	1	3	12346.7328	0.0055
5	1	4	5	←	4	1	3	5	12554.3922	0.0046
5	1	4	4	←	4	1	3	4	12556.4456	-0.0027
3	2	1	2	←	2	1	2	1	12616.1904	-0.0057
3	2	1	4	←	2	1	2	3	12616.4116	-0.0042
3	2	1	3	←	2	1	2	2	12616.7771	-0.0024
6	0	6	7	←	5	1	5	6	12789.1544	0.0164
5	2	3	5	←	4	2	2	4	12851.0647	-0.0010
5	2	3	6	←	4	2	2	5	12851.3010	0.0040
5	2	3	6	←	4	2	2	5	12851.3010	0.0040
6	1	6	6	←	5	1	5	5	12882.3149	-0.0070
6	1	6	5	←	5	1	5	4	12882.3149	-0.0070
6	1	6	7	←	5	1	5	6	12882.3149	-0.0070
4	2	3	3	←	3	1	2	2	12952.8834	0.0027
4	2	3	5	←	3	1	2	4	12953.0594	-0.0013
4	2	3	4	←	3	1	2	3	12953.4613	-0.0032
6	0	6	6	←	5	0	5	5	12983.7835	0.0041
6	0	6	7	←	5	0	5	6	12983.7835	0.0041
6	1	6	6	←	5	0	5	5	13077.0136	0.0135
3	2	1	2	←	2	0	2	1	13614.5707	0.0066
3	2	1	4	←	2	0	2	3	13615.0699	-0.0011
3	2	1	3	←	2	0	2	2	13615.9454	-0.0068
4	2	2	3	←	3	1	2	2	13899.1957	0.0050
4	2	2	5	←	3	1	2	4	13899.3315	-0.0067
4	2	2	4	←	3	1	2	3	13899.6153	-0.0004
6	2	5	5	←	5	2	4	4	14095.0564	-0.0065
6	2	5	7	←	5	2	4	6	14095.0564	-0.0065
3	3	1	2	←	2	2	0	2	14666.2636	0.0192
3	3	1	4	←	2	2	0	3	14667.6712	0.0099
3	3	1	3	←	2	2	0	2	14667.9829	-0.0017
3	3	1	3	←	2	2	0	3	14668.9481	-0.0023
3	3	0	4	←	2	2	0	3	14676.3278	0.0218
3	3	0	3	←	2	2	0	2	14676.6227	-0.0025
3	3	1	4	←	2	2	1	3	14739.4994	0.0031
3	3	1	3	←	2	2	1	2	14739.7835	0.0003
3	3	0	4	←	2	2	1	3	14748.1440	0.0029
3	3	0	3	←	2	2	1	2	14748.4182	-0.0055
6	1	5	7	←	5	1	4	6	14765.0026	0.0042
6	1	5	6	←	5	1	4	5	14765.0026	0.0042

7	0	7	7	←	6	1	6	6	14884.4459	0.0003
7	0	7	6	←	6	1	6	5	14884.4459	0.0003
7	1	7	7	←	6	1	6	6	14926.8881	0.0058
7	0	7	6	←	6	0	6	5	14977.6625	0.0065
7	0	7	7	←	6	0	6	6	14977.6625	0.0065
7	1	7	6	←	6	0	6	5	15020.0949	0.0026
7	1	7	7	←	6	0	6	6	15020.0949	0.0026
4	2	3	5	←	3	1	3	4	15231.0770	0.0054
4	2	3	4	←	3	1	3	3	15231.3610	-0.0093
7	1	6	6	←	6	1	5	5	16826.9671	-0.0063
7	1	6	7	←	6	1	5	6	16826.9671	-0.0063
4	3	2	5	←	3	2	1	4	16918.8354	-0.0079
4	3	2	4	←	3	2	1	3	16919.2831	0.0064
8	1	8	7	←	7	1	7	6	16959.3929	-0.0143
4	3	1	4	←	3	2	2	3	17322.1652	-0.0035

Table S34. Measured frequencies (ν_{obs}) and residuals ($\nu_{obs} - \nu_{cal}$) of the rotational transitions of isomer 1w9 of prolinol-H_2O.

J'	*K'*$_{-1}$	*K'*$_{+1}$	*F*	←	*J''*	*K''*$_{-1}$	*K''*$_{+1}$	*F''*	ν_{obs} /MHz	ν_{obs}- ν_{calc}/MHz
1	0	1	1	←	0	0	0	1	2501.5507	-0.0009
1	0	1	2	←	0	0	0	1	2501.7257	-0.0014
1	0	1	0	←	0	0	0	1	2501.9835	-0.0068
1	1	1	0	←	0	0	0	1	3560.3525	-0.0006
1	1	1	2	←	0	0	0	1	3561.3485	0.0015
1	1	1	1	←	0	0	0	1	3562.0082	-0.0014
2	1	2	1	←	1	1	1	1	4712.5762	0.0016
2	1	2	2	←	1	1	1	1	4713.3862	-0.0002
2	1	2	3	←	1	1	1	2	4713.5286	0.0016
2	1	2	2	←	1	1	1	2	4714.0483	-0.0007
2	1	2	1	←	1	1	1	0	4714.2317	0.0006
2	0	2	1	←	1	0	1	0	4951.3217	0.0045
2	0	2	2	←	1	0	1	2	4951.5564	0.0033
2	0	2	3	←	1	0	1	2	4951.5564	0.0033
2	0	2	1	←	1	0	1	2	4951.5564	0.0033
2	0	2	2	←	1	0	1	1	4951.6951	-0.0001
2	0	2	1	←	1	0	1	1	4951.7602	0.0044
2	1	1	2	←	1	1	0	2	5292.5902	-0.0007
2	1	1	1	←	1	1	0	0	5292.9615	-0.0030
2	1	1	2	←	1	1	0	1	5293.0749	-0.0033
2	1	1	3	←	1	1	0	2	5293.2995	-0.0015
2	1	1	1	←	1	1	0	1	5294.1826	-0.0001
2	1	2	1	←	1	0	1	0	5772.5945	0.0007
2	1	2	3	←	1	0	1	2	5773.1487	0.0018
2	1	2	2	←	1	0	1	1	5773.8419	-0.0025
3	0	3	3	←	2	1	2	2	6485.9346	-0.0013
3	0	3	4	←	2	1	2	3	6486.2923	-0.0032
3	0	3	2	←	2	1	2	1	6486.5276	-0.0010
3	1	3	2	←	2	1	2	2	7039.5598	0.0004
3	1	3	4	←	2	1	2	3	7040.2722	-0.0022
3	1	3	3	←	2	1	2	2	7040.2722	-0.0022
3	1	3	2	←	2	1	2	1	7040.3709	-0.0003
3	1	3	3	←	2	1	2	3	7040.8041	-0.0003
3	0	3	2	←	2	0	2	1	7307.8001	-0.0052
3	0	3	2	←	2	0	2	2	7307.8839	0.0016
3	0	3	4	←	2	0	2	3	7307.8839	0.0016
3	0	3	3	←	2	0	2	2	7308.0846	-0.0004
3	2	2	3	←	2	2	1	2	7504.9735	-0.0096
3	2	2	2	←	2	2	1	2	7504.9735	-0.0096
3	2	2	4	←	2	2	1	3	7505.1637	-0.0074
3	2	2	2	←	2	2	1	1	7505.2719	-0.0036
3	2	1	3	←	2	2	0	2	7701.7152	-0.0024

3	2	1	3	←	2	2	0	3	7701.7152	-0.0024
3	2	1	4	←	2	2	0	3	7702.1178	0.0083
3	2	1	2	←	2	2	0	3	7702.2559	-0.0002
3	2	1	2	←	2	2	0	1	7702.2559	-0.0002
3	1	3	2	←	2	0	2	1	7861.6476	-0.0003
3	1	3	4	←	2	0	2	3	7861.8575	0.0006
3	1	3	3	←	2	0	2	2	7862.4350	0.0034
3	1	2	3	←	2	1	1	3	7903.1237	-0.0036
3	1	2	2	←	2	1	1	1	7903.7140	-0.0023
3	1	2	3	←	2	1	1	2	7903.8554	0.0072
3	1	2	4	←	2	1	1	3	7903.8554	0.0072
3	1	2	2	←	2	1	1	2	7904.8194	-0.0014
4	0	4	4	←	3	1	3	3	9010.8632	0.0045
4	0	4	5	←	3	1	3	4	9011.0259	0.0008
4	0	4	5	←	3	0	3	4	9565.0190	0.0207
4	0	4	4	←	3	0	3	3	9565.2027	-0.0026
4	1	4	5	←	3	0	3	4	9892.4822	-0.0082
4	1	4	4	←	3	0	3	3	9892.9181	0.0053
4	2	3	5	←	3	2	2	4	9965.9796	-0.0045
4	2	3	3	←	3	2	2	2	9965.9796	-0.0045
4	3	2	4	←	3	3	1	3	10095.0009	-0.0073
4	3	2	4	←	3	3	1	4	10095.2517	-0.0152
4	3	2	5	←	3	3	1	4	10095.2517	-0.0152
4	3	2	3	←	3	3	1	2	10095.2517	-0.0152
4	3	1	5	←	3	3	0	4	10129.7961	0.0275
4	2	2	4	←	3	2	1	3	10406.2919	0.0162
4	2	2	5	←	3	2	1	4	10406.5364	-0.0036
4	2	2	3	←	3	2	1	2	10406.5364	-0.0036
4	1	3	5	←	3	1	2	4	10463.0516	-0.0008
5	1	5	6	←	4	1	4	5	11608.9723	-0.0095
5	1	5	4	←	4	1	4	3	11608.9723	-0.0095
5	0	5	4	←	4	0	4	3	11762.1065	0.0048
5	0	5	6	←	4	0	4	5	11762.1065	0.0048
5	0	5	5	←	4	0	4	4	11762.2712	-0.0019
5	1	4	4	←	4	1	3	3	12942.3169	-0.0063
5	1	4	6	←	4	1	3	5	12942.3169	-0.0063
5	1	4	5	←	4	1	3	4	12942.4695	-0.0023
6	0	6	7	←	5	1	5	6	13770.4519	-0.0022
6	0	6	6	←	5	1	5	5	13770.4519	-0.0022
6	0	6	5	←	5	1	5	4	13770.4519	-0.0022
6	1	6	7	←	5	1	5	6	13856.6719	-0.0047
6	1	6	5	←	5	1	5	4	13856.6719	-0.0047
6	0	6	5	←	5	0	5	4	13944.8039	0.0000
6	0	6	7	←	5	0	5	6	13944.8039	0.0000
6	0	6	6	←	5	0	5	5	13944.9313	0.0099
6	1	6	5	←	5	0	5	4	14031.0249	-0.0014
6	1	6	7	←	5	0	5	6	14031.0249	-0.0014

Table S35. Measured frequencies (ν_{obs}) and residuals ($\nu_{obs} - \nu_{cal}$) of the rotational transitions of isomer 1w12 of prolinol-H_2O.

J'	*K'*$_{-1}$	*K'*$_{+1}$	*F*	←	*J''*	*K''*$_{-1}$	*K''*$_{+1}$	*F''*	ν_{obs} /MHz	ν_{obs}- ν_{calc}/MHz
2	1	2	1	←	1	1	1	0	3848.0965	0.0039
2	1	2	2	←	1	1	1	2	3848.3879	-0.0017
2	1	2	3	←	1	1	1	2	3849.0076	0.0037
2	1	2	2	←	1	1	1	1	3849.2269	0.0022
2	1	2	1	←	1	1	1	1	3850.1892	0.0087
2	0	2	1	←	1	0	1	1	3902.1992	0.0047
2	0	2	3	←	1	0	1	2	3902.6016	-0.0014
2	0	2	2	←	1	0	1	1	3902.6016	-0.0014
2	0	2	1	←	1	0	1	0	3902.8526	-0.0034
2	0	2	2	←	1	0	1	2	3902.8526	-0.0034

2	1	1	1	←	1	1	0	1	3956.9567	-0.0028
2	1	1	3	←	1	1	0	2	3958.0245	-0.0053
2	1	1	2	←	1	1	0	1	3958.3606	-0.0034
2	1	1	1	←	1	1	0	0	3958.3606	-0.0034
2	1	1	2	←	1	1	0	2	3958.9185	-0.0060
3	1	3	3	←	2	1	2	3	5772.3309	0.0125
3	1	3	2	←	2	1	2	1	5772.7495	0.0032
3	1	3	4	←	2	1	2	3	5772.9047	-0.0017
3	1	3	3	←	2	1	2	2	5772.9047	-0.0017
3	1	3	2	←	2	1	2	2	5773.7012	-0.0009
3	0	3	3	←	2	0	2	2	5851.5341	0.0078
3	0	3	4	←	2	0	2	3	5851.5341	0.0078
3	0	3	2	←	2	0	2	1	5851.5929	-0.0005
3	0	3	3	←	2	0	2	3	5851.7788	-0.0004
3	2	2	2	←	2	2	1	1	5855.1484	-0.0068
3	2	2	4	←	2	2	1	3	5855.3075	-0.0036
3	2	2	3	←	2	2	1	2	5855.5874	-0.0042
3	2	1	2	←	2	2	0	1	5858.8879	-0.0043
3	2	1	4	←	2	2	0	3	5859.0509	-0.0032
3	2	1	3	←	2	2	0	2	5859.3608	-0.0015
3	1	2	2	←	2	1	1	2	5935.2581	-0.0111
3	1	2	4	←	2	1	1	3	5936.5060	-0.0062
3	1	2	3	←	2	1	1	2	5936.6167	-0.0152
3	1	2	2	←	2	1	1	1	5936.6167	-0.0152
3	1	2	3	←	2	1	1	3	5937.5058	-0.0012
4	1	4	5	←	3	1	3	4	7696.0269	-0.0030
4	1	4	4	←	3	1	3	3	7696.0269	-0.0030
4	1	4	3	←	3	1	3	4	7696.1560	-0.0149
4	1	4	3	←	3	1	3	3	7696.7421	0.0013
4	0	4	3	←	3	0	3	3	7797.3036	0.0016
4	0	4	3	←	3	0	3	4	7797.5567	0.0089
4	0	4	5	←	3	0	3	4	7797.5976	-0.0056
4	2	3	5	←	3	2	2	4	7806.3261	0.0089
4	2	3	4	←	3	2	2	3	7806.4307	-0.0005
4	2	2	5	←	3	2	1	4	7815.6724	0.0012
4	2	2	4	←	3	2	1	3	7815.8230	-0.0006
4	1	3	3	←	3	1	2	3	7912.9308	0.0092
4	1	3	5	←	3	1	2	4	7914.1903	0.0068
4	1	3	4	←	3	1	2	4	7915.2262	0.0044
5	1	5	4	←	4	1	4	3	9618.2040	0.0119
5	1	5	5	←	4	1	4	4	9618.2040	0.0119
5	1	5	6	←	4	1	4	5	9618.2040	0.0119
5	0	5	4	←	4	0	4	4	9739.6486	-0.0002
5	0	5	6	←	4	0	4	5	9739.8996	-0.0063
5	0	5	4	←	4	0	4	3	9739.8996	-0.0063
5	2	4	4	←	4	2	3	3	9756.6158	-0.0099
5	2	4	6	←	4	2	3	5	9756.6158	-0.0099
5	2	4	5	←	4	2	3	4	9756.6158	-0.0099
5	2	3	4	←	4	2	2	3	9775.2676	0.0006
5	2	3	6	←	4	2	2	5	9775.2676	0.0006
5	2	3	5	←	4	2	2	4	9775.3890	0.0152
5	1	4	6	←	4	1	3	5	9890.7789	-0.0028
6	1	6	5	←	5	1	5	4	11539.1996	-0.0022
6	1	6	6	←	5	1	5	5	11539.1996	-0.0022
6	1	6	7	←	5	1	5	6	11539.1996	-0.0022
6	0	6	6	←	5	0	5	5	11677.5475	0.0008
6	0	6	5	←	5	0	5	6	11677.5475	0.0008
6	0	6	7	←	5	0	5	6	11677.5475	0.0008
6	0	6	5	←	5	0	5	4	11677.5475	0.0008
6	2	5	6	←	5	2	4	5	11706.0515	0.0049
6	1	5	7	←	5	1	4	6	11866.0230	0.0119
6	1	5	6	←	5	1	4	5	11866.0230	0.0119
7	1	7	7	←	6	1	6	6	13458.8526	-0.0072
7	1	7	6	←	6	1	6	5	13458.8526	-0.0072
7	0	7	7	←	6	0	6	6	13609.8206	-0.0024

7	0	7	6	←	6	0	6	5	13609.8206	-0.0024
7	2	6	7	←	6	2	5	6	13654.3960	0.0037
7	2	5	6	←	6	2	4	5	13706.0234	-0.0072
7	2	5	7	←	6	2	4	6	13706.0234	-0.0072

8.3. Prolinol-$(H_2O)_2$

Table S36. Measured frequencies (ν_{obs}) and residuals ($\nu_{obs} - \nu_{cal}$) of the rotational transitions of isomer 2w1 of prolinol-$(H_2O)_2$.

J'	*K'*$_{-1}$	*K'*$_{+1}$	*F*	←	*J''*	*K''*$_{-1}$	*K''*$_{+1}$	*F''*	ν_{obs} /MHz	ν_{obs}- ν_{calc}/MHz
2	1	2	2	←	1	1	1	1	3084.6012	-0.0124
2	1	2	3	←	1	1	1	2	3084.8318	0.0080
2	0	2	3	←	1	0	1	2	3262.7159	0.0026
2	0	2	1	←	1	0	1	1	3263.0205	-0.0156
2	1	1	1	←	1	1	0	1	3499.7338	-0.0120
2	1	1	2	←	1	1	0	1	3499.8536	-0.0019
2	1	1	3	←	1	1	0	2	3500.0272	-0.0154
2	1	1	1	←	1	1	0	0	3500.3792	-0.0108
3	2	2	2	←	3	1	3	2	3883.8968	0.0039
3	2	2	4	←	3	1	3	4	3884.0116	-0.0033
3	2	2	3	←	3	1	3	3	3884.3585	-0.0050
3	0	3	3	←	2	1	2	2	4024.0540	0.0009
3	0	3	4	←	2	1	2	3	4024.0540	0.0009
2	1	2	2	←	1	0	1	2	4062.0029	0.0016
2	1	2	3	←	1	0	1	2	4062.2610	-0.0163
2	1	2	1	←	1	0	1	1	4062.6198	-0.0028
3	0	3	4	←	2	0	2	3	4823.6248	0.0031
5	3	2	4	←	5	2	3	4	4836.1137	-0.0106
5	3	2	6	←	5	2	3	6	4836.1137	-0.0106
5	2	4	6	←	5	1	5	6	4886.5567	0.0035
5	2	4	5	←	5	1	5	5	4886.8333	0.0114
3	2	2	3	←	2	2	1	2	4938.4300	-0.0078
3	2	2	4	←	2	2	1	3	4938.6362	-0.0071
3	2	2	2	←	2	2	1	1	4938.7740	0.0165
3	2	1	3	←	2	2	0	2	5053.4365	0.0043
3	2	1	4	←	2	2	0	3	5053.5839	-0.0097
3	2	1	2	←	2	2	0	1	5053.7152	0.0167
4	3	1	3	←	4	2	2	3	5106.7967	0.0059
4	3	1	5	←	4	2	2	5	5106.7967	0.0059
3	1	2	3	←	2	1	1	2	5229.8917	-0.0105
3	1	2	4	←	2	1	1	3	5229.8917	-0.0105
3	1	3	3	←	2	0	2	3	5408.9707	-0.0150
3	1	3	3	←	2	0	2	2	5409.2019	-0.0194
3	1	3	2	←	2	0	2	1	5409.3270	-0.0044
3	1	3	4	←	2	0	2	3	5409.3270	-0.0044
3	1	3	2	←	2	0	2	2	5409.7055	0.0137
3	3	1	3	←	3	2	2	3	5423.8107	-0.0099
5	3	3	6	←	5	2	4	6	5641.9627	0.0015
5	3	3	6	←	5	2	4	5	5642.0861	-0.0273
6	2	4	5	←	5	3	3	6	5669.6130	-0.0094
6	2	4	6	←	5	3	3	5	5669.6130	-0.0094
6	2	4	7	←	5	3	3	6	5669.6130	-0.0094
4	0	4	4	←	3	1	3	4	5732.3085	0.0033
4	0	4	5	←	3	1	3	4	5732.6556	0.0056
4	0	4	4	←	3	1	3	3	5732.6556	0.0056
4	0	4	3	←	3	1	3	3	5733.0752	-0.0084
8	2	7	7	←	8	1	8	7	7148.7805	0.0026
6	4	2	7	←	6	3	3	6	7310.6604	-0.0070
6	4	2	5	←	6	3	3	6	7310.6604	-0.0070
5	0	5	5	←	4	1	4	5	7376.5307	0.0038
5	0	5	6	←	4	1	4	5	7376.9212	0.0037
5	4	1	6	←	5	3	2	5	7440.1169	0.0015
5	4	1	4	←	5	3	2	4	7440.1169	0.0015
5	4	1	5	←	5	3	2	5	7440.2753	0.0111
5	1	5	5	←	4	1	4	4	7606.7253	0.0018
5	1	5	4	←	4	1	4	3	7606.7253	0.0018
5	1	5	6	←	4	1	4	5	7606.7253	0.0018
5	0	5	6	←	4	0	4	5	7761.6195	0.0168

5	0	5	4	←	4	0	4	3	7761.6195	0.0168
3	2	1	4	←	2	1	1	3	8015.6385	-0.0031
3	2	1	3	←	2	1	1	2	8015.6385	-0.0031
3	2	1	2	←	2	1	1	1	8015.6385	-0.0031
6	1	5	5	←	5	2	4	4	8344.2731	-0.0075
6	1	5	7	←	5	2	4	6	8344.2731	-0.0075
5	2	3	4	←	4	2	2	4	8632.6764	0.0004
5	2	3	5	←	4	2	2	4	8632.6764	0.0004
5	2	3	6	←	4	2	2	5	8632.6764	0.0004
5	2	3	4	←	4	2	2	3	8632.6764	0.0004
5	2	3	5	←	4	2	2	5	8632.6764	0.0004
3	2	1	2	←	2	1	2	1	8638.2494	0.0041
3	2	1	4	←	2	1	2	3	8638.4294	0.0048
3	2	1	3	←	2	1	2	2	8638.7771	0.0023
6	0	6	5	←	5	1	5	4	8952.1520	0.0090
6	0	6	5	←	5	1	5	4	8952.1520	0.0090
6	0	6	6	←	5	1	5	5	8952.1520	0.0090
6	0	6	7	←	5	1	5	6	8952.1520	0.0090
6	1	6	6	←	5	1	5	5	9079.8263	0.0005
6	1	6	5	←	5	1	5	4	9079.8263	0.0005
6	1	6	7	←	5	1	5	6	9079.8263	0.0005
4	2	3	4	←	3	1	2	3	9202.5534	0.0141
4	2	3	5	←	3	1	2	4	9202.6403	-0.0026
6	1	6	5	←	5	0	5	4	9309.6584	0.0033
6	1	6	7	←	5	0	5	6	9309.6584	0.0033
6	5	2	7	←	6	4	3	7	9645.4640	-0.0018
6	5	2	6	←	6	4	3	6	9645.5985	0.0017
7	1	6	6	←	6	2	5	5	10314.1508	0.0055
7	1	6	6	←	6	2	5	7	10314.1508	0.0055
3	3	0	2	←	2	2	1	2	10364.5658	-0.0074
3	3	0	4	←	2	2	1	3	10364.8858	0.0046
3	3	0	2	←	2	2	1	1	10364.8858	0.0046
3	3	0	3	←	2	2	1	3	10365.1568	0.0037
6	2	4	6	←	5	2	3	5	10433.1338	-0.0051
6	2	4	6	←	5	2	3	5	10433.1338	-0.0051
6	2	4	7	←	5	2	3	6	10433.1338	-0.0026
5	2	4	5	←	4	1	3	5	10435.6666	-0.0026
5	2	4	5	←	4	1	3	4	10435.6666	-0.0016
5	2	4	6	←	4	1	3	5	10435.7986	0.0075
5	2	4	4	←	4	1	3	3	10435.7986	-0.0155
4	2	3	5	←	3	1	3	4	10445.5753	-0.0118
4	2	3	4	←	3	1	3	3	10445.8427	0.0017
7	0	7	7	←	6	1	6	6	10472.7436	0.0009
7	0	7	6	←	6	1	6	5	10472.7436	0.0009
7	1	7	7	←	6	1	6	6	10540.0663	-0.0040
7	1	7	6	←	6	1	6	5	10540.0663	-0.0040
7	0	7	7	←	6	0	6	6	10600.4239	0.0082
7	0	7	6	←	6	0	6	5	10600.4239	0.0082
7	1	7	7	←	6	0	6	6	10667.7631	0.0197
7	1	7	6	←	6	0	6	5	10667.7631	0.0197
4	2	2	3	←	3	1	3	2	10856.2616	0.0035
4	2	2	5	←	3	1	3	4	10856.3978	0.0089
4	2	2	4	←	3	1	3	4	10856.3978	0.0089
4	2	2	4	←	3	1	3	3	10856.7743	0.0093
6	2	5	6	←	5	1	4	5	11590.1608	-0.0013
6	2	5	7	←	5	1	4	6	11590.2957	0.0020
6	2	5	5	←	5	1	4	4	11590.2957	0.0020
8	0	8	7	←	7	1	7	6	11957.0571	-0.0095
8	1	8	7	←	7	0	7	6	12058.5928	-0.0135
4	3	1	3	←	3	2	2	2	12079.1681	0.0072
4	3	1	5	←	3	2	2	4	12079.1681	0.0072
7	2	6	7	←	6	1	5	6	12700.7138	0.0024
7	2	6	6	←	6	1	5	5	12700.8391	-0.0014
5	2	3	6	←	4	1	4	5	13371.7323	0.0013
5	2	3	5	←	4	1	4	4	13372.1309	-0.0068

5	3	3	4	←	4	2	2	4	13396.1929	-0.0011
5	3	3	5	←	4	2	2	4	13396.1929	-0.0011
5	3	3	6	←	4	2	2	5	13396.1929	-0.0011
5	3	3	4	←	4	2	2	3	13396.1929	-0.0011
5	3	2	4	←	4	2	3	3	13879.5851	0.0105
5	3	2	5	←	4	2	3	4	13879.7188	-0.0104

Table S37. Measured frequencies (ν_{obs}) and residuals ($\nu_{obs} - \nu_{cal}$) of the rotational transitions of isomer 2w5 of prolinol-(H_2O)$_2$.

J'	K'_{-1}	K'_{+1}	F	←	J''	K''_{-1}	K''_{+1}	F''	ν_{obs} /MHz	ν_{obs}- ν_{calc}/MHz
2	1	2	1	←	1	1	1	0	3156.3023	0.0022
2	1	2	3	←	1	1	1	2	3156.6277	-0.0011
2	1	2	2	←	1	1	1	1	3156.8774	-0.0036
2	0	2	1	←	1	0	1	1	3317.9314	-0.0015
2	0	2	3	←	1	0	1	2	3318.3147	-0.0051
2	0	2	1	←	1	0	1	0	3318.5661	0.0055
2	0	2	2	←	1	0	1	2	3318.5661	0.0055
2	1	1	1	←	1	1	0	0	3528.8184	-0.0050
2	1	1	3	←	1	1	0	2	3529.0045	-0.0110
2	1	1	2	←	1	1	0	2	3529.2702	0.0040
2	1	1	2	←	1	1	0	1	3529.2702	0.0040
2	1	2	2	←	1	0	1	1	4117.2005	-0.0124
2	1	2	3	←	1	0	1	2	4117.4047	-0.0045
2	1	2	1	←	1	0	1	0	4117.7477	-0.0055
3	0	3	2	←	2	1	2	1	4119.7407	0.0008
3	0	3	4	←	2	1	2	3	4119.7407	0.0008
3	0	3	3	←	2	1	2	2	4119.9390	0.0008
4	1	3	3	←	3	2	2	2	4382.9877	-0.0009
4	1	3	5	←	3	2	2	4	4382.9877	-0.0009
4	1	3	4	←	3	2	2	3	4383.3177	-0.0089
6	3	3	6	←	6	2	4	6	4485.7940	-0.0003
6	3	3	7	←	6	2	4	7	4485.9358	-0.0116
5	2	4	4	←	5	1	5	4	4630.5327	0.0013
5	2	4	6	←	5	1	5	6	4630.5327	0.0013
5	2	4	5	←	5	1	5	5	4630.5327	0.0013
3	1	3	4	←	2	1	2	3	4720.5430	0.0085
3	0	3	2	←	2	0	2	2	4918.5114	-0.0039
3	0	3	3	←	2	0	2	2	4918.8410	0.0012
3	0	3	4	←	2	0	2	3	4918.8410	0.0012
3	0	3	3	←	2	0	2	3	4919.0717	-0.0082
3	2	2	2	←	2	2	1	1	5014.1041	-0.0014
3	2	2	4	←	2	2	1	3	5014.2485	-0.0049
3	2	2	3	←	2	2	1	2	5014.5127	-0.0071
4	3	1	4	←	4	2	2	4	5016.8547	-0.0034
4	3	1	5	←	4	2	2	5	5017.1376	-0.0075
4	3	1	3	←	4	2	2	3	5017.1376	-0.0075
3	2	1	2	←	2	2	0	1	5109.5145	-0.0029
3	2	1	4	←	2	2	0	3	5109.6627	-0.0079
3	2	1	3	←	2	2	0	2	5109.9548	-0.0076
3	3	0	3	←	3	2	1	3	5165.0858	-0.0066
3	3	0	4	←	3	2	1	4	5165.4834	0.0095
3	3	0	2	←	3	2	1	2	5165.6127	0.0052
6	2	5	5	←	6	1	6	5	5227.3840	-0.0134
6	2	5	7	←	6	1	6	7	5227.3840	-0.0191
3	1	2	2	←	2	1	1	2	5276.7524	-0.0034
3	1	2	4	←	2	1	1	3	5277.0810	0.0053
3	1	2	2	←	2	1	1	1	5277.0810	0.0053
3	1	2	3	←	2	1	1	3	5277.3488	-0.0056

3	3	1	3	←	3	2	2	3	5283.0337	-0.0047
3	3	1	4	←	3	2	2	4	5283.3666	-0.0119
3	3	1	2	←	3	2	2	2	5283.5007	0.0031
4	3	2	4	←	4	2	3	4	5345.8989	-0.0033
4	3	2	5	←	4	2	3	5	5346.0881	-0.0096
5	3	3	5	←	5	2	4	5	5465.5160	-0.0112
5	3	3	6	←	5	2	4	6	5465.6491	-0.0034
5	3	3	4	←	5	2	4	4	5465.6491	-0.0034
3	1	3	3	←	2	0	2	2	5519.4835	-0.0094
3	1	3	4	←	2	0	2	3	5519.6181	-0.0058
6	3	4	6	←	6	2	5	6	5660.3317	0.0037
6	3	4	7	←	6	2	5	7	5660.3317	0.0037
6	3	4	5	←	6	2	5	5	5660.3317	0.0037
6	2	4	7	←	5	3	3	6	5788.5302	0.0158
6	2	4	6	←	5	3	3	5	5788.7574	-0.0137
4	0	4	3	←	3	1	3	2	5861.5381	-0.0048
4	0	4	5	←	3	1	3	4	5861.5381	-0.0048
7	3	5	6	←	7	2	6	6	5945.1207	0.0014
4	1	3	3	←	3	1	2	3	7002.0330	0.0014
4	1	3	5	←	3	1	2	4	7002.4113	0.0026
4	1	3	3	←	3	1	2	2	7002.4113	0.0026
4	1	3	4	←	3	1	2	3	7002.4113	0.0026
4	1	3	4	←	3	1	2	4	7002.7073	0.0027
6	4	2	6	←	6	3	3	6	7168.8254	-0.0025
6	4	2	6	←	6	3	3	5	7169.0068	0.0050
4	4	0	4	←	4	3	1	4	7323.0939	0.0028
6	4	3	6	←	6	3	4	6	7323.0939	0.0028
6	4	3	5	←	6	3	4	5	7323.2646	-0.0032
4	4	0	5	←	4	3	1	5	7323.4068	0.0119
4	4	1	4	←	4	3	2	4	7337.0867	-0.0078
4	4	1	5	←	4	3	2	5	7337.4212	-0.0079
4	4	1	3	←	4	3	2	3	7337.4212	-0.0079
5	0	5	6	←	4	1	4	5	7549.8784	-0.0109
5	0	5	4	←	4	1	4	3	7549.8784	-0.0086
5	1	5	6	←	4	1	4	5	7803.3226	-0.0007
5	1	5	4	←	4	1	4	3	7803.3226	-0.0007
5	1	5	5	←	4	1	4	4	7803.3226	-0.0007
3	2	2	3	←	2	1	1	2	7896.2358	0.0024
3	2	2	4	←	2	1	1	3	7896.4491	0.0016
3	2	2	2	←	2	1	1	1	7896.5734	0.0069
5	0	5	4	←	4	0	4	4	7957.8465	0.0015
5	0	5	5	←	4	0	4	4	7958.0979	0.0054
5	0	5	6	←	4	0	4	5	7958.0979	0.0054
5	0	5	4	←	4	0	4	3	7958.0979	0.0054
5	0	5	5	←	4	0	4	5	7958.2849	-0.0006
3	2	1	3	←	2	1	1	2	8016.2264	0.0049
3	2	1	4	←	2	1	1	3	8016.3958	0.0026
3	2	1	2	←	2	1	1	1	8016.5008	0.0036
5	1	5	5	←	4	0	4	4	8211.5114	0.0002
5	1	5	6	←	4	0	4	5	8211.5114	0.0002
5	1	5	4	←	4	0	4	3	8211.5114	0.0002
5	2	4	4	←	4	2	3	3	8302.6048	-0.0054
5	2	4	6	←	4	2	3	5	8302.6048	-0.0054
5	4	2	6	←	4	4	1	5	8411.6079	-0.0035
5	4	2	5	←	4	4	1	4	8411.8489	0.0073
5	4	1	6	←	4	4	0	5	8412.7185	-0.0094
5	4	1	5	←	4	4	0	4	8412.9568	-0.0017
5	3	3	6	←	4	3	2	5	8422.1584	0.0011
5	3	3	5	←	4	3	2	4	8422.2947	0.0055
6	1	5	5	←	5	2	4	4	8441.1293	0.0024
6	1	5	7	←	5	2	4	6	8441.1293	0.0024
6	1	5	6	←	5	2	4	5	8441.2777	0.0013
3	2	2	4	←	2	1	2	3	8455.0110	0.0005
5	3	2	6	←	4	3	1	5	8463.4729	0.0076
5	3	2	5	←	4	3	1	4	8463.6085	0.0031

3	2	1	3	←	2	1	2	2	8574.9562	0.0001
3	2	1	4	←	2	1	2	3	8574.9562	0.0001
3	2	1	2	←	2	1	2	1	8574.9562	0.0001
5	1	4	4	←	4	1	3	4	8692.5241	0.0021
5	1	4	6	←	4	1	3	5	8692.9023	0.0026
5	1	4	5	←	4	1	3	4	8692.9023	0.0026
5	1	4	4	←	4	1	3	3	8692.9023	0.0026
5	1	4	5	←	4	1	3	5	8693.2118	0.0053
5	2	3	4	←	4	2	2	4	8700.1577	0.0084
5	2	3	4	←	4	2	2	3	8700.3993	-0.0059
5	2	3	6	←	4	2	2	5	8700.3993	-0.0059
5	2	3	5	←	4	2	2	4	8700.3993	-0.0059
5	2	3	5	←	4	2	2	5	8700.6365	-0.0020
6	0	6	7	←	5	1	5	6	9175.9237	-0.0044
6	0	6	6	←	5	1	5	5	9175.9237	-0.0044
4	2	3	4	←	3	1	2	3	9285.7171	0.0025
4	2	3	5	←	3	1	2	4	9285.9049	0.0141
8	5	3	7	←	8	4	4	7	9287.1329	0.0027
6	1	6	7	←	5	1	5	6	9322.4298	0.0001
6	1	6	6	←	5	1	5	5	9322.4298	0.0001
6	1	6	5	←	5	1	5	4	9322.4298	0.0001
6	0	6	5	←	5	0	5	5	9429.1286	0.0012
6	0	6	6	←	5	0	5	5	9429.3422	-0.0021
6	0	6	7	←	5	0	5	6	9429.3422	-0.0021
6	0	6	5	←	5	0	5	4	9429.3422	-0.0021
6	0	6	6	←	5	0	5	6	9429.5211	0.0000
4	2	2	4	←	3	1	2	3	9628.9205	0.0118
4	2	2	5	←	3	1	2	4	9628.9977	-0.0163
6	2	5	7	←	5	2	4	6	9919.3121	0.0010
6	2	5	5	←	5	2	4	4	9919.3121	0.0010
6	2	5	6	←	5	2	4	5	9919.3121	0.0010
6	4	3	5	←	5	4	2	4	10110.3923	0.0087
6	4	3	7	←	5	4	2	6	10110.3923	0.0087
6	4	3	6	←	5	4	2	5	10110.5388	0.0070
6	3	4	5	←	5	3	3	4	10114.0242	-0.0035
6	3	4	7	←	5	3	3	6	10114.0242	-0.0035
6	3	4	6	←	5	3	3	5	10114.0242	-0.0035
6	4	2	5	←	5	4	1	4	10115.3403	0.0008
6	4	2	7	←	5	4	1	6	10115.3403	0.0008
6	4	2	6	←	5	4	1	5	10115.4923	0.0034
3	3	1	3	←	2	2	0	3	10272.7704	0.0070
3	3	1	3	←	2	2	0	2	10273.0793	0.0091
3	3	1	2	←	2	2	0	1	10273.0793	0.0091
3	3	1	4	←	2	2	0	3	10273.0793	0.0091
3	3	1	2	←	2	2	0	2	10273.4704	-0.0014
3	3	1	4	←	2	2	1	3	10297.6435	0.0115
3	3	0	3	←	2	2	1	3	10299.3391	0.0051
3	3	0	4	←	2	2	1	3	10299.6753	0.0024
3	3	0	2	←	2	2	1	2	10300.0585	0.0005
4	2	3	4	←	3	1	3	3	10400.9950	0.0058
4	2	3	5	←	3	1	3	4	10400.9950	0.0058
4	2	3	3	←	3	1	3	2	10400.9950	0.0058
6	2	4	7	←	5	2	3	6	10513.2542	-0.0114
6	2	4	5	←	5	2	3	4	10513.2542	-0.0114
4	2	2	3	←	3	1	3	2	10744.1134	0.0031
4	2	2	5	←	3	1	3	4	10744.1134	0.0031
7	0	7	6	←	6	1	6	5	10749.3467	-0.0043
7	0	7	7	←	6	1	6	6	10749.3467	-0.0043
7	1	7	7	←	6	1	6	6	10829.7239	-0.0041
7	1	7	6	←	6	1	6	5	10829.7239	-0.0041
7	0	7	7	←	6	0	6	6	10895.8521	0.0006
7	0	7	6	←	6	0	6	5	10895.8521	0.0006
7	1	7	7	←	6	0	6	6	10976.2365	0.0080
7	1	7	6	←	6	0	6	5	10976.2365	0.0080
7	2	6	7	←	6	2	5	6	11514.3331	-0.0047

7	5	2	6	←	6	5	1	5	11788.0804	0.0066
7	5	2	7	←	6	5	1	6	11788.2338	0.0015
7	3	5	6	←	6	3	4	5	11799.0772	-0.0126
6	2	5	6	←	5	1	4	5	11812.3875	-0.0025
6	2	5	7	←	5	1	4	6	11812.5237	-0.0044
6	2	5	5	←	5	1	4	4	11812.5237	-0.0044
4	3	2	4	←	3	2	1	3	11892.5346	-0.0012
4	3	2	5	←	3	2	1	4	11892.6617	-0.0032
7	1	6	7	←	6	1	5	6	11914.8136	0.0047
7	1	6	6	←	6	1	5	5	11914.8136	0.0047
8	0	8	7	←	7	1	7	6	12285.8971	0.0026
7	2	5	6	←	6	2	4	5	12301.8273	0.0029
7	2	5	7	←	6	2	4	6	12301.8273	0.0029
8	1	8	7	←	7	1	7	6	12328.3643	0.0036
8	1	7	7	←	7	2	6	6	12355.5256	0.0013
8	0	8	7	←	7	0	7	6	12366.2723	-0.0056
8	1	8	7	←	7	0	7	6	12408.7391	-0.0051
8	2	7	7	←	7	2	6	6	13086.8782	0.0116
5	2	3	4	←	4	1	4	3	13174.7364	0.0030
5	2	3	6	←	4	1	4	5	13174.7364	0.0030
5	2	3	5	←	4	1	4	4	13174.8572	0.0024
5	3	3	5	←	4	2	2	4	13424.9995	0.0019
5	3	3	6	←	4	2	2	5	13425.1348	0.0029
8	1	7	7	←	7	1	6	6	13433.2125	-0.0030
8	3	6	7	←	7	3	5	6	13471.3946	0.0101
8	4	5	7	←	7	4	4	6	13526.3576	-0.0126
8	4	4	7	←	7	4	3	6	13569.1706	-0.0032
8	3	5	7	←	7	3	4	6	13853.7993	-0.0023

8.4. Prolinol-(H_2O)$_3$

Table S38. Measured frequencies (ν_{obs}) and residuals ($\nu_{obs} - \nu_{cal}$) of the rotational transitions of isomer 3w1 of prolinol-(H_2O)$_3$.

J'	K'_{-1}	K'_{+1}	F	←	J''	K''_{-1}	K''_{+1}	F''	ν_{obs} /MHz	ν_{obs}- ν_{calc}/MHz
2	1	2	2	←	1	0	1	2	3040.0234	-0.0124
2	1	2	2	←	1	0	1	1	3040.3716	-0.0015
2	1	2	3	←	1	0	1	2	3040.5375	-0.0028
2	1	2	1	←	1	0	1	1	3041.1685	0.0106
3	3	0	4	←	3	2	1	4	3228.4816	-0.0057
3	3	0	3	←	3	2	1	3	3228.8288	-0.0128
3	0	3	3	←	2	1	2	3	3282.7141	-0.0020
3	0	3	2	←	2	1	2	1	3283.1053	-0.0168
3	0	3	4	←	2	1	2	3	3283.2285	0.0040
3	0	3	2	←	2	1	2	2	3283.9000	-0.0070
4	3	2	3	←	4	2	3	3	3348.5150	-0.0167
4	3	2	5	←	4	2	3	5	3348.6246	0.0056
4	3	2	4	←	4	2	3	4	3348.9425	-0.0159
5	3	3	4	←	5	2	4	4	3427.8949	-0.0031
5	3	3	6	←	5	2	4	6	3427.8949	-0.0031
5	3	3	5	←	5	2	4	5	3428.1779	-0.0256
6	3	4	6	←	6	2	5	7	3556.7352	0.0213
6	3	4	5	←	6	2	5	5	3556.7352	0.0213
6	3	4	6	←	6	2	5	6	3557.0099	-0.0123
4	1	3	5	←	3	2	2	4	3697.5508	0.0092
4	1	3	3	←	3	2	2	2	3697.5508	0.0092
3	1	3	3	←	2	0	2	3	4146.9315	-0.0014
3	1	3	3	←	2	0	2	2	4147.3439	-0.0043
3	1	3	2	←	2	0	2	1	4147.5629	0.0018
3	1	3	4	←	2	0	2	3	4147.5629	0.0018
3	1	3	2	←	2	0	2	2	4148.2100	0.0075
6	4	2	5	←	6	3	3	5	4476.5544	0.0043
6	4	2	7	←	6	3	3	7	4476.5544	0.0043
6	4	2	6	←	6	3	3	6	4476.6809	0.0083
2	2	1	1	←	1	1	0	1	4479.0741	0.0043
2	2	1	2	←	1	1	0	1	4479.6212	-0.0106
2	2	1	3	←	1	1	0	2	4479.7486	0.0073
2	2	1	2	←	1	1	0	2	4480.1040	0.0013
2	2	1	1	←	1	1	0	0	4480.2445	-0.0024
2	2	0	3	←	1	1	0	2	4496.0843	0.0019
2	2	0	1	←	1	1	0	0	4496.5589	0.0009
5	4	1	6	←	5	3	2	6	4546.5964	0.0109
5	4	1	5	←	5	3	2	5	4546.7980	-0.0043
4	4	0	5	←	4	3	1	5	4580.8662	-0.0010
4	4	0	4	←	4	3	1	4	4581.2165	-0.0133
6	4	3	7	←	6	3	4	7	4582.1290	0.0172
6	4	3	6	←	6	3	4	6	4582.2834	-0.0174
5	4	2	6	←	5	3	3	6	4583.7737	-0.0106
5	4	2	5	←	5	3	3	5	4584.0343	-0.0044
4	0	4	5	←	3	1	3	4	4605.8240	-0.0372
4	0	4	3	←	3	1	3	3	4606.6400	-0.0105
2	2	0	1	←	1	1	1	0	4615.8357	-0.0009
2	2	0	3	←	1	1	1	2	4616.2702	0.0026
2	2	0	2	←	1	1	1	1	4616.8196	0.0031
3	1	2	4	←	2	0	2	3	4867.7091	0.0003
3	1	2	3	←	2	0	2	2	4868.1642	0.0050
6	2	4	7	←	5	3	3	6	5063.1945	-0.0095
6	2	4	6	←	5	3	3	5	5063.1945	-0.0095
5	1	4	4	←	4	2	3	3	5204.8313	-0.0143
5	1	4	6	←	4	2	3	5	5204.8313	-0.0143
5	1	4	5	←	4	2	3	4	5204.8313	-0.0143
4	1	4	4	←	3	0	3	4	5221.5611	0.0013
4	1	4	4	←	3	0	3	3	5222.0629	-0.0053

4	1	4	5	←	3	0	3	4	5222.2666	-0.0024
4	1	4	3	←	3	0	3	2	5222.2666	-0.0024
4	1	4	3	←	3	0	3	3	5222.9620	0.0041
3	2	2	3	←	2	1	1	2	5640.0403	-0.0042
3	2	2	4	←	2	1	1	3	5640.2035	0.0158
5	0	5	6	←	4	1	4	5	5892.8834	0.0031
5	0	5	5	←	4	1	4	4	5892.8834	0.0031
3	3	1	2	←	2	2	0	2	7132.1809	-0.0002
3	3	1	2	←	2	2	0	3	7132.5756	-0.0208
3	3	1	2	←	2	2	0	1	7132.8230	-0.0088
3	3	1	3	←	2	2	0	2	7132.8230	-0.0088
3	3	0	4	←	2	2	1	3	7150.5174	0.0063
3	3	0	3	←	2	2	1	2	7150.6482	0.0104
3	3	0	3	←	2	2	1	3	7151.0029	0.0038
6	1	6	6	←	5	0	5	6	7376.5308	0.0013
6	1	6	6	←	5	0	5	5	7377.2349	0.0067
6	1	6	5	←	5	0	5	4	7377.3425	0.0080
6	1	6	7	←	5	0	5	6	7377.3425	0.0080
4	2	3	3	←	3	1	3	2	7458.6678	0.0001
4	2	3	4	←	3	1	3	4	7458.6678	0.0001
4	2	3	5	←	3	1	3	4	7458.8605	0.0152
4	2	3	3	←	3	1	3	4	7458.8605	0.0152
4	2	2	3	←	3	1	3	2	7686.3877	0.0041
4	2	2	5	←	3	1	3	4	7686.6253	0.0055
4	2	2	3	←	3	1	3	4	7686.6253	0.0055
4	2	2	4	←	3	1	3	3	7687.3073	-0.0033
5	2	4	5	←	4	1	3	5	7779.5230	-0.0079
5	2	4	5	←	4	1	3	4	7779.5230	-0.0079
5	2	4	6	←	4	1	3	5	7779.7487	-0.0162
7	1	6	6	←	6	2	5	5	8195.2211	0.0080
7	0	7	7	←	6	1	6	6	8353.2748	0.0046
7	0	7	6	←	6	1	6	5	8353.2748	0.0046
4	3	2	5	←	3	2	1	4	8378.8710	-0.0035
4	3	2	3	←	3	2	1	2	8378.8710	-0.0035
4	3	1	5	←	3	2	1	4	8388.5964	-0.0050
4	3	1	5	←	3	2	2	4	8468.4478	0.0248
4	3	1	4	←	3	2	2	3	8468.6203	0.0098
7	1	7	6	←	6	0	6	5	8486.1153	-0.0059
6	2	5	6	←	5	1	4	5	8773.6607	0.0039
6	2	5	7	←	5	1	4	6	8773.8834	-0.0123
8	0	8	7	←	7	1	7	6	9544.1448	-0.0017
7	2	6	7	←	6	1	5	6	9739.4759	0.0182
7	2	6	6	←	6	1	5	6	9739.9321	0.0107
4	4	1	5	←	3	3	0	4	9740.8758	-0.0055
4	4	1	4	←	3	3	0	4	9741.4401	0.0088
4	4	0	3	←	3	3	1	3	9741.7622	0.0026
4	4	0	5	←	3	3	1	4	9742.3904	0.0049
4	4	0	4	←	3	3	1	4	9742.9503	0.0146
5	3	2	4	←	4	2	3	5	9833.2426	0.0027
5	3	2	6	←	4	2	3	5	9833.2426	0.0027
5	3	2	5	←	4	2	3	4	9833.4532	-0.0063
6	3	4	6	←	5	2	3	5	10678.0318	-0.0099
6	3	4	7	←	5	2	3	6	10678.1732	0.0023
6	3	4	5	←	5	2	3	6	10678.1732	0.0023
6	3	4	5	←	5	2	3	4	10678.1732	0.0023
8	2	7	7	←	7	1	6	6	10702.8643	-0.0050
5	4	2	4	←	4	3	1	3	11020.6027	0.0196
5	4	2	6	←	4	3	1	5	11020.6027	0.0196
5	4	1	4	←	4	3	2	3	11031.2114	0.0092
5	4	1	6	←	4	3	2	5	11031.2114	0.0092
7	3	5	7	←	6	2	4	6	11706.3222	-0.0051
7	3	5	6	←	6	2	4	6	11706.4920	-0.0004
6	4	3	5	←	5	3	2	4	12284.3250	-0.0294
6	4	3	7	←	5	3	2	6	12284.3250	-0.0294
6	4	2	7	←	5	3	3	6	12326.8658	0.0052

6	5	2	5	←	5	4	1	4	13626.0023	-0.0023
6	5	2	7	←	5	4	1	6	13626.0023	-0.0023
6	5	1	7	←	5	4	2	6	13626.9703	0.0014
6	5	1	7	←	5	4	2	6	13626.9703	0.0014
6	5	1	6	←	5	4	2	5	13626.9703	0.0014

9. Conformational analysis of (S)-prolinol in solution

Table S39. Comparison of the Experimental and MD Simulation Derived Distances for (*S*)-prolinol in aqueous solution (pH 10.0).

Distance	Experimental data	Unrestrained 1 μs MD simulations
A/B-D	< 2.5	2.7
A/B-E	< 3.5	3.1
A/B-I	< 3.5	4.4
A/B-F	< 3.5	4.3
C-E	< 2.5	2.3
C-H	< 3.5	2.8
C-G	< 3.5	3.1
H-E	< 3.5	3.5
I-D	< 3.5	3.5

Table S40. Comparison of the Experimental and MD Simulation Derived Distances for (*S*)-prolinol in in methanol.

Distance	Experimental data	Unrestrained 1 μs MD simulations
A/B-D	< 2.5	2.7
A/B-E	< 3.5	3.1
A/B-I	< 3.5	4.3
A/B-F	< 3.5	4.3
C-E	< 2.5	2.3
C-H	< 3.5	2.8
C-G	< 3.5	3.1
H-E	< 3.5	3.5
I-D	< 3.5	3.5

Figure S10. Top: ^{1}H NMR spectrum of (*S*)-prolinol in D_2O (600 MHz, pH 10, 25 °C), together with labels employed for the different proton signals. Bottom left: 2D NOESY spectrum acquired under identical conditions with a mixing time of 600 ms. Key cross-peaks employed to derive upper limits for the interproton distances are highlighted. Bottom right: Schematic representation of (S)-prolinol indicating the relevant NOE-derived distances and the $^{3}J_{H,H}$ coupling constants (in Hz) obtained from the NMR spectra.

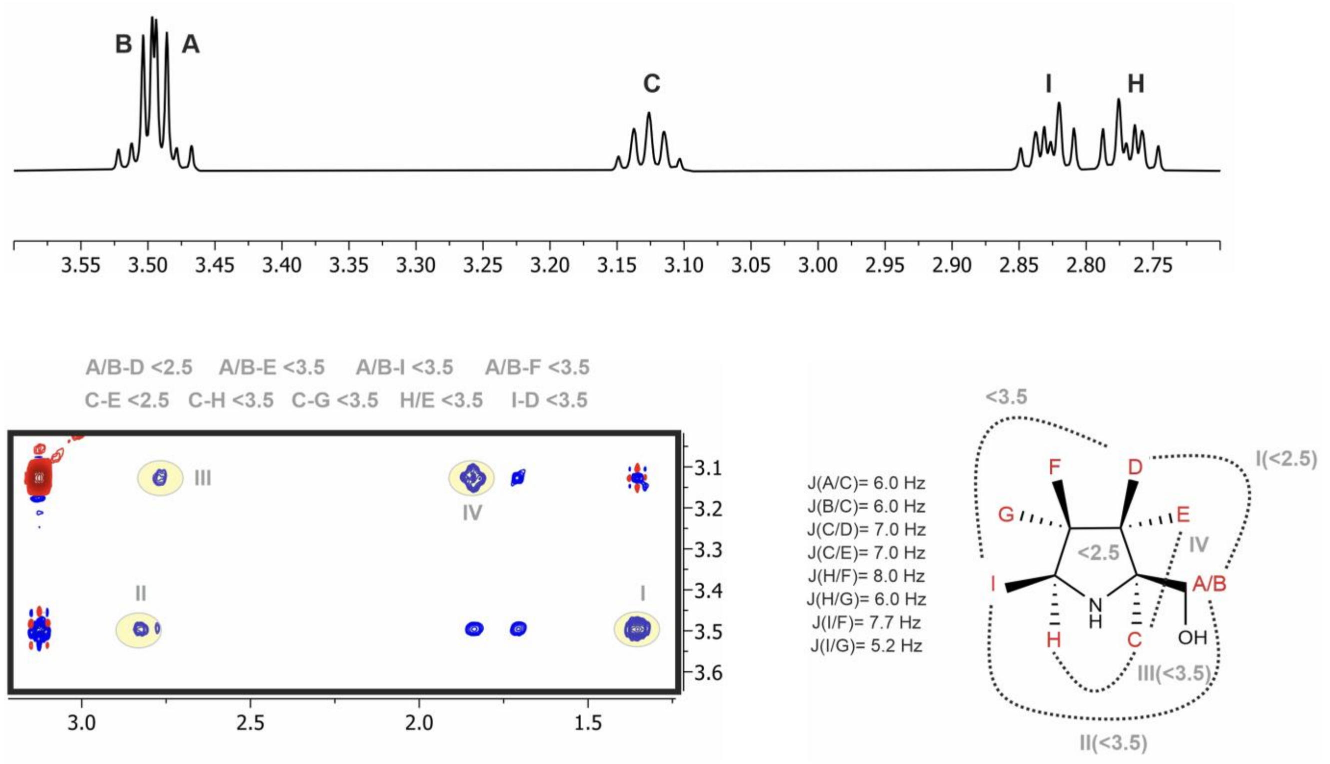

Figure S11. Top: ^{1}H NMR spectrum of (*S*)-prolinol in CD_3OD (600 MHz, 25 °C), together with labels employed for the different proton signals. Bottom left: 2D NOESY spectrum acquired under identical conditions with a mixing time of 600 ms. Key cross-peaks employed to derive upper limits for the interproton distances are highlighted. Bottom right: Schematic representation of (S)-prolinol indicating the relevant NOE-derived distances and the $^3J_{H,H}$ coupling constants (in Hz) obtained from the NMR spectra.

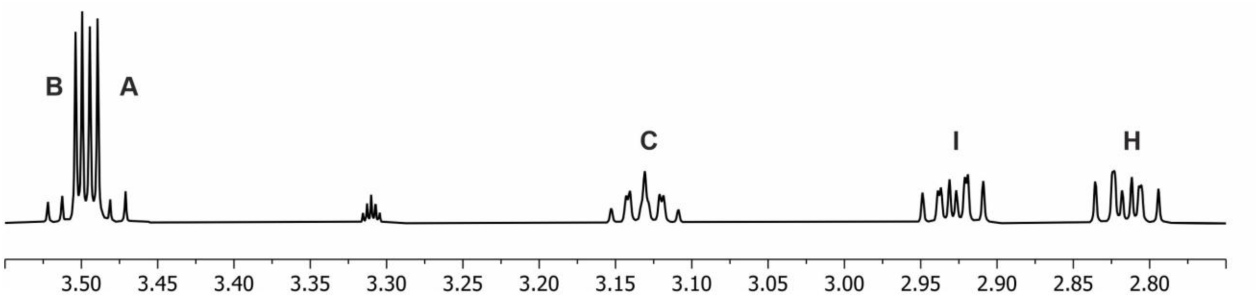

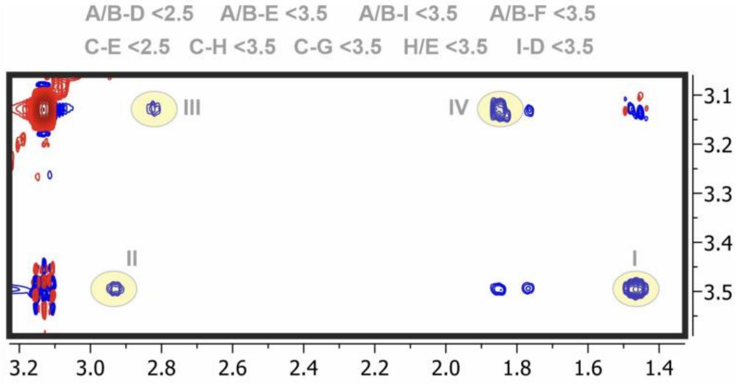

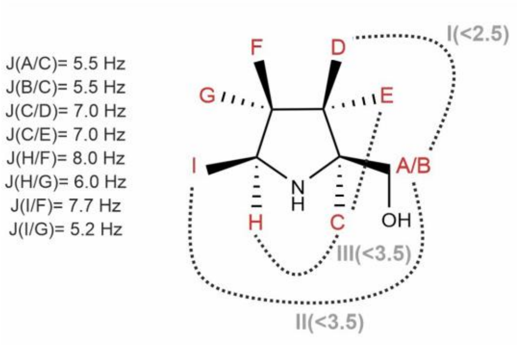

Figure S12. Cremer–Pople ring-puckering analysis of prolinol conformers obtained from 1 μs MD simulations in aqueous solution (left) and in methanol (right). The parameter *q* denotes the puckering amplitude (in Å), while Φ represents the puckering phase (in degrees).

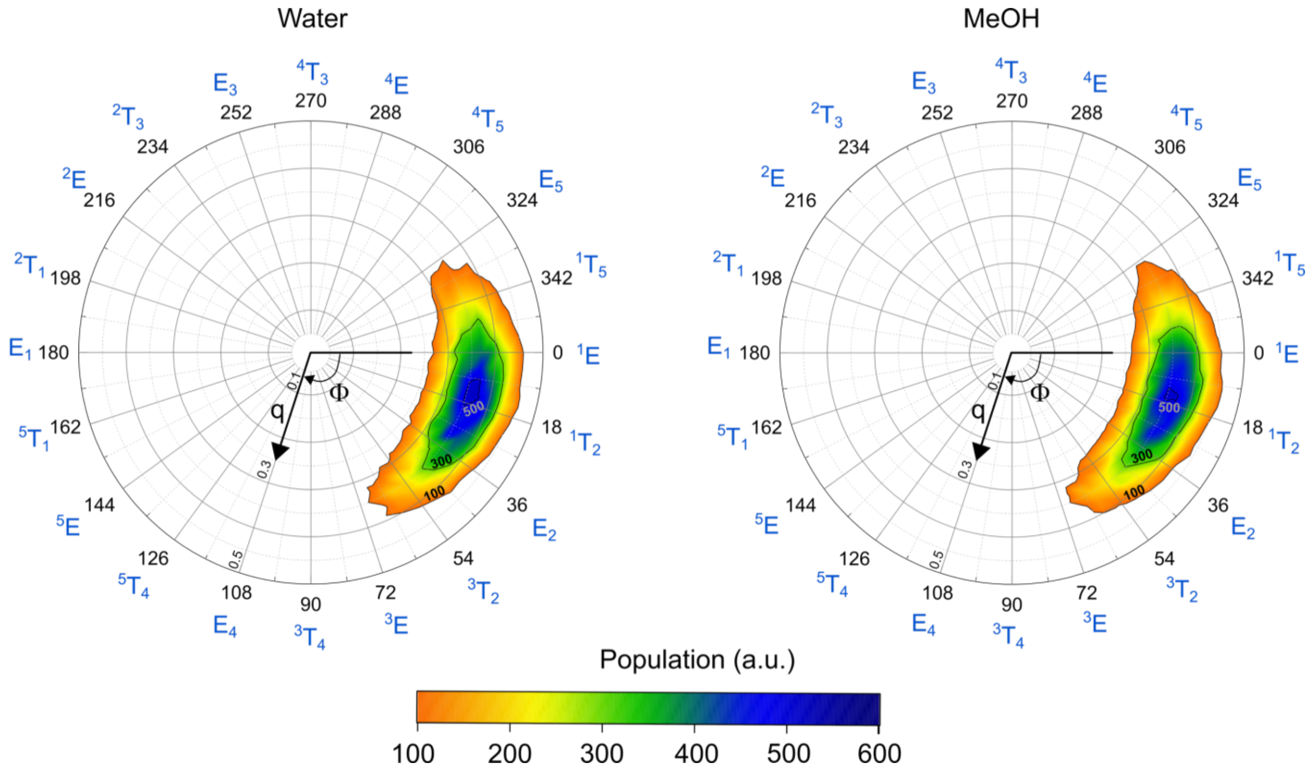

Figure S13. Top: Oxygen density maps of the first solvation shell around (*S*)-prolinol in water from 1 μs MD simulations, shown together with the average molecular geometry. The corresponding distribution of the dihedral angle ω (N1–C2–C6–O) is displayed on the right. Bottom: Equivalent oxygen density maps and ω distribution obtained from 1 μs MD simulations in methanol. Violin plots depict the full angular distribution; the central solid line denotes the median, while the dotted lines indicate the interquartile range.

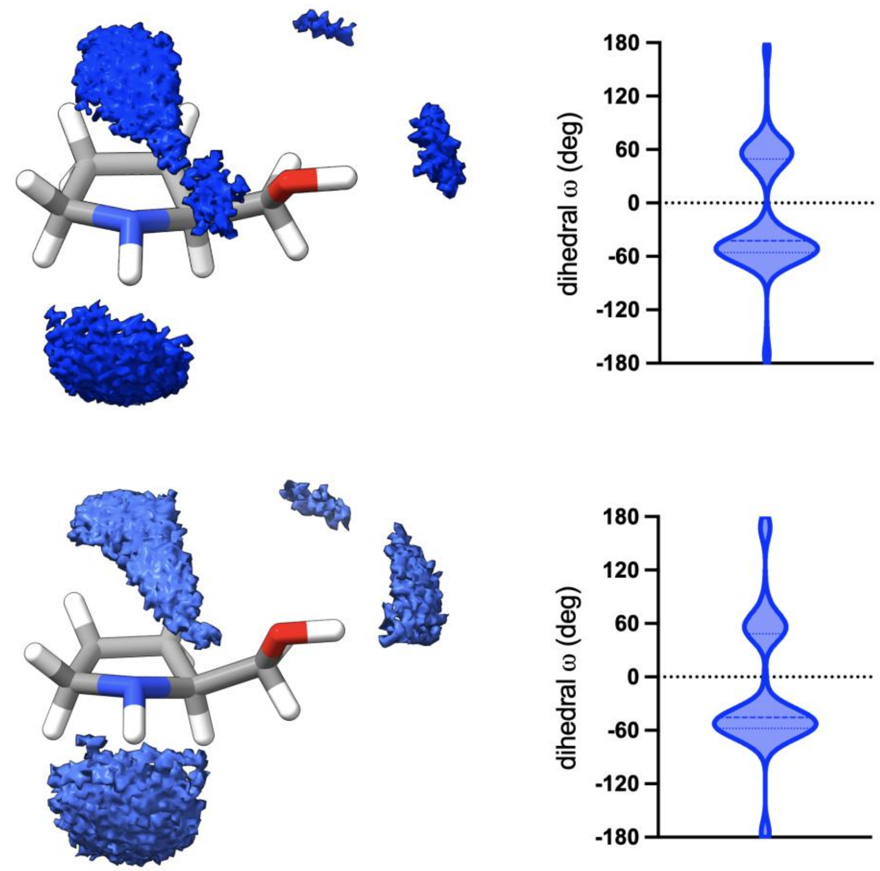

Supplement: Supplementary file 1 [file ja5c13582_si_001.pdf]
